# Towards the controlled enzymatic synthesis of LNA containing oligonucleotides

**DOI:** 10.3389/fchem.2023.1161462

**Published:** 2023-04-27

**Authors:** Nazarii Sabat, Dace Katkevica, Karlis Pajuste, Marie Flamme, Andreas Stämpfli, Martins Katkevics, Steven Hanlon, Serena Bisagni, Kurt Püntener, Filippo Sladojevich, Marcel Hollenstein

**Affiliations:** ^1^ Institut Pasteur, Université de Paris Cité, CNRS UMR3523, Department of Structural Biology and Chemistry, Laboratory for Bioorganic Chemistry of Nucleic Acids, Paris, France; ^2^ Latvian Institute of Organic Synthesis, Riga, Latvia; ^3^ Pharma Research and Early Development, Roche Innovation Center Basel, F Hoffmann-La Roche Ltd., Basel, Switzerland; ^4^ Pharmaceutical Division, Synthetic Molecules Technical Development, Process Development and Catalysis, F Hoffmann-La Roche Ltd., Basel, Switzerland

**Keywords:** locked nucleic acids (LNA), XNA, modified nucleotides, polymerases, controlled enzymatic synthesis

## Abstract

Enzymatic, *de novo* XNA synthesis represents an alternative method for the production of long oligonucleotides containing chemical modifications at distinct locations. While such an approach is currently developed for DNA, controlled enzymatic synthesis of XNA remains at a relative state of infancy. In order to protect the masking groups of 3′-*O*-modified LNA and DNA nucleotides against removal caused by phosphatase and esterase activities of polymerases, we report the synthesis and biochemical characterization of nucleotides equipped with ether and robust ester moieties. While the resulting ester-modified nucleotides appear to be poor substrates for polymerases, ether-blocked LNA and DNA nucleotides are readily incorporated into DNA. However, removal of the protecting groups and modest incorporation yields represent obstacles for LNA synthesis via this route. On the other hand, we have also shown that the template-independent RNA polymerase PUP represents a valid alternative to the TdT and we have also explored the possibility of using engineered DNA polymerases to increase substrate tolerance for such heavily modified nucleotide analogs.

## 1 Introduction

Synthetic oligonucleotides play essential roles in an increasing number of applications including storage of digital information in DNA (Lee et al., 2019; Doricchi et al., 2022), drug discovery (Lindenburg et al., 2020; Vummidi et al., 2022), and the development of mRNA vaccines (Jackson et al., 2020; Chaudhary et al., 2021). Besides the need for production of larger numbers of sequences and scaling up to kilograms, demands vary widely in terms of size and also in sequence and chemical composition. For instance, antisense oligonucleotides consist of short, fully-modified sequences and the *de novo* genome synthesis requires the error-free assembly of massive amounts of shorter stretches of unmodified DNA (Masaki et al., 2022; Matthey-Doret et al., 2022). On the other end of the spectrum, mRNA vaccines require the production of long (several thousands of nucleotides) oligonucleotides containing modified residues such as *N*1-methyl-pseudouridine (Nance and Meier, 2021; Dousis et al., 2022) while studies aiming at understanding the mechanisms and functions of larger RNAs such as long non-coding RNAs or mRNA call in for the synthesis of long, heavily modified sequences (Zuckerman et al., 2020; Statello et al., 2021; Liu and Wang, 2022).

The main approach for the synthesis of oligonucleotides relies on the iterative addition of phosphoramidite-based building blocks on immobilized nucleic acid sequences (Beaucage and Caruthers, 1981; Caruthers, 1985). While this method has met undeniable success, there are still inherently limiting factors. For instance, sequences longer than 200 nucleotides cannot be obtained by this solid-phase synthetic approach. In addition, the sustainability (Andrews et al., 2021) as well as the scalability (Molina and Sanghvi, 2019) of phosphorous (III)-based oligonucleotide synthesis are limited which negatively impacts scalable manufacturing (Van Giesen et al., 2023). Hence, various enzymatic methods are currently developed to alleviate the shortcomings of solid-phase synthesis of nucleic acids. In this context, controlled enzymatic synthesis represents a promising approach where temporarily blocked nucleoside triphosphates are incorporated sequentially into DNA mainly by template-independent polymerases such as the terminal deoxynucleotidyl transferase (TdT) (Jensen and Davis, 2018; Lee et al., 2019; Sarac and Hollenstein, 2019; Doricchi et al., 2022; Lu et al., 2022; Wang et al., 2022; Ashley et al., 2023; Hoose et al., 2023; Van Giesen et al., 2023). The blocking groups can be affixed either at the 3′-hydroxyl moiety to prevent further nucleophilic attack on the α-phosphorous of incoming nucleoside triphosphates (Bollum, 1962; Mackey and Gilham, 1971; Chen et al., 2010; Hutter et al., 2010; Gardner et al., 2012; Chen et al., 2013; Mathews et al., 2016; Jang et al., 2019) or on the nucleobase which then act as inhibitors of polymerases (Bowers et al., 2009; Palluk et al., 2018). While robust protocols have been established for DNA (Palluk et al., 2018; Lee et al., 2019; Jung et al., 2022; Venter et al., 2022; Wang et al., 2022), changing the sugar chemistry to ribose (RNA) or to more complex modification patterns deviating from natural systems (xenonucleic acids, XNAs(Chaput and Herdewijn, 2019; Chaput et al., 2020)) raises yet unmet challenges.

We have recently explored the possibility of using phosphate (Flamme et al., 2022a) or robust ester functionalities (Flamme et al., 2022b) as prosthetic 3′-*O*-protecting groups for controlled enzymatic synthesis of locked nucleic acids (LNAs). While some of these temporarily blocked XNA nucleotides are tolerated by various polymerases including TdT, intrinsic esterase (Canard et al., 1995; LinWu et al., 2019; LinWu et al., 2020) and phosphatase (Krayevsky et al., 2000; Flamme et al., 2022a) activities of polymerases precludes their use for the crafting of oligonucleotides. Here, we have explored i) the possibility of using yet more robust protecting groups designed to resist esterase and phosphatase activity for DNA and XNA synthesis with template-dependent and independent polymerases, ii) whether other template-independent polymerases than the TdT could be harnessed for *de novo* DNA and LNA synthesis, and iii) the use of engineered, template-dependent polymerases more tolerant to LNA nucleotides.

## 2 Results and discussion

### 2.1 Design and synthesis of blocked DNA and LNA nucleotides

**FIGURE 1:**
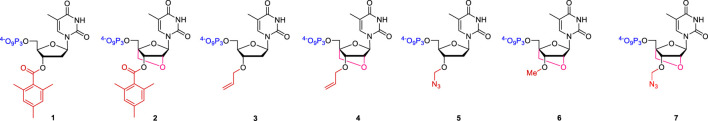
Structures of designed DNA and LNA nucleotides one to seven bearing 3′-*O*-blocking groups.

Benzoyl-protected LNA nucleotides were rather well-tolerated by a number of DNA polymerases and displayed an important resistance against hydrolytic removal (Flamme et al., 2022b). Despite these favorable assets, some polymerases including Kf(*exo* ^−^), *Bst* or Therminator were capable of abstracting the benzoyl masking group by their moonlighting esterase activity leading to multiple incorporation events. Substitution of the aromatic moiety of benzoates with methyl groups not only decreases the rate of hydrolysis under mild acidic conditions compared to the unsubstituted parent compound but also to a change from an A-2 (Watson) mechanism involving a water molecule in the transition state to an A-1 (Ingold) mechanism that proceeds *via* the formation of an acylium ion (Chmiel and Long, 1956; Shi et al., 2015; Pengthong et al., 2023). Based on this rationale, we deemed that the esterase activity of polymerases might be reduced by replacing a benzoyl- with a mesitoyl units on incoming nucleotides **1** and **2** (Figure 1).

Etherases catalyzing the hydrolysis of C-O bonds are quite rare in nature and essentially hydrolyze aryl ether bonds in lignin (Picart et al., 2015), uncommon vinyl ethers (Parsons et al., 2003), or lactyl ethers of MurNAc and related derivatives. This scarcity of naturally existing enzymes capable of hydrolyzing ether linkages is mainly due to the thermodynamic stability of C-O bonds (Jaeger et al., 2005). This feature has already been exploited in nucleic acid chemistry to develop blocked nucleotides for sequencing purposes (Ruparel et al., 2005; Ju et al., 2006; Wu et al., 2007; Guo et al., 2008; Keller et al., 2009; Knapp et al., 2011; Palla et al., 2014; Choi et al., 2022). Based on these considerations, we explored the possibility of using LNA nucleotides equipped with 3′-*O*-allyl (nucleotide **4**), 3′-*O*-methyl (nucleotide **6**), and 3′-*O*-azidomethyl (nucleotide **7**) protecting groups in controlled enzymatic XNA synthesis. In addition, docking experiments performed with nucleotide **7** and the TdT polymerase suggested that the modified nucleotide is rather well tolerated within the active site of the enzyme (Supplementary Figure S1).

In order to establish adequate control reactions, we also synthesized the known 3′-*O*-allyl- and 3′-*O*-azidomethyl-dTTP protected analogs (nucleotides **3** (Wu et al., 2007) and **5** (Guo et al., 2008), respectively).

**FIGURE 2:**
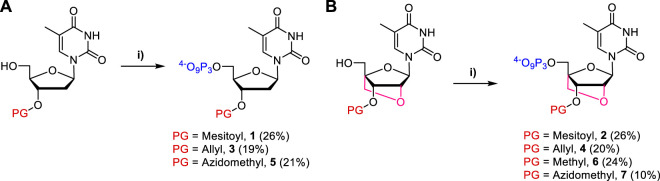
Synthesis of **(A)** DNA and **(B)** LNA nucleoside triphosphates one to seven bearing 3′-*O*-blocking groups. Reagents and conditions: 1) 2-chloro-1,3,2-benzodioxaphosphorin-4-one, pyridine, dioxane, rt, 1h; 2) (*n*Bu_3_NH)_2_H_2_P_2_O_7_, DMF, *n*Bu_3_N, rt, 1h; 3) I_2_, pyridine, H_2_O, rt, 30 min.

Based on this design, we first synthesized 3′-*O*-protected DNA and LNA nucleoside analogs starting either from 5′-*O*-DMTr- (for nucleotides **1**-**4** and **6**) or 5′-*O*-TBDMS-protected starting nucleosides (for nucleotides **5** and **7**) using protocols as described in detail in the supporting information and the literature (Obika et al., 1998; Singh et al., 1998; Christensen et al., 2001). After installation of the 3′-*O*-masking groups, the trityl and silyl protecting groups were removed under mild conditions. Finally, nucleoside triphosphates **1–7** were obtained in moderate yields (10%–26%) by application of the one-pot-three-steps protocol developed by Ludwig and Eckstein (Ludwig and Eckstein, 1989) (Figure 2).

### 2.2 Template independent synthesis

With blocked nucleotides **1–7** at hand, we sought to explore the possibility of constructing modified and natural oligonucleotides using controlled enzymatic synthesis. In this context, template-independent DNA polymerases such as the terminal deoxynucleotidyl transferase (TdT) (Sarac and Hollenstein, 2019; Ashley et al., 2023) are often considered as prime candidates for *de novo* synthesis of single-stranded DNA oligonucleotides (Lee et al., 2019; Jung et al., 2022; Lu et al., 2022; Wang et al., 2022). While the TdT polymerase is rather tolerant to a broad array of structurally modified nucleotides, it catalyzes the incorporation of single LNA nucleotides which then act as chain terminators even in the absence of 3′-*O*-blocking groups (Kuwahara et al., 2009; Kasahara et al., 2010; Flamme et al., 2021). Nonetheless, extension reactions with the TdT and 3′-*O*-blocked LNA nucleotides allow to rapidly gauge the substrate tolerance of a DNA polymerase for such modified analogs.

**FIGURE 3:**
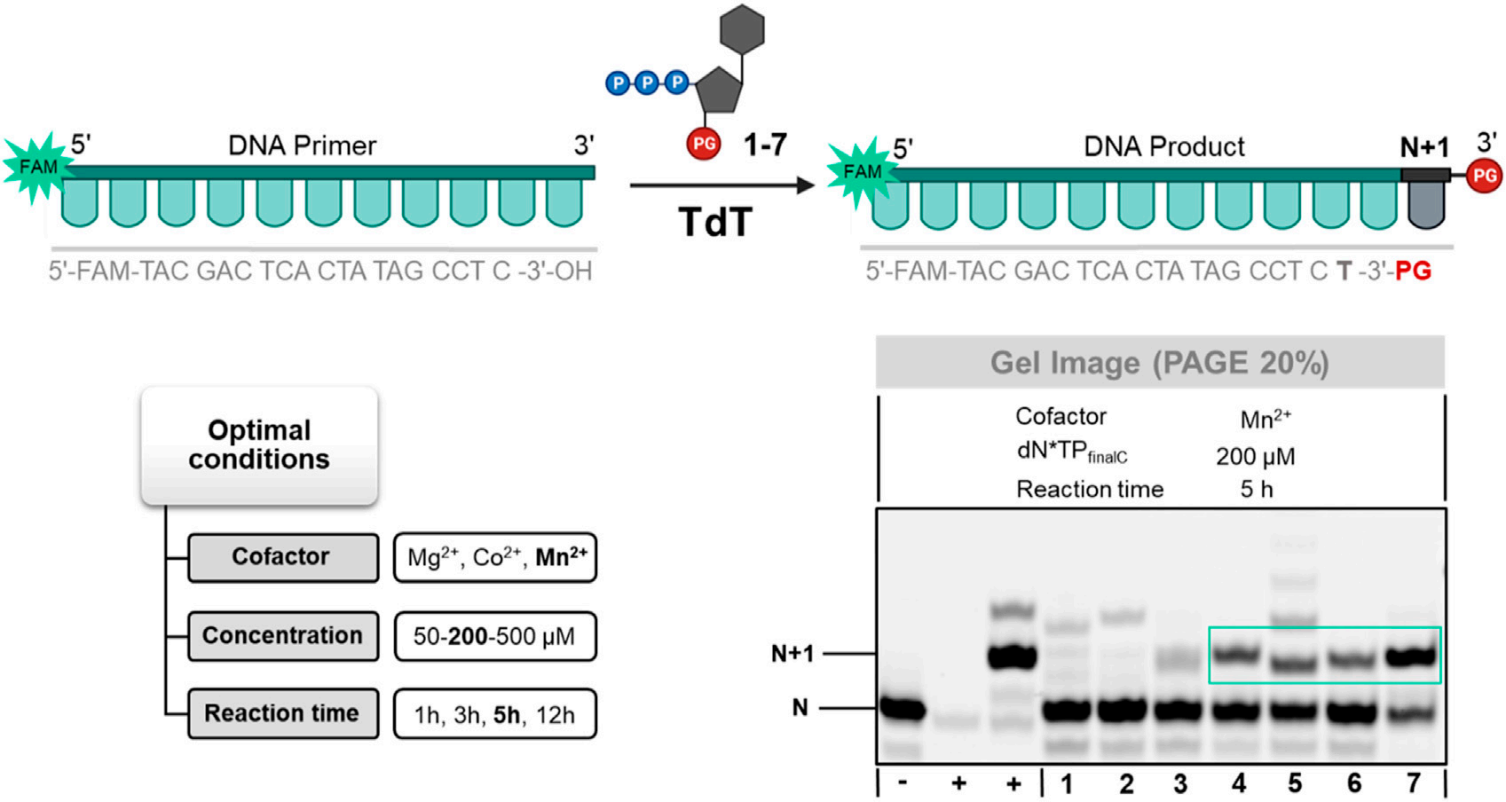
Gel image (PAGE 20%) of TdT-mediated tailing reactions with 3′-*O*-protected dN*TPs **1–7**. First from the left (**+**)—positive control using dTTP, second from the left (**+**)—positive control using 3′-OH-LNA-dN*TP, (**−**)—negative control in the absence of TdT.

Therefore, we first evaluated whether nucleotides **1–7** could act as substrates for the TdT. To do so, we incubated the modified nucleotides together with TdT, reaction buffer, various cofactors (Co^2+^, Mn^2+^, or Mg^2+^), and a 19 nucleotide long, 5′-FAM-labelled DNA primer for various reaction times (Figure 3; Supplementary Figure S2). Azidomethyl-protected nucleotide **7** displayed the best substrate tolerance by the TdT of all investigated nucleotides since conversion to the expected N+1 product could be achieved in near quantitative yields after 12 h of reaction with Mn^2+^ as cofactor. In addition to nucleotide **7**, 3′-*O*-allyl-blocked LNA analog **4** was also recognized as a substrate by the TdT albeit with lower efficiency (∼50% yield of conversion to N+1 product). Surprisingly, the corresponding DNA counterparts **3** and **5** were not well recognized by the TdT and significant amounts of further extended products could be detected by gel electrophoresis analysis. Analog **6** equipped with a 3′-*O*-methyl group was incorporated into DNA by the TdT with moderate efficiency (∼30% of conversion), while nucleotides **1** and **2** were not recognized as substrates. It is noteworthy mentioning that of all the conditions tested, the highest incorporation efficiencies were obtained, irrespective of the nature of the modified nucleotide, when Mn^2+^ was used as cofactor along with 200 µM triphosphate concentration and 5 h or 12 h of reaction time (Figure 3; Supplementary Figure S2). This is in contrast with 3′-*O*-benzoyl and 3′-*O*-pivaloyl-protected LNA-TTPs which displayed a marked preference for Co^2+^ over Mn^2+^ (Flamme et al., 2022b). The rather low yields observed with nucleotides **1**, **2**, **3**, and **5** might also be partially ascribed to the sequence bias of the TdT polymerase since 3′-terminal cytosine nucleotides on initiators are known to negatively impact the processivity of this enzyme (Schaudy et al., 2021).

We then subjected the reaction products obtained with nucleotides **4**, **6**, and **7** and the TdT to an LC-MS analysis (see Supplementary Information for experimental details). When methylated LNA-TTP **6** was engaged in the reaction mixture, the expected N+1 product could be detected by this analysis (*m*/*z* calcd.: 6588,1676; observed: 6,588,1960; see Supplementary Table S1). On the other hand, the 3′-allyl and 3′-azidomethyl moieties of the detected N+1 products obtained with nucleotides **4** and **7** are clearly absent. The main reaction products detected in these reactions was the N+1 product with 3′-OH moiety (*m*/*z* calcd.: 6574,1519; observed: 6,574,1834; see Supplementary Table S1). Collectively, these results suggest the possibility that under longer reaction times and in the presence of Mn^2+^ cofactor, ether protecting groups can be removed either as a consequence of the experimental conditions or through the effect of the TdT polymerase.

Poly(U) polymerases (PUPs) are another class of template-independent polymerase that catalyze the addition of rUMP residues at the 3′-termini of ssRNA in a mechanism reminiscent of that of the TdT (Kwak and Wickens, 2007; Munoz-Tello et al., 2012). PUPs have been employed for the terminal labelling of RNA oligonucleotides and shown a relative tolerance for sugar- and base-modified nucleotides (Winz et al., 2012; George et al., 2020; Vo et al., 2021; Gupta et al., 2022). Surprisingly, PUPs have not been considered for *de novo* synthesis of RNA or XNA oligonucleotides despite these favorable assets. We thus evaluated the possibility of using PUPs to incorporate blocked and unmodified LNA-TTP nucleotides into RNA given the structural preference of locked nucleic acids for an A-type conformation (Eichert et al., 2010; Campbell and Wengel, 2011).

**FIGURE 4:**
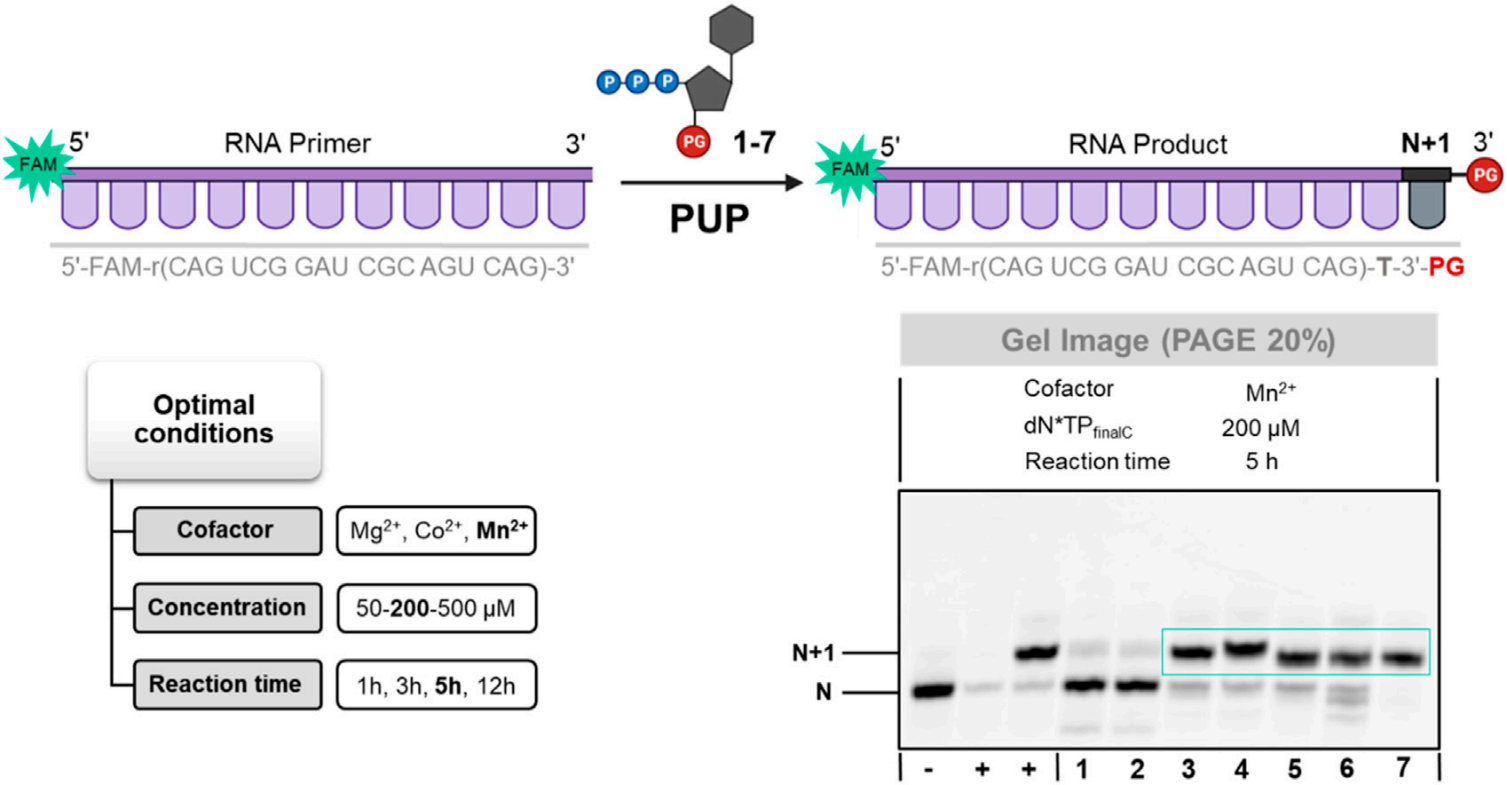
Gel image (PAGE 20%) of PUP-mediated tailing reactions with 3′-*O*-protected dN*TPs **1–7**. First from the left (**+**)—positive control using rUTP, second from the left (**+**)—positive control using 3′-OH-LNA-dN*TP, (**−**)—negative control in the absence of PUP.

To do so, we incubated an 18 nucleotide long, 5′-FAM-labelled RNA primer with LNA-TTP and nucleotides **1–7** with commercially available PUP under various experimental conditions including different cofactors, reaction times, and nucleotide concentrations (Figure 4; Supplementary Figure S3). With the exception of nucleotides **1** and **2** equipped with 3′-*O*-mesitoyl groups, the RNA polymerase PUP produced extended RNA primers with high efficiency (80%–95% yields of conversion to N+1 product) regardless of the nature of the nucleotide and the presence of blocking groups (see Figure 4). While PUP incorporated a single, unblocked LNA-TTP with a similar efficiency as TdT on DNA primers (Kuwahara et al., 2009; Flamme et al., 2021), this RNA polymerase appears to be much more tolerant to the presence of 3′-*O*-blocking groups than TdT. Indeed, DNA and LNA nucleotides equipped with 3′-*O*-methyl-, 3′-*O*-allyl-, and 3′-*O*-azidomethyl- protecting groups were equally well tolerated by PUP and successfully incorporated into RNA. Surprisingly, when the reaction product of 3′-OH-LNA-TTP was fed with UTP or increased concentrations of LNA-TTP no additional incorporation events could be observed (data not shown). Hence, LNA acts as a chain terminator in TdT-as well as in PUP-catalyzed reactions even in the absence of blocking groups.

In addition, we have analyzed the reaction products by LCMS to evaluate the nature of the products obtained by PUP-mediated catalysis. To do so, we subjected the reaction products obtained with nucleotides **3–7** and the PUP to an LCMS analysis (see Supplementary Information for experimental details). Unlike what has been observed with the TdT, the expected N+1 products still equipped with their respective protecting groups formed with nucleotides **3**–**6** (see Supplementary Table S2). On the other hand, the product obtained with nucleotide **7** corresponds to the primer extended by a single LNA-T nucleotide without any masking group at the 3′-end (*m*/*z* calcd: 6636,9811; observed: 6637,0165; see Supplementary Table S2). Clearly, reactions catalyzed by the PUP lead to the expected products with little or no removal of the masking groups.

### 2.3 Template dependent synthesis

**FIGURE 5:**
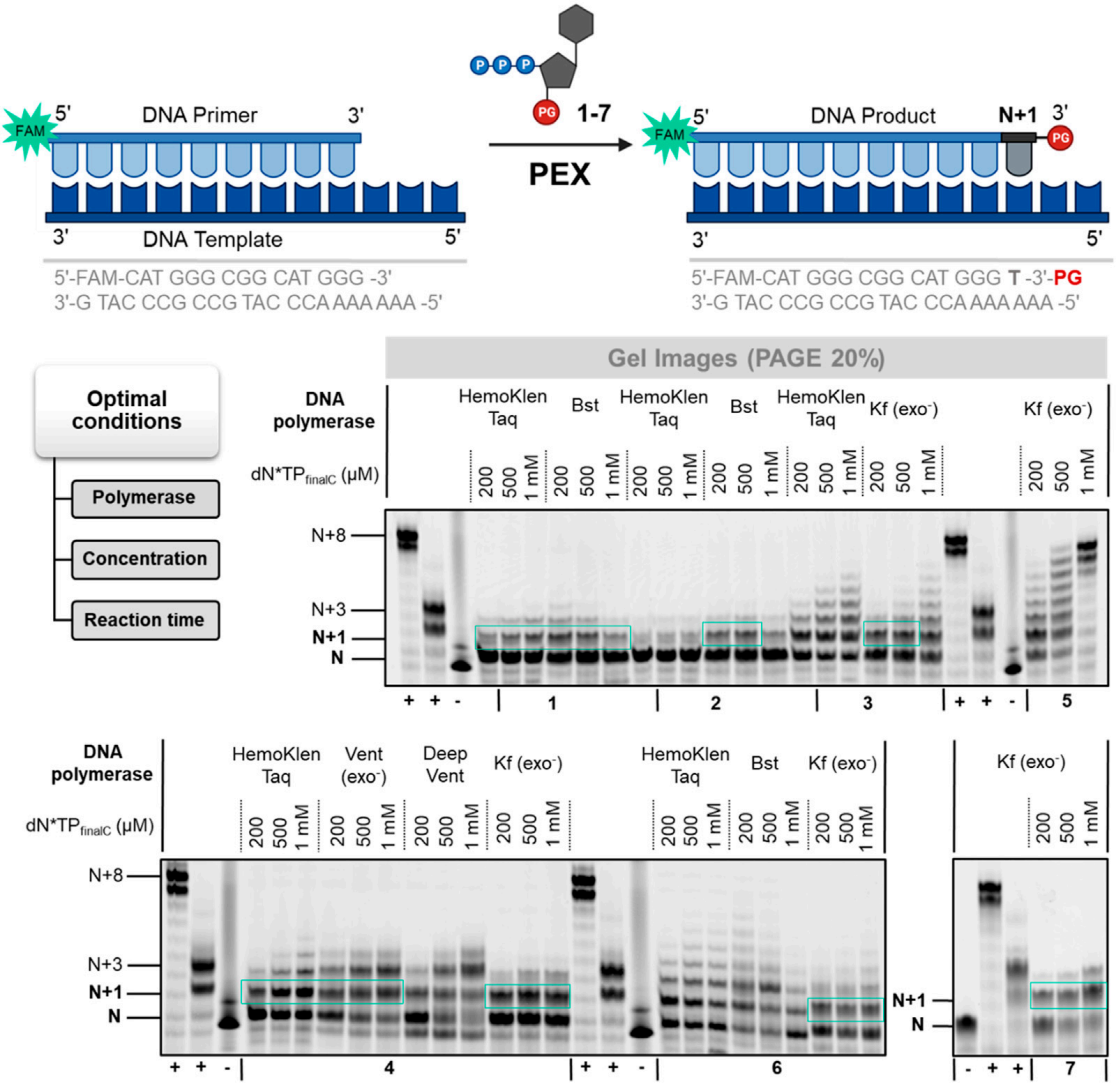
Gel image (PAGE 20%) of PEX reactions with 3′-*O*-protected dN*TPs **1–7**. First from the left (**+**)—positive control using dTTP, second from the left (**+**)—positive control using 3′-OH-LNA-dN*TP, (**−**)—negative control in the absence of polymerase.

While most efforts to improve the efficiency of *de novo* DNA synthesis are centered around TdT-mediated, template independent oligonucleotide production, template-dependent approaches are also emerging (Hoff et al., 2020; Hoose et al., 2023; Van Giesen et al., 2023). A main advantage of template-dependent synthesis is the plethora of polymerases that have been engineered to display very lax substrate requirements and which might be capable of incorporating blocked nucleotides. On the other hand, template-dependent synthesis leads to the formation of dsDNA *rather* than ssDNA products but this can be circumvented by immobilizing products on solid-support or to an extent by using universal templates (Hoff et al., 2020; Flamme et al., 2022b). Consequently, we set out to evaluate whether nucleotides **1–7** are compatible with enzymatic synthesis with template-dependent polymerases. To do so, we performed primer extension (PEX) reactions using a 15-mer, 5′-FAM-labelled primer and a 22-nucleotide long template equipped with a terminal poly (dA) stretch (Figure 5). We then evaluated the capacity of a small subset of polymerases (spanning over three families (A, B, and Y): Hemo KlenTaq, *Bst*, Vent (*exo* ^−^), *Sulfolobus* DNA polymerase IV, (Dpo4), Deep Vent, and Kf (*exo* ^−^)) at accepting nucleotides **1**–**7** as substrates and extending the primer by one nucleotide (Figure 5; Supplementary Figures S4A–C).

This analysis revealed that nucleotides **4** 3′-*O*-allyl-LNA-TTP, **6** 3′-*O*-methyl-LNA-TTP and **7** 3′-*O*-azidomethyl-LNA-TTP performed best of all evaluated analogs with over 50% of conversion of the primer to the expected N+1 product under optimized conditions. However, when higher dN*TP concentrations were employed, N+2 product formation was observed suggesting partial removal of the blocking group (Supplementary Figures S4A–C). An increase in dN*TP concentration was also accompanied by faster running bands which stem from hydrolytic degradation of the primer as observed with other modified nucleotides (Vastmans et al., 2001; Kuwahara et al., 2008; Flamme et al., 2022b). On the other hand, increasing the reaction time to 12 h led to a near completion of the primer and exclusive formation of the N+1 product (Supplementary Figure S4D). Nucleotides **3** and **4** equipped with 3′-*O*-allyl groups were tolerated by polymerases such as Kf (*exo* ^−^) but led to lower conversion yields (30%–40% of N+1 product formation). Interestingly, nucleotides **1** and **2** equipped with bulky ester groups were incorporated to a certain extent by the Taq and *Bst* polymerases under PEX reaction conditions unlike what had been observed with both the TdT and the PUP polymerases. Even though yields remained modest (∼20%), these incorporation events highlight the difference in substrate tolerance at the level of position 3′ of the deoxyribose sugar between template-dependent and template-independent polymerases. Lastly, reactions carried out with DNA nucleotide **5** (3′-*O*-azidomethyl-dTTP) led to the formation of a product distribution unlike those performed with the corresponding LNA analog. Overall, incorporation efficiencies in PEX reactions were comparable to those observed for related nucleotides blocked with ester moieties (Flamme et al., 2022b) and nucleotide **7** appeared to be the most promising candidate.

As for reactions catalyzed by template-independent polymerases, we subjected the resulting PEX reaction products to a thorough LCMS analysis to i) verify whether expected products were formed and ii) shed light into the product distribution observed with nucleotide **5** as well as the nature of the N+2 products. Analysis of the reaction products of nucleotides **3** and **4** obtained with Kf (*exo* ^−^) clearly highlights formation of the expected N+1 *but* without the 3′-*O*-allyl groups in both cases (Supplementary Table S3). While the removal of an allyl ether groups was not expected, these results are similar to those obtained with the TdT polymerase with these blocked nucleotides. Unfortunately, no product other than phosphorylated template could be observed in the reaction mixtures with nucleotides **1**, **2**, and **6**. Intrigued by these results, we tried to rationalize the loss of protecting groups observed by gel electrophoresis and LCMS analysis.

Concerning the azidomethyl protecting group, we believed this to arise due to the presence of the reducing agent dithiothreitol (DTT) both in the reaction and storage buffers of polymerases. Reducing agents convert azides to amines via a Staudinger reaction and the resulting aminomethyl moiety is then prone to hydrolysis as reported for blocked DNA nucleotides (Guo et al., 2008). In order to suppress this potential reduction event, we performed a PEX reaction with nucleotide **7** and Kf (*exo* ^−^) purchased without DTT in the storage buffer and in a reaction mixture devoid of the reducing agent (Supplementary Figure S4E). However, gel electrophoresis of the reaction product obtained after 12 h also showed the formation of the N+2 product. Addition of dTTP to the reaction mixture led to the formation of a product distribution thus suggesting partial removal of the protecting group. Lastly, treatment of the reaction mixture with potassium carbonate (1M, 3 h, RT) followed by incubation with canonical dTTP led to the same outcome. Overall, LCMS analysis combined with additional PEX reactions revealed that ether-blocked nucleotides were incorporated into DNA by polymerases but that the blocking groups were abstracted during the reactions.

In order to improve the yield of N+1 product formation, we considered generating a mutant of the KOD polymerase that would tolerate LNA nucleotides additionally modified at position 3’ of the sugar moiety. We thus considered engineering a KOD polymerase variant that contained one point mutation at the level of the exonuclease domain (P179S) and one in the thumb section (L650R). Point mutations were introduced at these sites because these have been recognized as facilitating the incorporation of sugar-modified nucleotides (Bergen et al., 2013; Larsen et al., 2016; Hoshino et al., 2020; Hajjar et al., 2022) and KOD was chosen given its tolerance for LNA nucleotides (Veedu et al., 2010). With this KOD mutant HP1.C2 at hand (courtesy from Roche), we carried out PEX reactions with blocked nucleotides **1**–**7** as well as unblocked LNA-TTP using similar conditions as described above.

First, we carried out PEX reactions with LNA-TTP and canonical dTTP to evaluate the proficiency of KOD mutant HP1.C2. Gel electrophoretic analysis revealed that the polymerase was capable of fully extending the primer with dTTP resulting in an N+8 product (which corresponds to full length product with an additional, untemplated addition). On the other hand, LNA-TTP was accepted as a substrate however only three nucleotides were incorporated into the primer (Supplementary Figure S4C). Similar efficiencies have been observed with other DNA polymerases (Flamme et al., 2021). We then extended our study to blocked nucleotides and gel electrophoretic analysis of the reaction products revealed that nucleotides **1**, **2**, **3**, and **6** were not tolerated at all by this polymerase since no or very little (<10% conversion of the primer) extended product could be observed (Supplementary Figures S4A, C). On the other hand, LNA nucleotide **4** was readily incorporated into DNA by the mutant polymerase but a lower running band suggested that undesired N+2 product formed while a faster running band suggested some partial hydrolytic degradation of the primer. Similarly, PEX reaction with DNA nucleotide **5** was readily incorporated and led to a distribution of N+1 and N+2 but no hydrolytic degradation of the primer. As noticed for other polymerases, nucleotide **7** acted as an excellent substrate for KOD mutant HP1.C2, since primer was fully converted (Supplementary Figure S4C). However, the main product stemming from this reaction was that corresponding to a double incorporation event (N+2) suggesting an abstraction of the protecting group during the reaction.

## 3 Discussion

Chemical synthesis of XNAs is particularly efficient for the development of potent therapeutic oligonucleotides (mainly antisense and siRNA) (McKenzie et al., 2021) but is more challenging for longer (>50 nucleotide-long) sequences (Taylor et al., 2016). On the other hand, polymerase-mediated synthesis grants access to very long sequences (Hoshino et al., 2020) but control of the localization of the modified nucleotides within the sequence is limited. The combination of both methods appears to be a potential strategy for the preparation of long oligonucleotides with modifications present at user defined positions. However, to reach these aims polymerases need to circumvent multiple hurdles in XNA *de novo* synthesis. Indeed, polymerases need to cope with modifications present at both the level of the sugar and the 3′-position. In addition, the masking group needs to be stable for longer time storage but concomitantly should be removable under mild conditions that would not affect the integrity of DNA and XNA oligonucleotides. Lastly, both incorporation of the blocked nucleotides and removal of the masking groups need to be high yielding and fast to be considered for the synthesis of longer oligonucleotides. In order to unravel such a potential protecting group candidate, we have focused on the *de novo* synthesis of LNA-containing oligonucleotides due to the relevance of this type of chemical modification in the context of therapeutic oligonucleotides (Campbell and Wengel, 2011; Hagedorn et al., 2018). In addition, LNA-TTP appears to be a more difficult substrate for polymerases since the presence of such a modification often induces rather high error-rates (Pinheiro et al., 2012; Hoshino et al., 2020).

So far, we have synthesized LNA nucleoside triphosphates equipped with a variety of 3′-*O*-blocking groups including phosphate, esters, and ethers. Nucleotides equipped with 3′-*O*-phosphate units suffer from poor polymerase acceptance due to the increased negative charge and relative bulkiness of the modification but also from rapid removal by the inherent phosphatase activity of polymerases (Flamme et al., 2022a). Ester protecting groups are better tolerated by polymerases but a fine balance between removal by the esterase capacity of polymerases and efficient incorporation needs to be evaluated in a case to case manner (Flamme et al., 2022b). Here, we have extended this approach to ether protecting groups and we have found that these nucleotides could be incorporated into DNA by template-independent and template-dependent reactions using DNA polymerases. However, incorporation efficiencies for N+1 product formation rarely exceed 60%–70% which is clearly unpractical for *de novo* XNA synthesis. In addition, removal of most of the 3′-*O*-ether protecting groups was observed and clearly results from an abstraction event during enzymatic reactions. The reasons for these unexpected ablations of the protecting groups remain unknown and might include reduction by DTT or other reagents, combined presence of divalent metal cations and long reaction times, or an etherase activity of polymerases. Interestingly, the RNA polymerase PUP incorporates only single LNA nucleotides which then act as chain terminators but unlike the TdT which displays a similar behaviour, the PUP is much more tolerant to the presence of 3′-modifications. Understanding the reason why LNA nucleotides act as chain terminators for reactions catalyzed by the TdT and the PUP polymerases would allow to engineer mutant enzymes that would be ideal candidates for *de novo* LNA and potentially XNA synthesis.

## 4 Conclusion

Here, we report the synthesis of various DNA and LNA nucleotides blocked with 3′-*O*-ether and more robust 3′-*O*-ester protecting groups, and their further biochemical evaluation in enzymatic reactions. We have shown that nucleotides equipped with ether linkages were tolerated by DNA and RNA polymerases while the ester moieties precluded incorporation into oligonucleotides presumably due to the increased bulkiness of the blocking group. We have also shown that the PUP polymerase readily tolerates 3′-*O*-masked LNA nucleotides as substrates and thus represents a valid alternative polymerase to be considered for *de novo* synthesis of XNA oligonucleotides. Similarly, we have evaluated the possibility of using an engineered DNA polymerase to increase product formation. Surprisingly, LCMS and gel electrophoresis analysis revealed that most ether linkages were abstracted during the enzymatic reactions. Hence, future directions for improving XNA *de novo* synthesis will include the evaluation of other protecting groups, engineered versions of the TdT and PUP polymerases, and potentially considering polymerases with the capacity to catalyze the formation of other linkages such as phosphoramidate bonds (Aggarwal et al., 2022).

## Data Availability

The original contributions presented in the study are included in the article/Supplementary Material, further inquiries can be directed to the corresponding author.
