# Supplementary material for: Towards the controlled enzymatic synthesis of LNA containing oligonucleotides
Source: Front Chem. 2023 Apr 27;11:1161462. doi: 10.3389/fchem.2023.1161462 (PMC10172484; doi:10.3389/fchem.2023.1161462)

Supplementary Material

**Towards the controlled enzymatic synthesis of LNA containing oligonucleotides**

**Nazarii Sabat,^a^ Dace Katkevica,^b^ Karlis Pajuste,^b^ Marie Flamme,^a^ Andreas Stämpfli,^c^ Martins Katkevics,^b^ Steven Hanlon,^d^ Serena Bisagni,^d^ Kurt Püntener,^d^ Filippo Sladojevich,^c^ and Marcel Hollenstein^a^***

^a^ Institut Pasteur, Université de Paris Cité, CNRS UMR3523, Department of Structural Biology and Chemistry, Laboratory for Bioorganic Chemistry of Nucleic Acids, 28, rue du Docteur Roux, 75724 Paris Cedex 15, France

^b^ Latvian Institute of Organic Synthesis, Aizkraukles 21, Riga, LV-1006, Latvia

^c^ Pharma Research and Early Development, Roche Innovation Center Basel, F. Hoffmann-La Roche Ltd, Grenzacherstrasse 124, 4070, Basel, Switzerland

^d^ Pharmaceutical Division, Synthetic Molecules Technical Development, Process Development & Catalysis, F. Hoffmann-La Roche Ltd, 4070 Basel, Switzerland

***** Corresponding Author: marcel.hollenstein@pasteur.fr

**Contents**

[1. Materials and methods 2](#_Toc126330782)

[2. Experimental section. 3](#_Toc126330783)

[2a. Synthesis of starting nucleosides. 3](#_Toc126330784)

[2b. Nucleoside triphosphorylation. General procedure. 11](#_Toc126330785)

[3. Copies of NMR spectra 15](#_Toc126330786)

[4. Enzymatic reactions. General protocols. 28](#_Toc126330787)

[4a. TdT reactions 28](#_Toc126330788)

[4b. PUP reactions 29](#_Toc126330789)

[4c. PEX reactions 30](#_Toc126330790)

[5. LCMS analyses of enzymatic reactions 35](#_Toc126330791)

# Materials and methods

All reactions were performed under argon in flame-dried glassware. Anhydrous solvents for reactions were purchased from Sigma Aldrich. Flash chromatography was performed using silica gel (230–400 mesh) from Sigma Aldrich or on the Reveleris Prep system from Büchi. Thin layer chromatography was carried out on pre-coated glass-backed plates of silica gel (0.25 mm, UV_254_) from Macherey-Nagel. All chemicals and solvents used were purchased from Sigma-Aldrich and Alfa Aesar. NMR spectra were recorded on a Bruker Avance 500 spectrometer (500.1 MHz for ^1^H, 125.8 MHz for ^13^C, and 202.5 MHz for ^31^P) or Varian Mercury spectrometer (400 MHz for ^1^H) and all spectra were referenced to the signals of the corresponding solvent. Chemical shifts are given in ppm (δ scale) and coupling constants (*J*) in Hz. Assignation of the NMR signals was performed by using a combination of ^1^H/^1^H-COSY, ^13^C-DEPT-135, and ^13^C/^1^H-HMBC experiments. High-resolution electrospray ionization (ESI) mass spectra (MS, m/z) were recorded on a Waters Q-Tof Micro MS in the positive-ion electrospray ionization (ESI+) mode. HPLC purification was performed using an Äkta™ pure system (GE Healthcare) equipped with Thermo Scientific™ DNAPac™ PA100 preparative ion exchange column (13 µm, 250 x 22.0 mm). Unmodified DNA oligonucleotides were purchased from Microsynth or IDT companies. All the DNA polymerases (TdT, Phusion, Hemo KlenTaq, Taq, *Bst*, Q5, Therminator, Vent (*exo*^-^), Dpo4, Deep Vent and Kf *exo*^-^) and RNA PolyU polymerase were purchased from New England Biolabs as well as the natural dNTPs. Acrylamide/bisacrylamide (29:1, 40%) was obtained from Fisher Scientific. Visualization of PAGE gels was performed by fluorescence imaging using Typhoon Trio phosphorimager with the ImageQuant software (both from GE Healthcare).

# **Experimental section**.

## 2a. Synthesis of starting nucleosides.

**(*2R,3S,5R*)-2-((Bis(4-methoxyphenyl)(phenyl)methoxy)methyl)-5-(5-methyl-2,4-dioxo-3,4-dihydropyrimidin-1(*2H*)-yl)tetrahydrofuran-3-yl 2,4,6-trimethylbenzoate** (**S1).**

To a solution of **DMTr DNA-T** (800 g, 1.46 mmol) in DCM (15 mL) dry pyridine (1.17 mL, 14.6 mmol) was added and mixture was cooled to 0 ^o^C (ice bath). Solution of 2,4,6-trimethylbenzoyl chloride (346 mg 1.89 mmol) in DCM (5 mL) was added dropwise. The reaction mixture was stirred at 0 ^o^C for 3 h. DCM (20 mL) was added and mixture was extracted with sat. NaHCO_3_ solution (20 mL). Organic phase was separated, dried over Na_2_SO_4_, and concentrated. The residue was purified by silica gel chromatography (EtOAc/hexane + 0.5% NEt_3_) to afford title compound **S1** as white foam (756 mg, 75%). ^1^H NMR (400 MHz, CDCl_3_) δ 7.61 (q, *J* = 1.2 Hz, 1H), 7.41 – 7.36 (m, 2H), 7.33 – 7.21 (m, 7H), 6.87 – 6.81 (m, 6H), 6.35 (dd, *J* = 7.4, 5.9 Hz, 1H), 4.56 (dt, *J* = 6.3, 3.2 Hz, 1H), 4.03 (q, *J* = 3.1 Hz, 1H), 3.79 (s, 6H), 3.48 (dd, *J* = 10.6, 3.2 Hz, 1H), 3.36 (dd, *J* = 10.6, 3.1 Hz, 1H), 2.41 (ddd, *J* = 13.6, 5.9, 3.2 Hz, 1H), 2.35 – 2.24 (m, 1H), 2.37 (s, 6H), 2.35 – 2.24 (m, 1H), 2.27 (s, 3H), 1.45 (d, *J* = 1.2 Hz, 3H).

**(*1S,3R,4R,7S*)-1-(Hydroxymethyl)-3-(5-methyl-2,4-dioxo-3,4-dihydropyrimidin-1(*2H*)-yl)-2,5-dioxabicyclo[2.2.1]heptan-7-yl 2,4,6-trimethylbenzoate (S2)**

To a solution of **S1** (700 mg, 1.01 mmol) in DCM (30 mL) TFA (1.5 mL) and triethylsilane (700 mg, 6.01 mmol) was added. The reaction mixture was stirred at room temperature for 30 min and then evaporated. The residue was purified by silica gel chromatography (DCM/EtOAc) to afford the title compound **S2** as white foam (285 mg, 85%). ESI-MS (m/z): 389.5 [M+H]^+^ . ^1^H NMR (400 MHz, CDCl_3_) δ 8.92 (s, 1H), 7.75 (q, *J* = 1.3 Hz, 1H), 7.45 – 7.37 (m, 2H), 5.78 (d, *J* = 0.7 Hz, 1H), 5.42 (s, 1H), 4.80 (d, *J* = 0.7 Hz, 1H) 3.93 – 3.83 (m, 2H), 3.65 (d, *J* = 10.9 Hz, 1H), 3.43 (d, *J* = 10.9 Hz, 1H ), 2.27 (s, 3H), 2.13 (s, 6H), 1.61 (d, *J* = 1.3 Hz, 3H).

**1-((*2R,4S,5R*)-4-(Allyloxy)-5-((bis(4-methoxyphenyl)(phenyl)methoxy)methyl)tetrahydrofuran-2-yl)-5-methylpyrimidine-2,4(*1H,3H*)-dione** **(S3)**

To a suspension of 60% NaH (128 mg, 3.19 mmol) in THF (10 mL) **DMTr DNA-T** (700 mg, 1.28 mmol) was added at 0 ^o^C (ice bath ) and mixture was stirred for 30 min. Allyl bromide (280 μL, 3.24 mmol) was added and the reaction mixture was stirred at room temperature for 4 h. The reaction mixture was poured into sat. NaHCO_3_ (20 mL) and extracted with EtOAc (3 × 20 mL). EtOAc extracts were combined, dried over Na_2_SO_4_, and concentrated. The residue was purified by silica gel chromatography (EtOAc/hexane + 0.5% NEt_3_) to afford title compound **S3** as white foam (530 mg, 71%). ^1^H NMR (400 MHz, acetone-d_6_) δ 9.94 (br s, 1H), 7.60 (q, *J* = 1.3 Hz, 1H), 7.52 – 7.46 (m, 2H), 7.39 – 7.31 (m, 6H), 7.30 – 7.23 (m, 1H), 6.95 – 6.85 (m, 4H), 6.35 – 6.25 (m, 1H), 5.90 (ddt, *J* = 17.3, 10.6, 5.4 Hz, 1H), 5.26 (dq, *J* = 17.3, 1.7 Hz, 1H), 5.14 (ddt, *J* = 10.5, 2.0, 1.4 Hz, 1H), 4.41 – 4.33 (m, 1H), 4.13 (1td, *J* = 3.5, 2.7 Hz, 1H), 4.09 – 3.98 (m, 2H), 3.79 (s, 6H) 3.42 – 3.34 (m, 2H), 2.47 – 2.29 (m, 2H), 1.48 (d, *J* = 1.3 Hz, 3H).

**1-((*2R,4S,5R*)-4-(Allyloxy)-5-(hydroxymethyl)tetrahydrofuran-2-yl)-5-methylpyrimidine-2,4(*1H,3H*)-dione (S4)**

To a solution of **S3** (500 mg, 0.86 mmol) in DCM (15 mL) TFA (0.6 mL) and triethylsilane (298 mg, 257 mmol) was added. The reaction mixture was stirred at room temperature for 30 min and then evaporated. The residue was purified by silica gel chromatography (MeOH/EtOAc) to afford the title compound **S4** as white foam (203 mg, 83%). ESI-MS (m/z): 283.3 [M+H]^+^ . ^1^H NMR (400 MHz, DMSO-d_6_) δ 11.30 (s, 1H), 7.69 (q, *J* = 1.3 Hz, 1H), 6.12 (dd, *J* = 8.4, 5.8 H, 1H), 5.90 (ddt, *J* = 17.2, 10.4, 5.3 Hz, 1H), 5.28 (dq, *J* = 17.2, 1.8 Hz, 1H), 5.16 (dq, *J* = 10.4, 1.5 Hz, 1H), 5.09 (s, 1H), 4.12 – 4.06 (m, 1H), 4.01 – 3.97 (m. 2H), 3.94 – 3.90 (m, 1H), 3.64 – 3.51 (m, 2H), 2.22 (ddd, *J* = 13.8, 5.8, 2.3 Hz, 1H), 2.12 (ddd, *J* = 13.8, 8.4, 5.8 Hz, 1H), 1.77 (d, *J* = 1.3 Hz, 3H).

**1-((*2R,4S,5R*)-4-(Azidomethoxy)-5-(hydroxymethyl)tetrahydrofuran-2-yl)-5-methylpyrimidine-2,4(*1H,3H*)-dione (S5).**

To a solution of 5’-0-(tertbutyldimethylsilyl) thymidine **TBDMS DNA T** (1.50 g, 4.21 mmol) in DMSO (10 mL) AcOH (5 mL) and Ac_2_O (15 mL) at 25 °C were added. After stirring for 48 h, saturated NaHCO_3_ (100 mL) and EtOAc (80 mL) were added and the aqueous layer was extracted with EtOAc (3 × 80 mL). The EtOAc extracts were combined, washed with brine (300 mL), dried over Na_2_SO_4_ and evaporated. The residue was dissolved in EtOAc and passes through silica gel plug. Plug was washed with EtOAc and filtrate was evaporated. The residue was dissolved in DCM (20 mL), cyclohexene (1.5 mL) and SO_2_Cl_2_ (0.47 mL 1.91 mmol) was added at 0 °C. Reaction mixture was stirred at 0 °C for 3h and then evaporated. The residue was dried at vacuum (1 × 10^-2^ mbar) for 10 min and then was dissolved in DMF (15 mL). NaN_3_ (1.20 g 19.8 mmol) was added to the reaction mixture was stirred at room temperature for 3 h. DCM (50 mL) and water (50 mL) was added and extracted with DCM (3 × 50 mL). DCM extracts were combined, dried over Na_2_SO_4_ and evaporated. The residue was dissolved in THF (20 mL) and solution of TBAF (1M in THF, 4.50 mL, 4.50 mmol) was added, Reaction mixture was stirred at room temperature for 3h and then evaporated. EtOAc (30 mL) and water (30 mL) was added and extracted with EtOAc (3 × 30 mL). EtOAc extracts were combined, dried over Na_2_SO_4_ and evaporated. The residue was purified several times by column chromatography on silica gel (EtOAc/hexane and EtOAc/MeOH) to afford the title compound **S5** as white foam (320 mg, 24% yield in three steps). ESI-MS (m/z): 298.3 [M+H]^+^ . ^1^H NMR (400 MHz, CDCl_3_) δ 8.54 (s, 1H), 7.36 (q, *J* = 1.3 Hz, 1H), 6.11 (dd, *J* = 7.4, 6.5 Hz, 1H), 4.75 (d, *J* = 9.1 Hz, 1H), 4.69 (d, *J* = 9.1 Hz, 1H), 4.50 – 4.45 (m, 1H), 4.13 (q, *J* = 2.9 Hz, 1H), 3.97 (dd, *J* = 11.9, 2.7 Hz, 1H), 3.83 (dd, *J* = 11.9, 2.9 Hz, 1H), 2.55 (br. s, 1H), 2.47–2.40 (m, 2H), 1.92 (d, *J* = 1.3 Hz, 3H)..

**(*1R,3R,4R,7S*)-1-((Bis(4-methoxyphenyl)(phenyl)methoxy)methyl)-3-(5-methyl-2,4-dioxo-3,4-dihydropyrimidin-1(*2H*)-yl)-2,5-dioxabicyclo[2.2.1]heptan-7-yl 2,4,6-trimethylbenzoate (S6)**

To a solution of **DMTr DNA-T** (800 g, 1.39 mmol) in DCM (15 mL) dry pyridine (1.11 mL, 1.39 mmol) was added and mixture was cooled to 0 ^o^C (ice bath). Solution of 2,4,6-trimethylbenzoyl chloride (329 mg 1.81 mmol) in DCM (5 mL) was added dropwise. The reaction mixture was stirred at 0 ^o^C for 3 h. DCM (20 mL) was added and mixture was extracted with sat. NaHCO_3_ solution (20 mL). Organic phase was separated, dried over Na_2_SO_4_, and concentrated. The residue was purified by silica gel chromatography (EtOAc/hexane + 0.5% NEt_3_) to afford title compound **S6** as white foam (653 mg, 66%). ^1^H NMR (400 MHz, CDCl_3_) δ 8.92 (s, 1H), 7.75 (q, *J* = 1.3 Hz, 1H), 7.45 – 7.37 (m, 2H), 7.34 – 7.20 (m, 7H), 6.87 – 6.76 (m, 6H), 5.78 (d, *J* = 0.7 Hz, 1H), 5.42 (s, 1H), 4.80 (d, *J* = 0.7 Hz, 1H), 3.93 – 3.83 (m, 2H), 3.79 (s, 3H), 3.78 (s, 3H), 3.65 (d, *J* = 10.9 Hz, 1H), 3.43 (d, *J* = 10.9 Hz, 1H), 2.27 (s, 3H), 2.13 (s, 6H), 1.61 (d, *J* = 1.3 Hz, 3H).

**(1S,3R,4R,7S)-1-(Hydroxymethyl)-3-(5-methyl-2,4-dioxo-3,4-dihydropyrimidin-1(2H)-yl)-2,5-dioxabicyclo[2.2.1]heptan-7-yl 2,4,6-trimethylbenzoate (S7)**

To a solution of **S6** (612 mg, 0.85 mmol) in DCM (25 mL) TFA (1.5 mL) and triethylsilane (350 mg, 3.01 mmol) was added. The reaction mixture was stirred at room temperature for 30 min and then evaporated. The residue was purified by silica gel chromatography (DCM/EtOAc) to afford the title compound **S7** as white foam (285 mg, 85%). ESI-MS (m/z): 417.43 [M+H]^+^ . ^1^H NMR (400 MHz, CDCl_3_) δ 8.36 (s, 1H), 7.69 (q, *J* = 1.2 Hz, 1H), 6.89 (s, 2H), 5.74 (s, 1H), 5.08 (s, 1H), 4.75 (s, 1H), 4.05 (d, *J* = 13.3 Hz, 1H), 3.91 (s, 2H), 3.91 (d, *J* = 13.3 Hz, 1H), 2.32 (s, 6H), 2.30 (s, 3H), 1.99 (d, *J* = 1.2 Hz, 3H).

**1-((*1R,3R,4R,7S*)-7-(Allyloxy)-1-((bis(4-methoxyphenyl)(phenyl)methoxy)methyl)-2,5-dioxabicyclo[2.2.1]heptan-3-yl)-5-methylpyrimidine-2,4(*1H,3H*)-dione (S8)**

To a suspension of 60% NaH (127 mg, 4.55 mmol) in THF (10 mL) **DMTr LNA-T** (800 mg, 1.39 mmol) was added at 0^°^C (ice bath) and mixture was stirred for 30 min. Allyl bromide (168 μL, 1.95 mmol) was added and the reaction mixture was stirred at room temperature for 4 h. The reaction mixture was poured into sat. NaHCO_3_ (20 mL) and extracted with EtOAc (3 × 20 mL). EtOAc extracts were combined, dried over Na_2_SO_4_, and concentrated. The residue was purified by silica gel chromatography (EtOAc/hexane + 0.5% NEt_3_) to afford title compound **S8** as white foam (530 mg, 61%). ^1^H NMR (400 MHz, CDCl_3_) δ 8.37 (s, 1H), 7.76 (q, *J* = 1.3 Hz, 1H), 7.49 – 7.41 (m, 2H), 7.36 – 7.28 (m, 6H), 7.28 – 7.21 (m, 2H), 6.89 – 6.80 (m, 4H), 5.82 (ddt, *J* = 17.3, 10.5, 5.5 Hz, 1H), 5.64 (d, *J* = 0.7 Hz, 1H), 5.26 – 5.19 (m, 1H), 5.17 (dq, *J* = 10.5, 1.3 Hz, 1H), 4.55 (d, *J* = 0.7 H, 1H), 4.17 – 4.06 (m, 1H), 3.99 (ddt, *J* = 12.8, 5.5, 1.5 Hz, 1H), 3.87 (d, *J* = 7.8 Hz, 1H), 3.80 (s, 3H), 3.80 (s, 3H), 3.75 (d, *J* = 7.8 Hz, 1H), 3.56 (d, *J* = 11.0 Hz, 3.80 (s, 1H), 3.36 (d, *J* = 11.0 Hz, 1H), 1.69 d, *J* = 1.3 Hz, 3H).

**1-((*1S,3R,4R,7S*)-7-(Allyloxy)-1-(hydroxymethyl)-2,5-dioxabicyclo[2.2.1]heptan-3-yl)-5-methylpyrimidine-2,4(*1H,3H*)-dione (S9)**

To a solution of **S8** (500 mg, 0.82 mmol) in DCM (10 mL) TFA (0.40 mL) and triethylsilane (286 mg, 2.46 mmol) was added. The reaction mixture was stirred at room temperature for 30 min and then evaporated. The residue was purified by silica gel chromatography (MeOH/EtOAc) to afford the title compound **S9** (187 mg, 73%). ESI-MS (m/z): 311.2 [M+H]^+^ . ^1^H NMR (400 MHz, DMSO-d_6_) δ 11.36 (s, 1H), 7.60 (q, *J* = 1.2 Hz, 1H), 5.87 (ddt, *J* = 17.3, 10.5, 5.2 Hz, 1H), 5.46 (s, 1H), 5.31-5.27 (m, 2H), 5.13 (ddt, *J* = 10.5, 2.1, 1.4 Hz, 1H), 4.42 (s, 1H), 4.08 – 4.04 (m, 2H), 3.86 (s, 1H), 3.81 (d, *J* = 7.9 Hz, 1H), 3.77 (dd, *J* = 5.7, 3.3 Hz, 2H), 3.66 (d, *J* = 7.7 Hz, 1H), 1.78 (d, *J* = 1.2 Hz, 3H).

**1-((*1R,3R,4R,7S*)-1-((Bis(4-methoxyphenyl)(phenyl)methoxy)methyl)-7-methoxy-2,5-dioxabicyclo[2.2.1]heptan-3-yl)-5-methylpyrimidine-2,4(*1H,3H*)-dione (S10)**

To a suspension of 60% NaH (128 mg, 3.19 mmol) in THF (10 mL) **DMTr LNA-T** (800 mg, 1.46 mmol) was added at 0^°^C (ice bath) and mixture was stirred for 30 min. MeI (182 μL, 2.93 mmol) was added slowly and the reaction mixture was stirred at room temperature for 4h. The reaction mixture was poured into sat. NaHCO_3_ (20 mL) and extracted with EtOAc (3 × 20 mL EtOAc extracts were combined, dried over Na_2_SO_4_, and concentrated. The residue was purified by silica gel chromatography (EtOAc/hexane + 0.5% NEt_3_) to afford title compound **S10** as white foam (580 mg, 67%). ^1^H NMR (400 MHz, CDCl_3_) δ 8.25 (s, 1H), 7.74 (q, *J* = 1.2 Hz, 1H), 7.49 – 7.41 (m, 2H), 7.38 – 7.28 (m, 6H), 7.28 – 7.21 (m, 1H), 6.90 – 6.80 (m, 4H), 5.65 (d, *J* = 0.8 Hz, 1H), 4.57 (d, *J* = 0.8 Hz, 1H), 3.96 (s, 1H), 3.84 (d, *J* = 7.8 Hz, 1H) 3.80 (s, 6H), 3.75 (d, *J* = 7.8 Hz, 1H), 3.55 (d, *J* = 11.0 Hz, 1H), 3.40 (s, 3H), 3.37 (d, *J* = 11.0 Hz, 1H), 1.69 (d, *J* = 1.2 Hz, 3H).

**1-((*1S,3R,4R,7S*)-1-(Hydroxymethyl)-7-methoxy-2,5-dioxabicyclo[2.2.1]heptan-3-yl)-5-methylpyrimidine-2,4(*1H,3H*)-dione** **(S11)**

To a solution of **S10** (560 mg, 0.95 mmol) in DCM (25 mL) TFA (1.5 mL) and triethylsilane (331 mg, 2.85 mmol) was added. The reaction mixture was stirred at room temperature for 30 min and then evaporated. The residue was purified by silica gel chromatography (DCM/EtOAc) to afford the title compound **S11** as white foam (173 mg, 64%). ESI-MS (m/z): 285.3 [M+H]^+^. ^1^H NMR (400 MHz, DMSO-d_6_) δ 11.36 (s, 1H), 7.60 (q, *J* = 1.2 Hz, 1H), 5.46 (s, 1H), 5.28 (t, *J* = 5.3 Hz, 1H), 4.44 (s, 1H), 3.78 – 3.73 (m, 4H), 3.65 (d, *J* = 7.8 Hz, 1H), 3.31 (s, 3H), 1.79 (d, *J* = 1.2 Hz, 3H).

**1-((*1R,3R,4R,7S*)-1-(((tert-butyldimethylsilyl)oxy)methyl)-7-hydroxy-2,5-dioxabicyclo[2.2.1]heptan-3-yl)-5-methylpyrimidine-2,4(*1H,3H*)-dione (S12)**

**LNA T** (188 mg, 0.696 mmoles, 1 eq) was dissolved in dry DMF under inert atmosphere. Imidazole (115 mg, 0.765 mmoles, 1.1 eq) was added and the mixture was cooled to 0°C. TBDMSCl (115 mg, 0.765 mmoles, 1.1 eq) in dry DMF was added over 5 min. The mixture was allowed to warm to room temperature and was stirred for 12h. The crude mixture was concentrated under vacuo and purified by flash chromatography (DCM/MeOH 90:10) to give 140 mg of **S12** a white powder (51% yield). ^1^H NMR (500.02 MHz, MeOD) δ 7.63 (s, 1H), 5.52 (s, 1H), 4.26 (s, 1H), 4.04 (s, 1H), 3.99 (s, 2H), 3.72-3.94 (m, 2H), 1.87 (s, 3H), 0.96 (s, 9H), 0.15 (s, 6H). HRMS (ESI) for C_17_H_29_N_2_O_6_Si^+^ m/z calcd: 385.1789; found: 385.1780.

**1-((*1R,3R,4R,7S*)-1-(((tert-butyldimethylsilyl)oxy)methyl)-7-((methylthio)methoxy)-2,5-dioxabicyclo[2.2.1]heptan-3-yl)-5-methylpyrimidine-2,4(*1H,3H*)-dione (S13)**

To a stirred solution of **S12** (140 mg, 0.364 mmoles, 1 eq) in DMSO (0.7 mL), acetic acid (0.3 mL) and acetic anhydride (1 mL) were added. The reaction mixture was stirred at room temperature for 48h. A saturated NaHCO_3_ solution (20 mL) was added and the aqueous phase was extracted with ethyl acetate (3 x 20 mL). The combined organic layers were washed with a saturated solution of NaHCO_3_ and dried over MgSO_4_. After concentration, the crude was purified by flash chromatography column (EtOAc/hexane 7:3 to 1:1) to afford 122 mg of **S13** as white powder (75% yield). ^1^H NMR (500.02 MHz, CDCl_3_) δ 9.83 (bs, 1H), 7.53 (s, 1H), 5.62 (s, 1H), 4.66-4.67 (m, 2H), 4.59 (s, 1H), 4.10 (s, 1H), 4.10 (s, 1H), 2.10 (s, 3H), 2.00 (s, 1H), 1.90 (s, 3H), 1.20-1.23 (m, 1H), 0.92 (s, 9H), 0.10 (d, *J* = 5.50 Hz, 6H). ^13^C NMR (100.62 MHz, CDCl_3_) δ 167.0, 152.7, 137.0, 112.9, 91.3, 89.7, 80.0, 79.7, 79.5, 77.2, 76.2, 74.6, 63.0, 60.5, 21.0, 16.8, 16.6, 15.2, -2.7. HRMS (ESI) for C_19_H_33_N_2_O_6_SSi^+^ m/z calcd: 445.1823; found: 445.1820.

**1-((*1S,3R,4R,7S*)-7-(azidomethoxy)-1-(hydroxymethyl)-2,5-dioxabicyclo[2.2.1]heptan-3-yl)-5-methylpyrimidine-2,4(*1H,3H*)-dione (S14)**

To a stirred solution of **S13** (122 mg, 0.274 mmoles, 1 eq) in dry DCM (2 mL) under argon was added SO_2_Cl_2_ (28 µL, 0.336 mmoles, 1.3 eq). The reaction mixture was stirred at 0°C for 1h. The solvent was removed under reduced pressure and dissolved in dry DMF (2 mL). It was reacted with NaN_3_ (106 mg, 1.644 mmoles, 6 eq) at room temperature for 3h. The reaction mixture was dispersed in distilled water (20 mL) and extracted with DCM (3 x 20 mL). The organic layer was dried and concentrated under reduced pressure. The residue was dissolved in methanol (2 mL) and reacted with NH_4_F (64 mg, 1.726 mmoles, 6.3 eq) at room temperature for 24h. The reaction mixture was concentrated and partitionned between distilled water (20 mL) and DCM (3 x 20 mL). The organic layer was dried and concentrated under reduced pressure and the residue was purified by flash chromatography column (EtOAc/hexane 1:1 to 2:5) to afford 30 mg of **S14** as white powder (52% yield). ^1^H NMR (500.02 MHz, CDCl_3_) δ 8.52 (bs, 1H), 7.51 (s, 1H), 5.64 (s, 1H), 4.73-4.81 (m, 2H), 4.63 (s, 1H), 4.10 (s, 1H), 4.04-4.06 (m, 1H), 3.97-4.00 (m, 2H), 3.84-3.86 (m, 1H), 3.84-3.86 (m, 1H), 1.93 (s, 3H). ^13^C NMR (100.62 MHz, CDCl_3_) δ 166.1, 152.3, 137.1, 113.3, 90.8, 90.0, 84.7, 80.2, 78.2, 74.4, 60.0, 15.5. HRMS (ESI) for C_12_H_16_N_5_O_6_^+^ m/z calcd: 326.1095; found: 326.1105.

## 2b. Nucleoside triphosphorylation. General procedure.

Nucleosides **S2, S4, S5, S7, S9, S11, S14** (0.15 mmol, 1.0 equiv.) were dried under reduced pressure overnight prior to reaction, then dissolved in the mixture (1:2) of dry pyridine (0.25 ml) and dioxane (0.50 ml) under argon (Suppl. Figure 1). To this solution, 2-chloro-1,3,2-benzodioxaphosphorin-4-one (36.5 mg, 0.18 mmol, 1.2 equiv.) in dry dioxane (0.25 mL) was added and the reaction mixture was stirred for 1 hour at room temperature under argon. Next, the solution of tributylammonium pyrophosphate (98.8 mg, 0.18 mmol, 1.2 equiv.), in the mixture (1:3) of dry tributylamine (0.15 mL) and DMF (0.45 mL) was added dropwise and the reaction mixture was continued to stir at room temperature for another 1 hour. The mixture was then oxidized by the addition of iodine (49.5 mg, 0.195 mmol, 1.3 equiv.) in pyridine (0.9 mL) and H_2_O (0.3 mL). After 30 min of stirring, the excess iodine was quenched with sodium thiosulfate solution (10% w/v in water) and the reaction mixture was concentrated under reduced pressure at 30 °C. The residue was precipitated by the addition of 2% NaClO_4_ solution in acetone (10 mL), centrifuged, acetone was decanted and the precipitate was dried under reduced pressure. The crude precipitate was re-dissolved in water and purified by HPLC using a preparative ion exchange column (Buffer A: 10 mM TEAB, Buffer B: 1M TEAB). The aqueous fractions were collected and concentrated under reduced pressure at 30 °C, then converted to a sodium salt by loading on DOWEX Na^+^ ion-exchange column and washed with water (15 mL). Finally, the collected aqueous solution was freeze-dried yielding the corresponding triphosphates **1-7** as white lyophilizates in 10-26% yields.

**Supplementary Scheme 1.** Synthesis of 3’-protected DNA and LNA nucleoside triphosphates **1-7**.

**3’-*O*-Mesitoyl-thymidine-5’-*O*-triphosphate 1**

****Triphosphate **1** (21.0 mg, 0.034 mmol, 26%) was prepared from nucleoside **S2** (50.5 mg, 0.13 mmol) according to the general procedure. ^1^H NMR (500 MHz, D_2_O) δ 7.35 (d, *J* = 1.3 Hz, 1H), 6.97 (s, 2H), 6.31 (dd, *J* = 7.0, 4.7 Hz, 1H), 5.15 – 5.10 (m, 1H), 4.68 – 4.67 (m, 2H), 4.55 – 4.53 (m, 1H), 2.79 – 2.74 (m, 1H), 2.69 – 2.64 (m, 1H), 2.29 (s, 3H), 2.21 (s, 6H), 1.50 (d, *J* = 1.2 Hz, 3H). ^13^C NMR (126 MHz, D_2_O) δ 172.4, 166.1, 152.0, 140.8, 137.3, 135.0, 129.5, 128.3, 111.0, 85.5, 82.3, 74.8, 63.8, 36.2, 20.2, 18.6, 11.0. ^31^P NMR (203 MHz, D_2_O) δ -10.67 (d, *J* = 19.7 Hz), -12.19 (d, *J* = 19.5 Hz), -23.43 (t, *J* = 19.4 Hz). HRMS calcd for C_20_H_26_N_2_O_15_P_3_ [M-H]^-^: 627.0546, found: 627.0545.

**3’-*O*-Allyl-thymidine-5’-*O*-triphosphate 3**

****Triphosphate **3** (16.9 mg, 0.032 mmol, 19%) was prepared from nucleoside **S4** (48.0 mg, 0.17 mmol) according to the general procedure. ^1^H NMR (500 MHz, D_2_O) δ 7.78 (d, *J* = 1.3 Hz, 1H), 6.34 (dd, *J* = 8.9, 5.8 Hz, 1H), 6.06 – 5.95 (m, 1H), 5.40 (dq, *J* = 17.3, 1.6 Hz, 1H), 5.30 (dq, *J* = 10.4, 1.2 Hz, 1H), 4.51 – 4.45 (m, 1H), 4.40 – 4.34 (m, 1H), 4.22 (dd, *J* = 5.2, 3.4 Hz, 2H), 4.16 (dt, *J* = 5.8, 1.2 Hz, 2H), 2.49 (ddd, *J* = 14.3, 5.8, 1.7 Hz, 1H), 2.39 – 2.29 (m, 1H), 1.95 (d, *J* = 1.2 Hz, 3H). ^13^C NMR (126 MHz, D_2_O) δ 166.6, 151.7, 137.2, 133.6, 118.5, 111.8, 85.1, 83.5, 79.6, 70.2, 66.0, 36.3, 11.6. ^31^P NMR (203 MHz, D_2_O) δ -6.49 (d, *J* = 21.0 Hz), -11.75 (d, *J* = 19.4 Hz), -22.70 (t, *J* = 20.2 Hz). HRMS calcd for C_13_H_20_N_2_O_14_P_3_ [M-H]^-^: 521.0133, found: 521.0131.

**3’-*O*-Azidomethyl-thymidine-5’-*O*-triphosphate 5**

****Triphosphate **5** (17.0 mg, 0.032 mmol, 21%) was prepared from nucleoside **S5** (44.6 mg, 0.15 mmol) according to the general procedure. ^1^H NMR (500 MHz, D_2_O) δ 7.78 (d, *J* = 1.3 Hz, 1H), 6.35 (dd, *J* = 8.4, 5.3 Hz, 1H), 4.92 (d, *J* = 8.4 Hz, 1H), 4.87 (d, *J* = 9.1 Hz, 1H), 4.72 – 4.64 (m, 1H), 4.42 – 4.36 (m, 1H), 4.30 – 4.14 (m, 2H), 2.54 (ddd, *J* = 14.2, 5.9, 2.2 Hz, 1H), 2.49 – 2.37 (m, 1H), 1.95 (t, *J* = 1.3 Hz, 3H). ^13^C NMR (126 MHz, D_2_O) δ 166.5, 151.7, 137.2, 111.9, 85.0, 83.7, 81.5, 79.3, 65.6, 36.7, 11.3. ^31^P NMR (203 MHz, D_2_O) δ -9.59 (d, *J* = 20.0 Hz, 1P), -11.76 (d, *J* = 19.8 Hz, 1P), -23.1 (t, 19.9 Hz, 1P). HRMS calcd for C_11_H_17_N_5_O_14_P_3_ [M-H]^-^: 535.9985, found: 535.9992.

**3’-*O*-Mesitoyl-LNA-thymidine-5’-*O*-triphosphate 2**

****Triphosphate **2** (22.2 mg, 0.034 mmol, 26%) was prepared from nucleoside **S7** (54.1 mg, 0.13 mmol) according to the general procedure. ^1^H NMR (500 MHz, D_2_O) δ 7.81 (d, *J* = 1.4 Hz, 1H), 7.04 (s, 2H), 5.84 (s, 1H), 5.25 (s, 1H), 4.96 (s, 1H), 4.51 (qd, *J* = 12.5, 6.3 Hz, 2H), 4.22 – 4.15 (m, 2H), 2.31 (s, 6H), 2.30 (s, 3H), 1.98 (d, *J* = 1.2 Hz, 3H). ^13^C NMR (126 MHz, D_2_O) δ 170.8, 166.6, 151.0, 141.5, 136.0, 135.9, 128.4, 128.2, 111.1, 86.9, 86.8, 77.6, 72.3, 72.0, 61.0, 20.2, 18.9, 11.8. ^31^P NMR (203 MHz, D_2_O) δ -10.34 (d, *J* = 19.7 Hz), -11.86 (d, *J* = 19.5 Hz), -23.11 (t, *J* = 19.4 Hz). HRMS calcd for C_21_H_26_N_2_O_16_P_3_ [M-H]^-^: 655.0495, found: 655.0495.

**3’-*O*-Allyl-LNA-thymidine-5’-*O*-triphosphate 4**

****Triphosphate **4** (18.7 mg, 0.034 mmol, 20%) was prepared from nucleoside **S9** (52.8 mg, 0.17 mmol) according to the general procedure. ^1^H NMR (500 MHz, D_2_O) δ 7.71 (d, *J* = 1.3 Hz, 1H), 6.03 – 5.92 (m, 1H), 5.72 (s, 1H), 5.41 – 5.33 (m, 1H), 5.33 – 5.25 (m, 1H), 4.66 (s, 1H), 4.52 – 4.44 (m, 2H), 4.24 – 4.18 (m, 2H), 4.13 – 4.11 (m, 2H), 4.08 – 4.05 (m, 1H), 1.94 (d, *J* = 1.2 Hz, 3H). ^13^C NMR (126 MHz, D_2_O) δ 168.1, 152.1, 136.0, 133.4, 118.8, 110.8, 87.0, 86.7, 77.4, 76.2, 71.6, 61.2, 58.9, 11.9. ^31^P NMR (203 MHz, D_2_O) -6.48 (d, *J* = 20.9 Hz), -11.80 (d, *J* = 19.2 Hz), -22.60 (t, *J* = 20.0 Hz). HRMS calcd for C_14_H_20_N_2_O_15_P_3_ [M-H]^-^: 549.0077, found: 549.0081.

**3’-*O*-Methyl-LNA-thymidine-5’-*O*-triphosphate 6**

****Triphosphate **6** (28.3 mg, 0.054 mmol, 24%) was prepared from nucleoside **S11** (64.0 mg, 0.225 mmol) according to the general procedure. ^1^H NMR (500 MHz, D_2_O) δ 7.75 (d, *J* = 1.3 Hz, 1H), 5.72 (s, 1H), 4.69 (s, 1H), 4.51 – 4.43 (m, 2H), 4.08 – 4.02 (m, 2H), 4.01 (s, 1H), 3.49 (s, 3H), 1.95 (d, *J* = 1.3 Hz, 3H). ^13^C NMR (126 MHz, D_2_O) δ 166.6, 151.0, 136.2, 111.0, 86.3, 77.9, 76.8, 71.6, 61.2, 58.9, 46.6, 11.8. ^31^P NMR (203 MHz, D_2_O) -6.74 (d, *J* = 20.6 Hz), -11.67 (d, *J* = 19.1 Hz), -22.48 (t, *J* = 19.8 Hz). HRMS calcd for C_12_H_18_N_2_O_15_P_3_ [M-H]^-^: 522.9920, found: 522.9924.

**3’-*O*-Azidomethyl-LNA-thymidine-5’-*O*-triphosphate 7**

****Triphosphate **7** (5.0 mg, 0.009 mmol, 10%) was prepared from nucleoside **S14** (29.3 mg, 0.09 mmol) according to the general procedure. ^1^H NMR (500 MHz, D_2_O) δ 7.61 (s, 1H), 5.59 (s, 1H), 4.57 (s, 1H), 4.37 – 4.28 (m, 2H), 4.10 (s, 1H), 3.97 (d, *J* = 8.9 Hz, 1H), 3.91 (d, *J* = 8.7 Hz, 1H), 1.80 (d, *J* = 1.2 Hz, 2H), 1.77 (s, 3H). ^31^P NMR (203 MHz, D_2_O) -13.68 (d, *J* = 19.8 Hz), -14.83 (d, *J* = 19.8 Hz), -26.31 (t, *J* = 19.8 Hz). HRMS calcd for C_12_H_17_N_5_O_15_P_3_ [M-H]^-^: 563.9939, found: 563.9926.

# Copies of NMR spectra

**^1^H NMR (D_2_O, 500 MHz)** spectrum of **3’-*O*-Mesitoyl-thymidine-5’-*O*-triphosphate 1.**


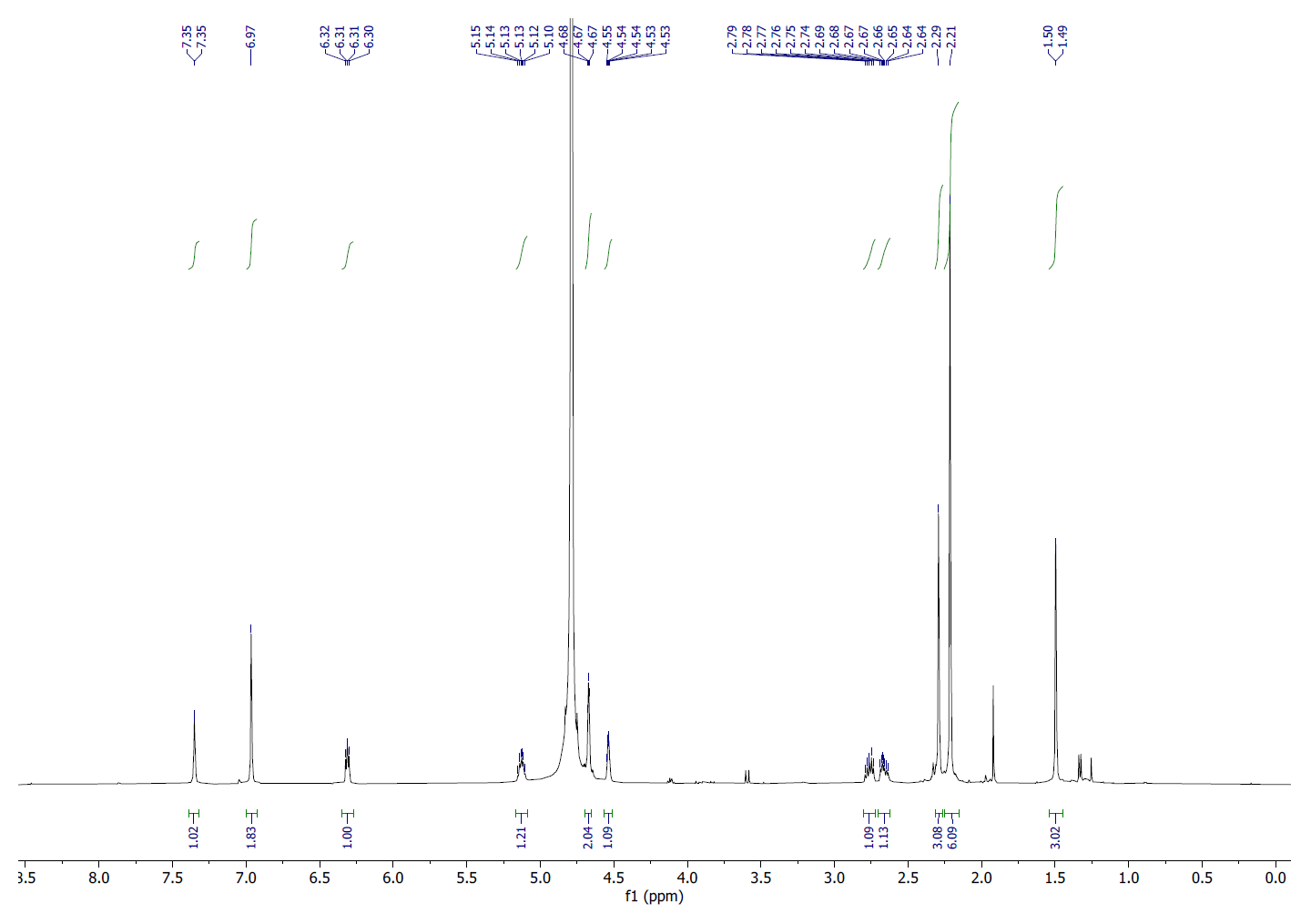


**^13^C NMR (D_2_O, 126 MHz)** spectrum of **3’-*O*-Mesitoyl-thymidine-5’-*O*-triphosphate 1.**


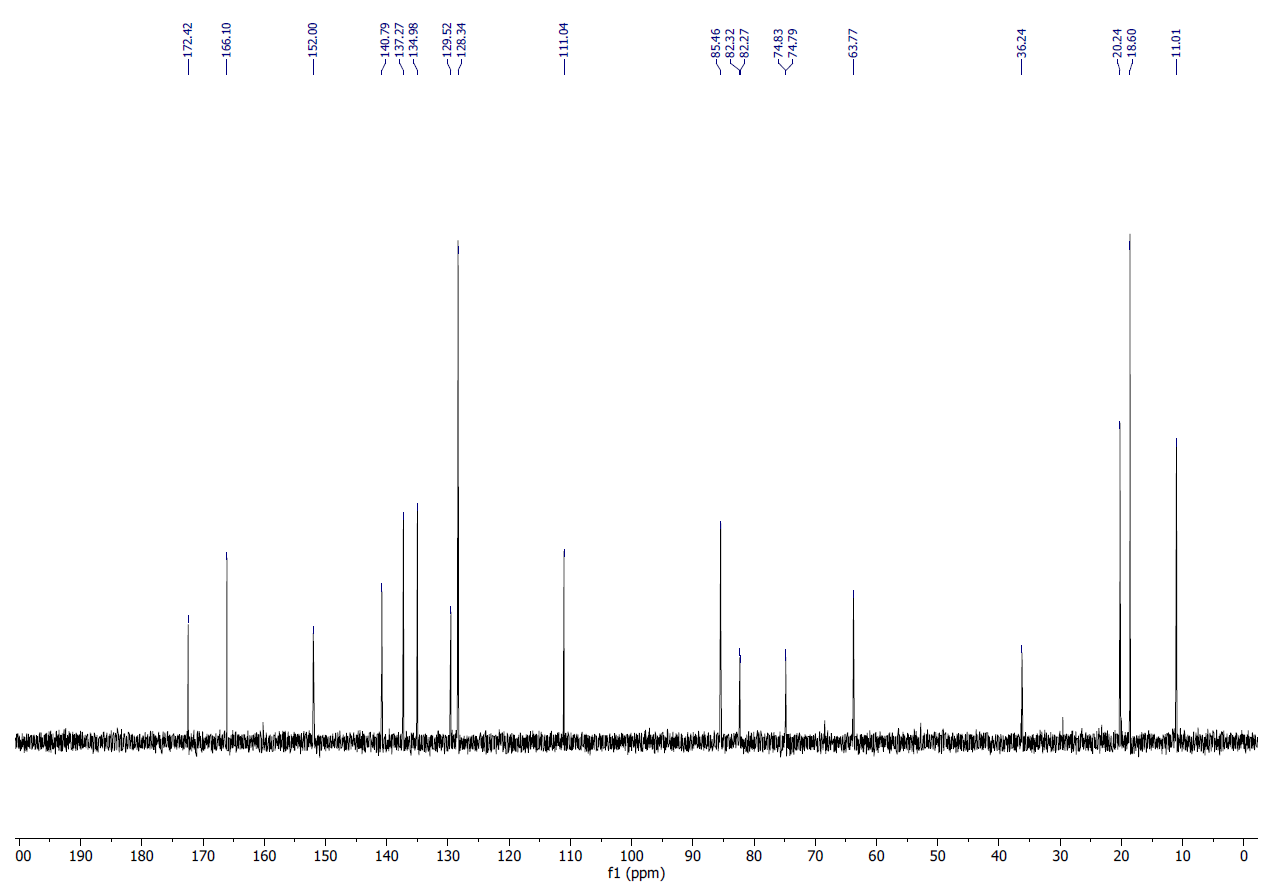


**^31^P NMR (D_2_O, 203 MHz)** spectrum of **3’-*O*-Mesitoyl-thymidine-5’-*O*-triphosphate 1.**


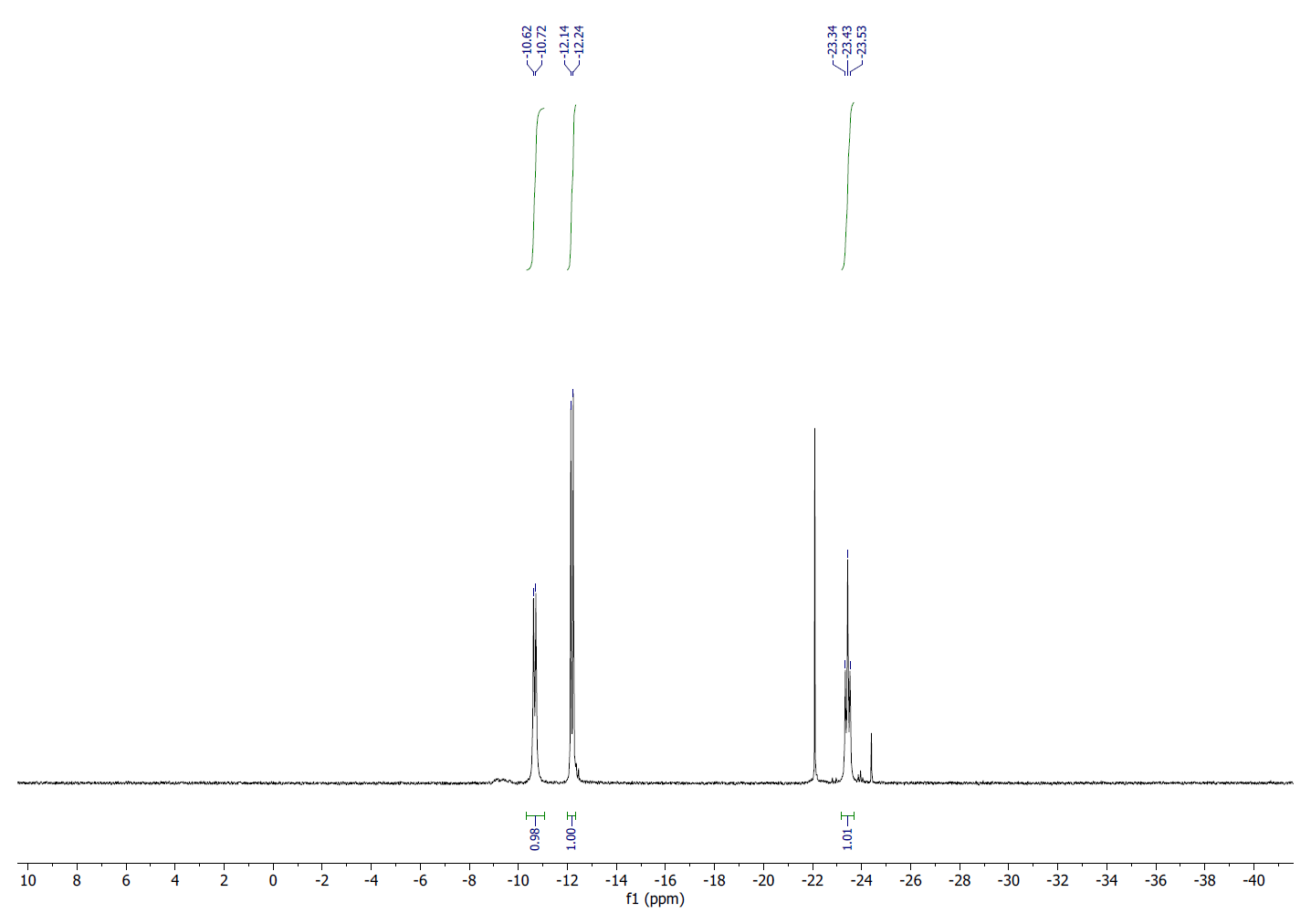


**^1^H NMR (D_2_O, 500 MHz)** spectrum of **3’-*O*-Allyl-thymidine-5’-*O*-triphosphate 3.**


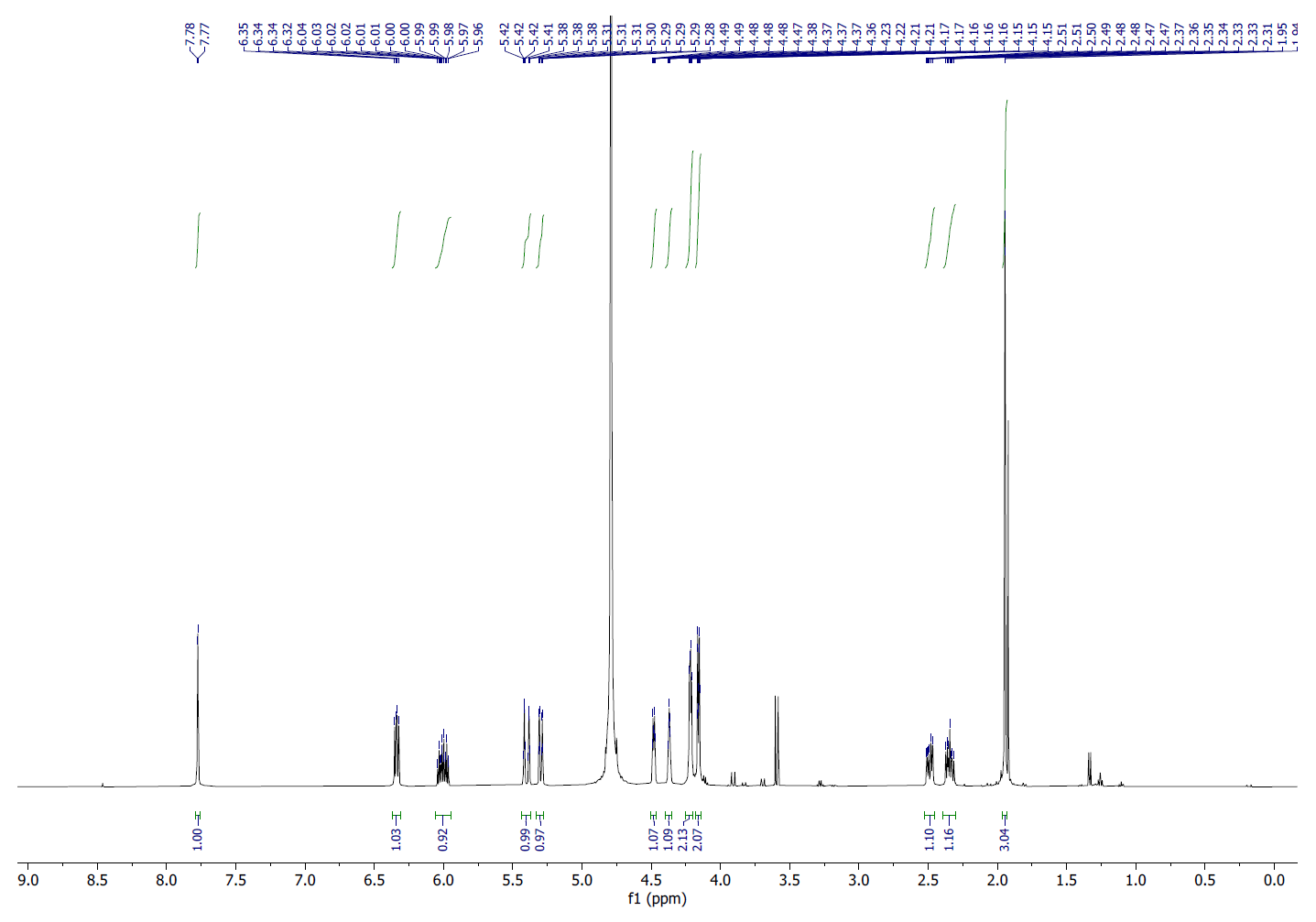


**^13^C NMR (D_2_O, 126 MHz)** spectrum of **3’-*O*-Allyl-thymidine-5’-*O*-triphosphate 3.**


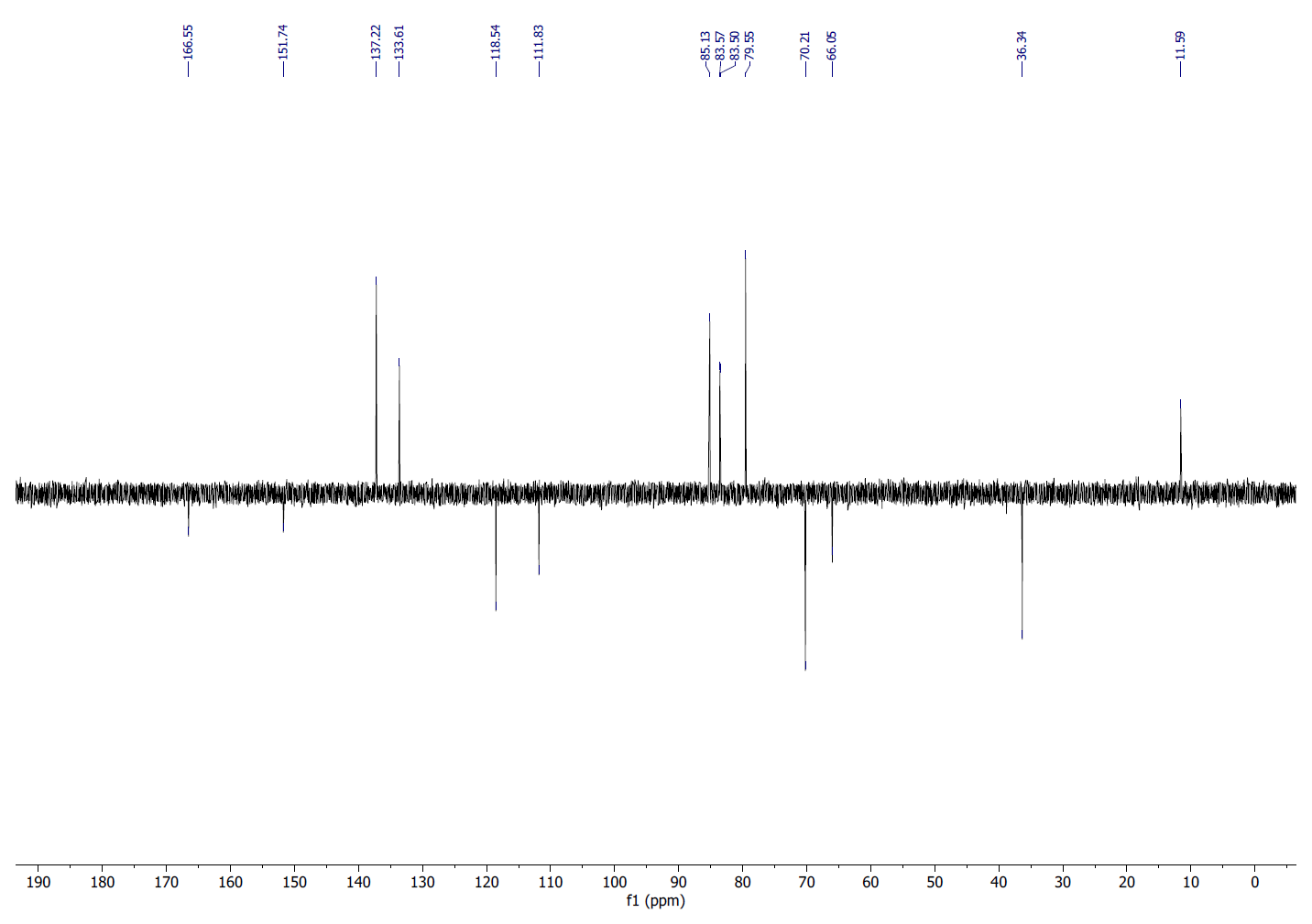


**^31^P NMR (D_2_O, 203 MHz)** spectrum of **3’-*O*-Allyl-thymidine-5’-*O*-triphosphate 3.**


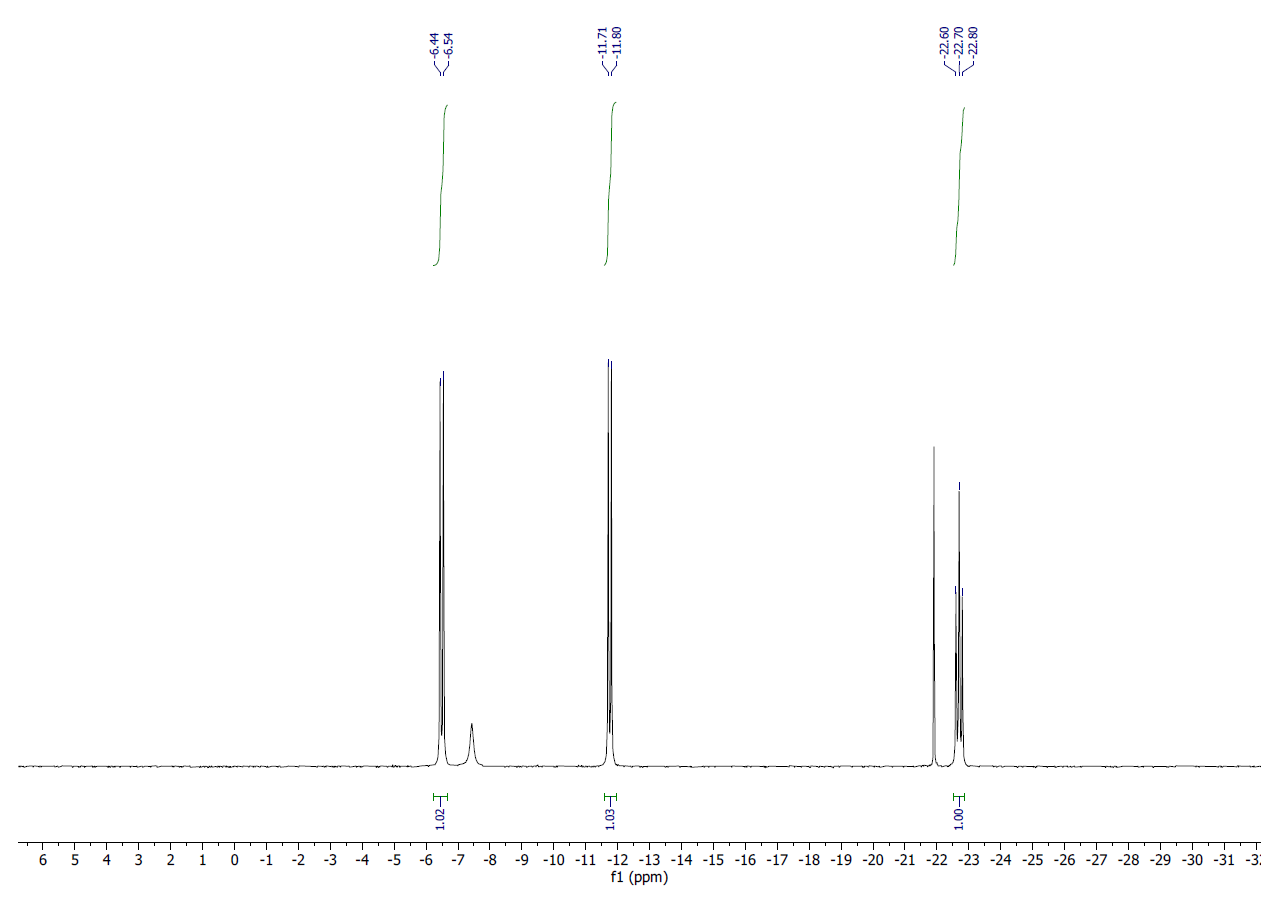


**^1^H NMR (D_2_O, 500 MHz)** spectrum of **3’-*O*-Azidomethyl-thymidine-5’-*O*-triphosphate 5.**


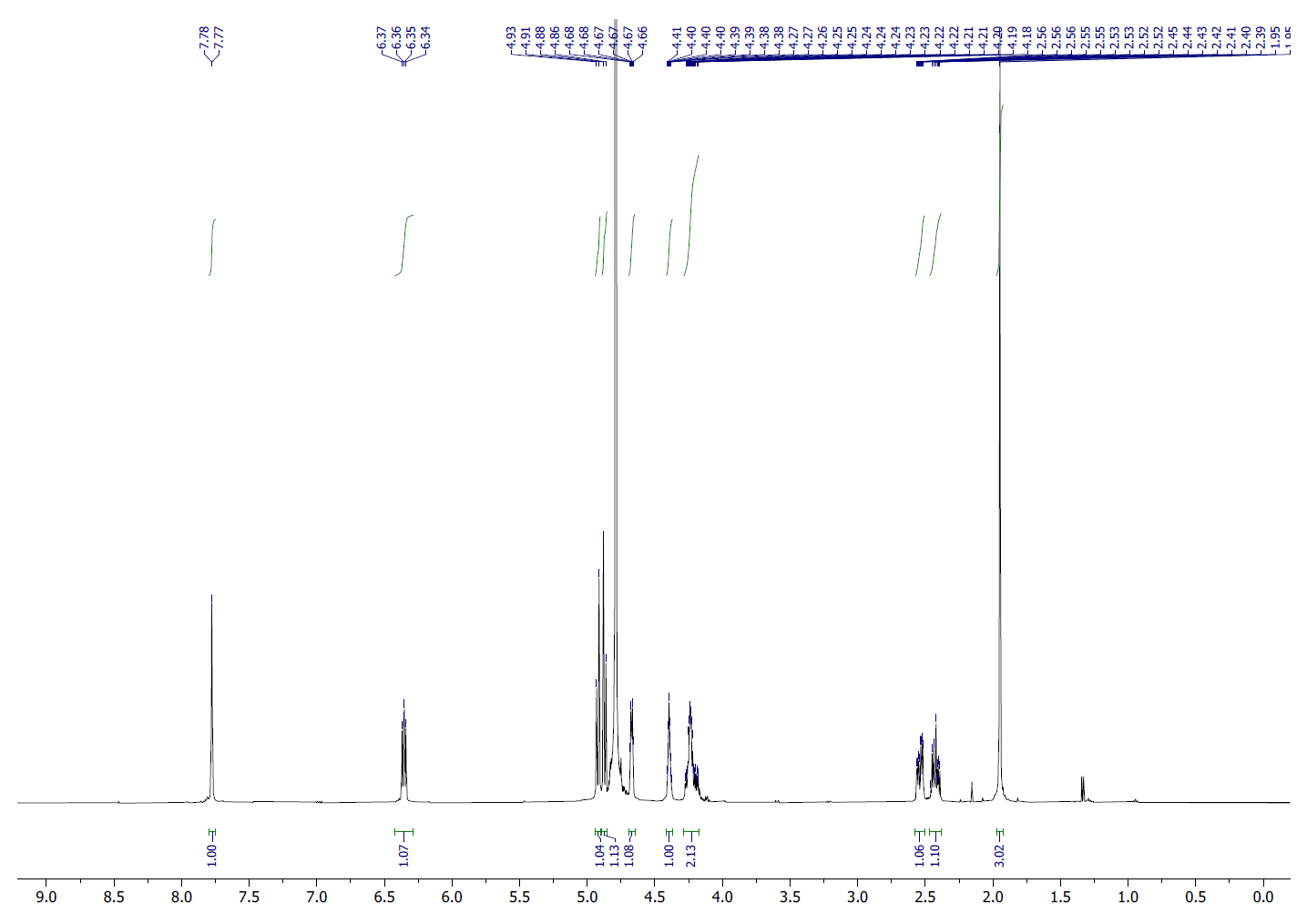


**^13^C NMR (D_2_O, 126 MHz)** spectrum of **3’-*O*-Azidomethyl-thymidine-5’-*O*-triphosphate 5.**


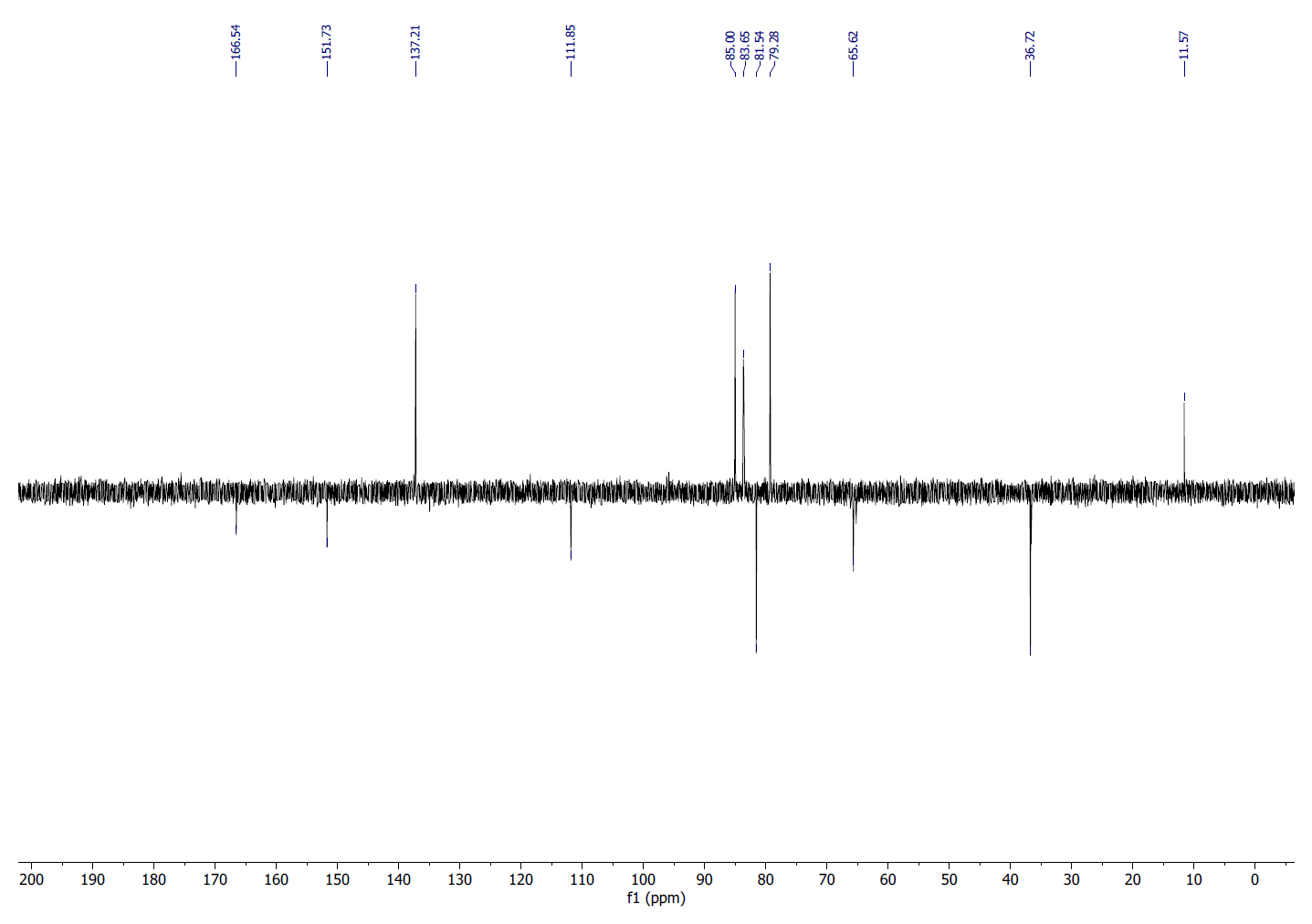


**^31^P NMR (D_2_O, 203 MHz)** spectrum of **3’-*O*-Azidomethyl-thymidine-5’-*O*-triphosphate 5.**


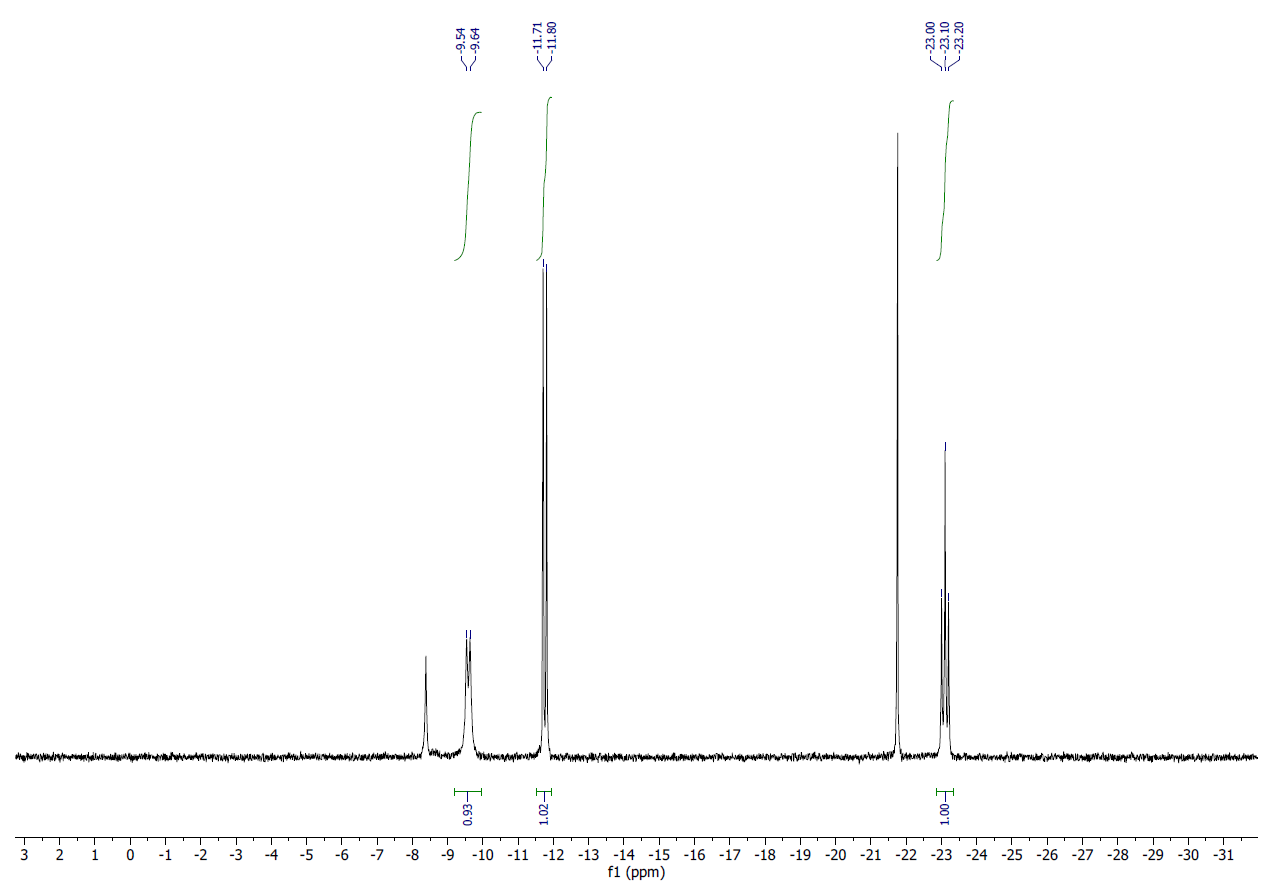


**^1^H NMR (D_2_O, 500 MHz)** spectrum of **3’-*O*-Mesitoyl-LNA-thymidine-5’-*O*-triphosphate 2.**


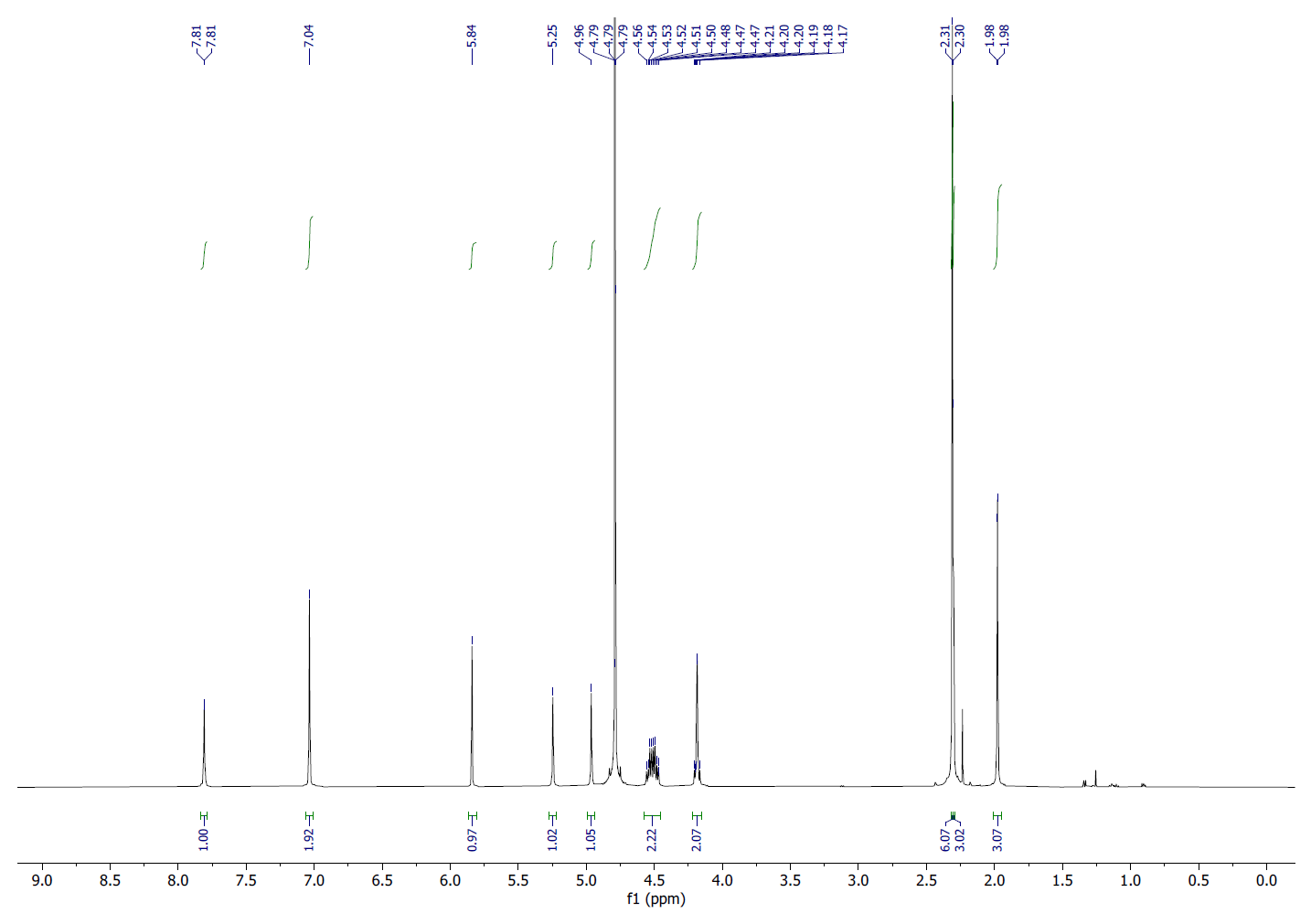


**^13^C NMR (D_2_O, 126 MHz)** spectrum of **3’-*O*-Mesitoyl-LNA-thymidine-5’-*O*-triphosphate 2.**


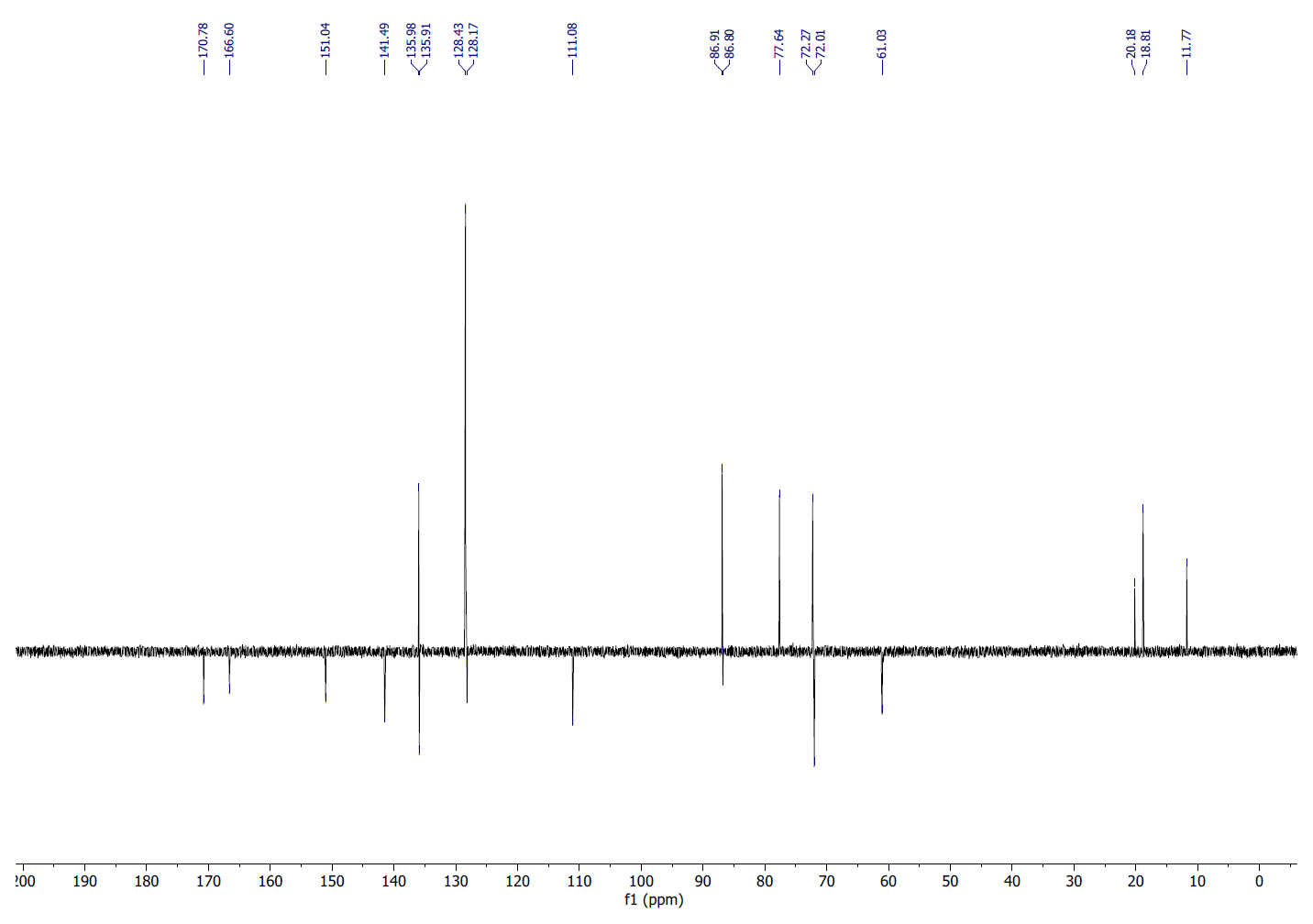


**^31^P NMR (D_2_O, 203 MHz)** spectrum of **3’-*O*-Mesitoyl-LNA-thymidine-5’-*O*-triphosphate 2.**


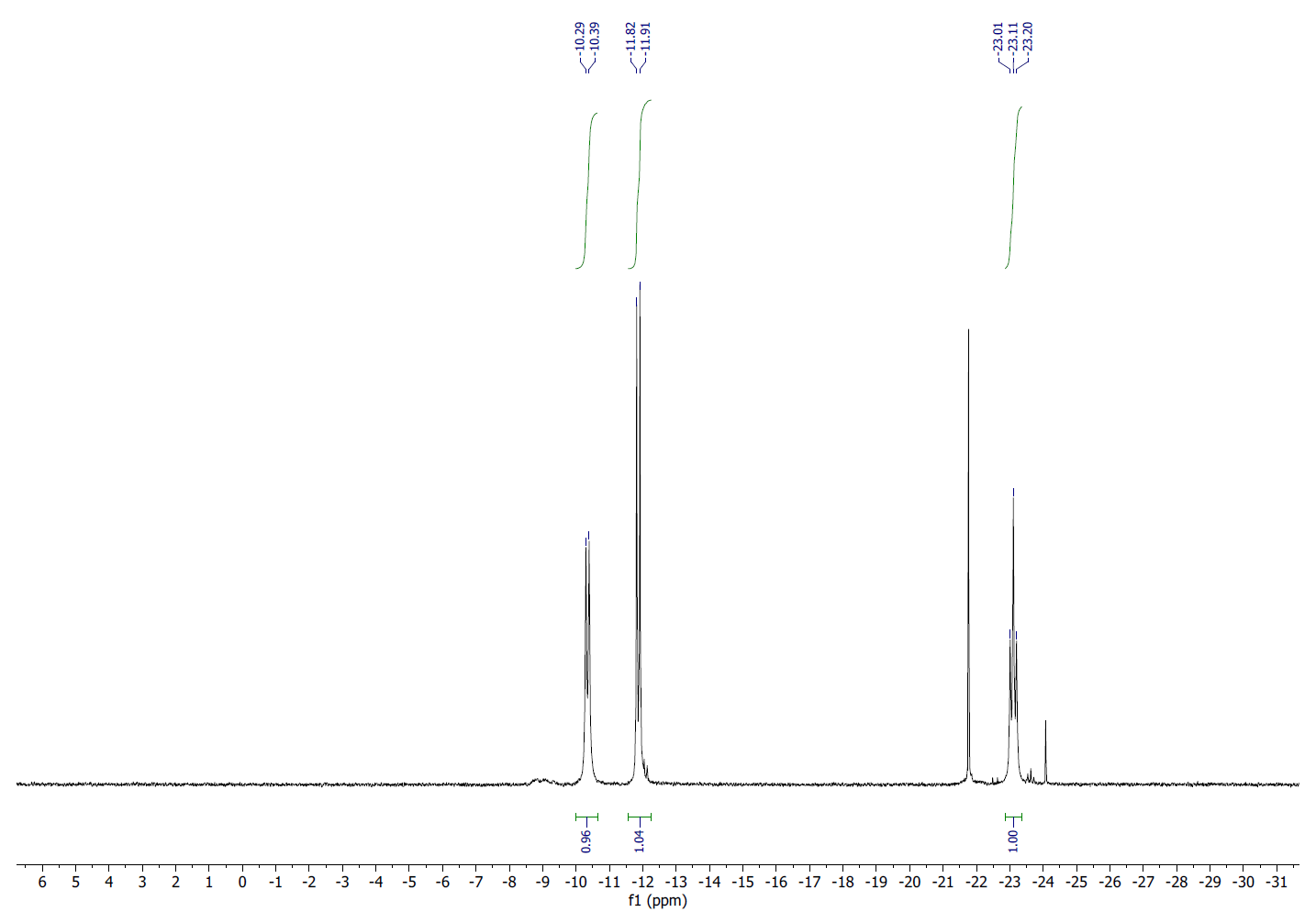


**^1^H NMR (D_2_O, 500 MHz)** spectrum of **3’-*O*-Allyl-LNA-thymidine-5’-*O*-triphosphate 4.**


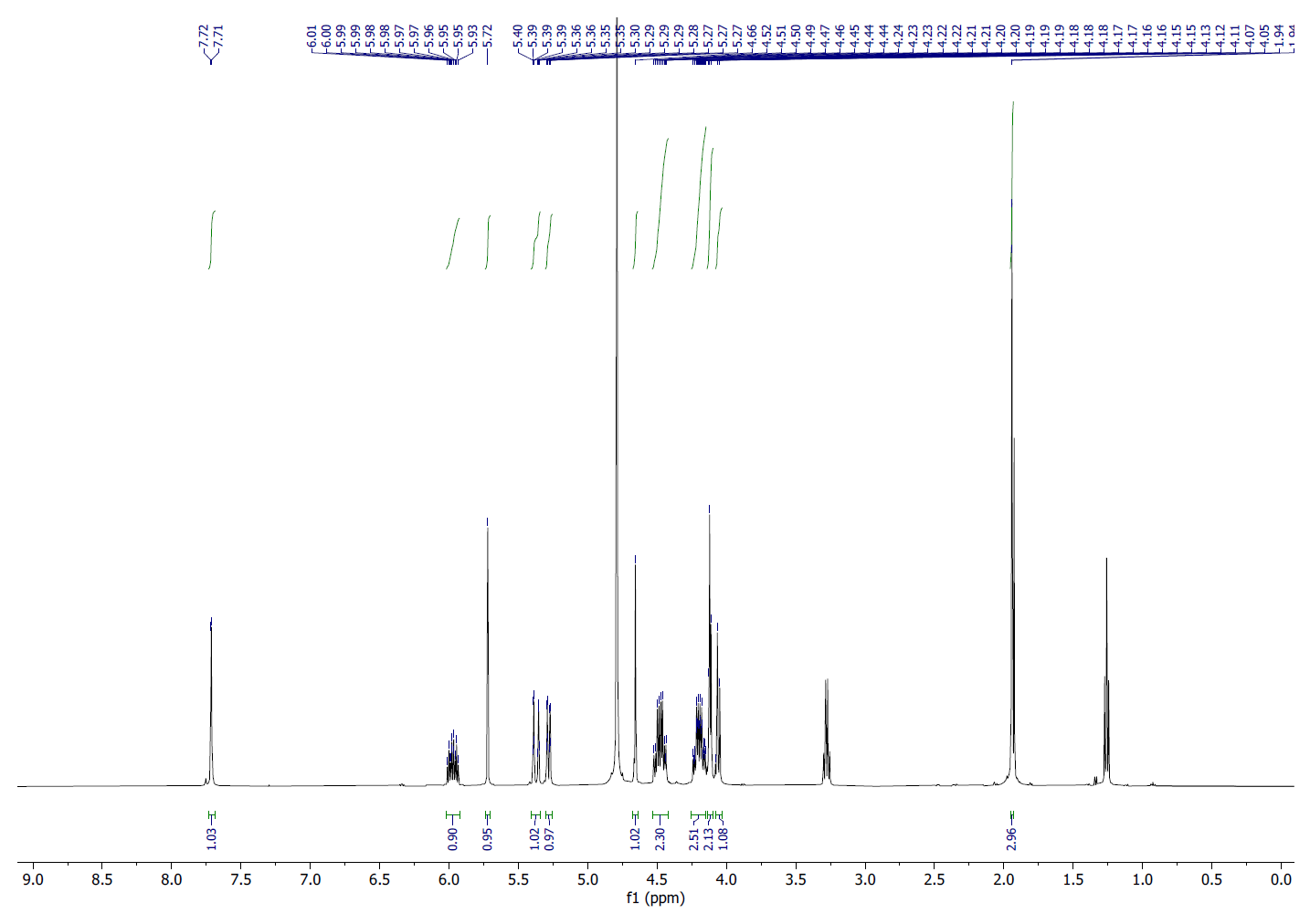


**^13^C NMR (D_2_O, 126 MHz)** spectrum of **3’-*O*-Allyl-LNA-thymidine-5’-*O*-triphosphate 4.**


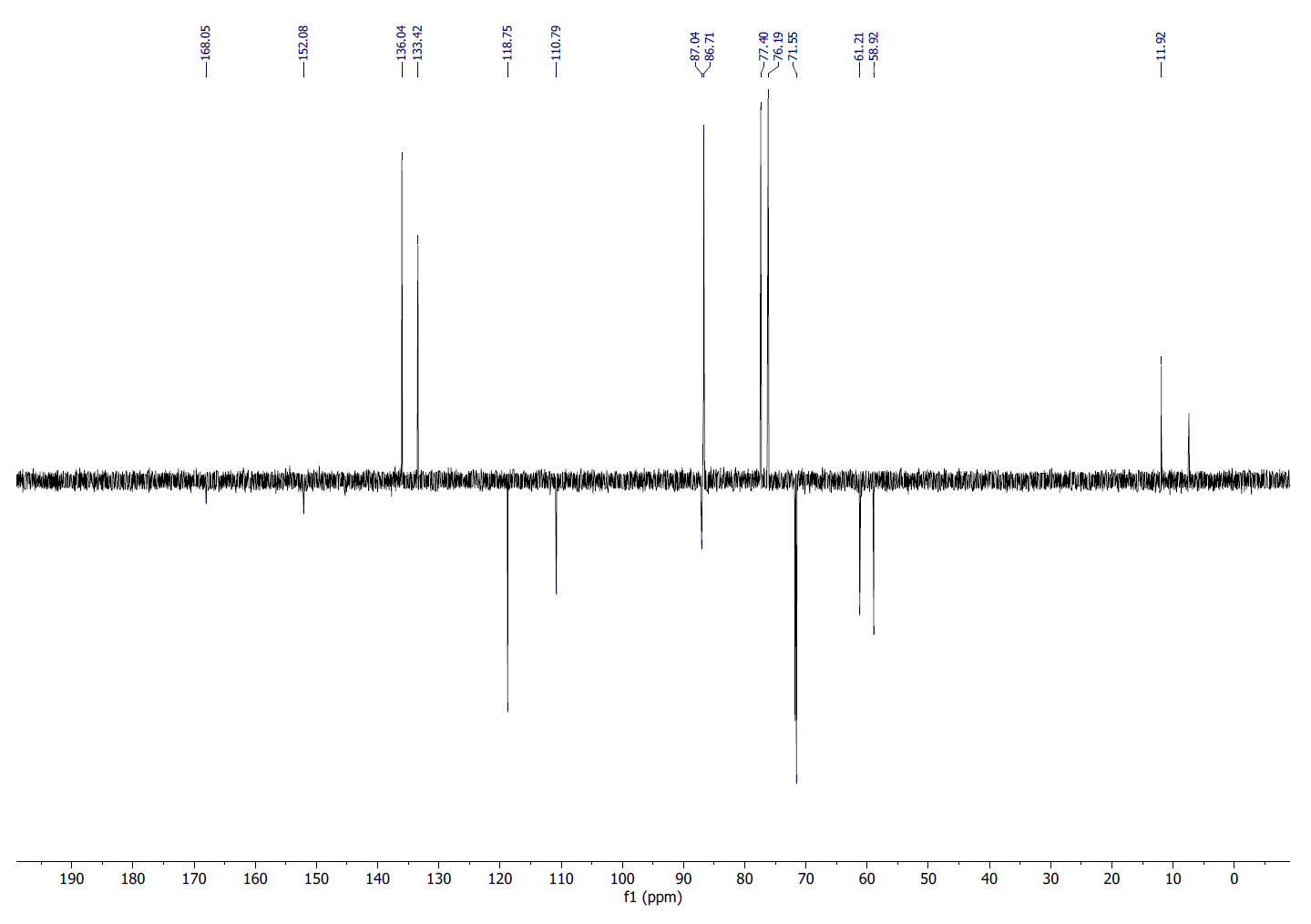


**^31^P NMR (D_2_O, 203 MHz)** spectrum of **3’-*O*-Allyl-LNA-thymidine-5’-*O*-triphosphate 4.**


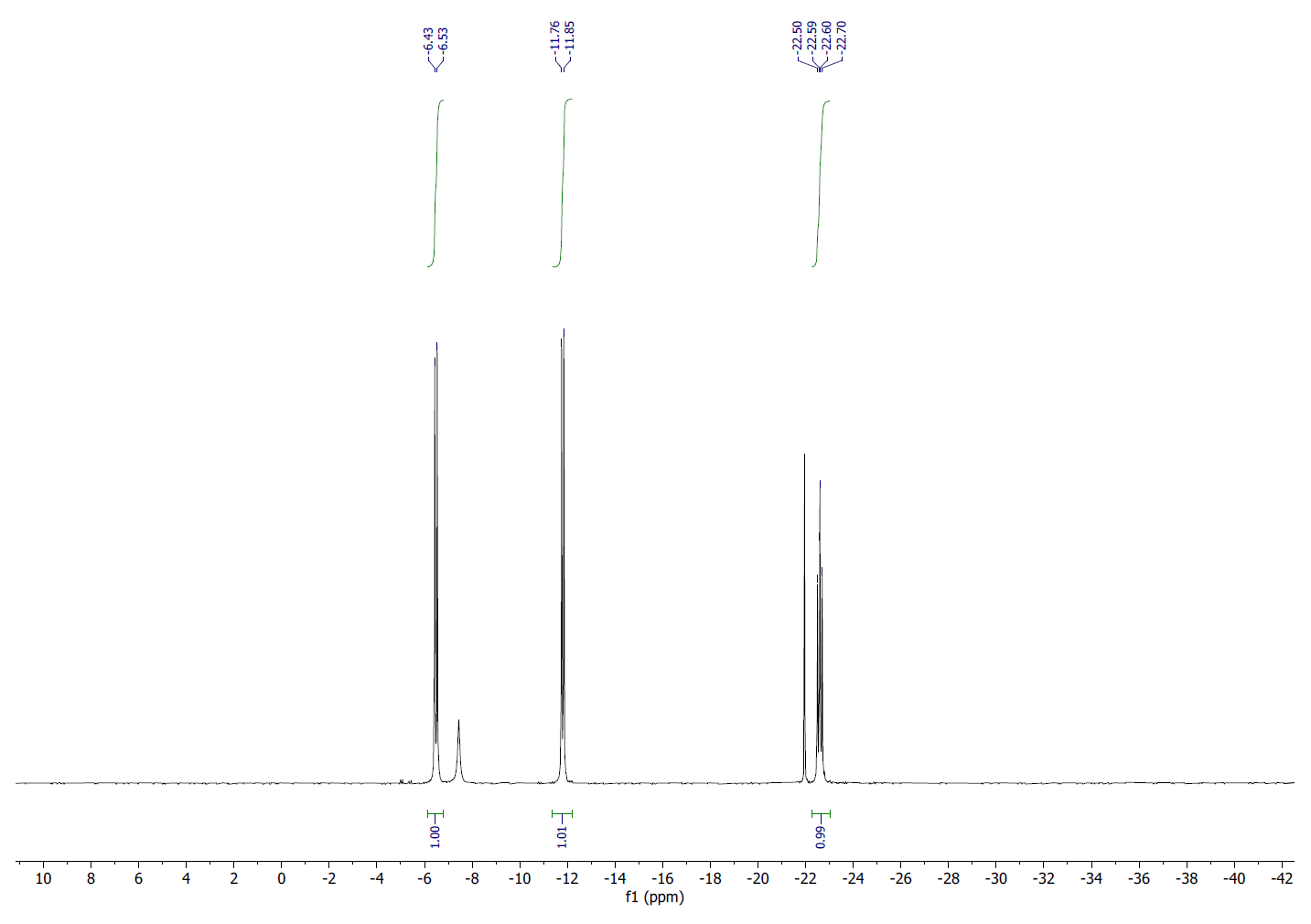


**^1^H NMR (D_2_O, 500 MHz)** spectrum of **3’-*O*-Methyl-LNA-thymidine-5’-*O*-triphosphate 6.**


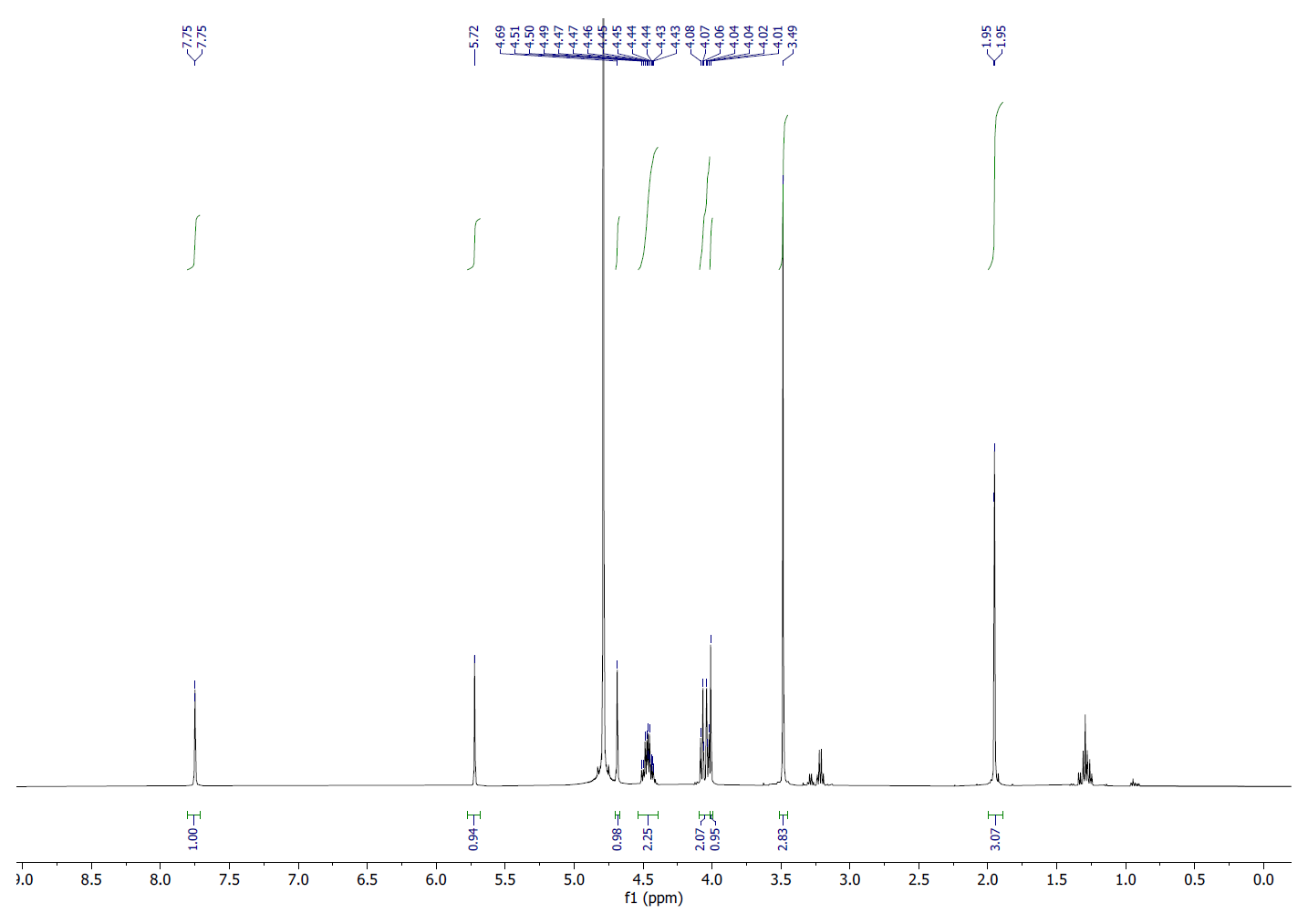


**^13^C NMR (D_2_O, 126 MHz)** spectrum of **3’-*O*-Methyl-LNA-thymidine-5’-*O*-triphosphate 6.**


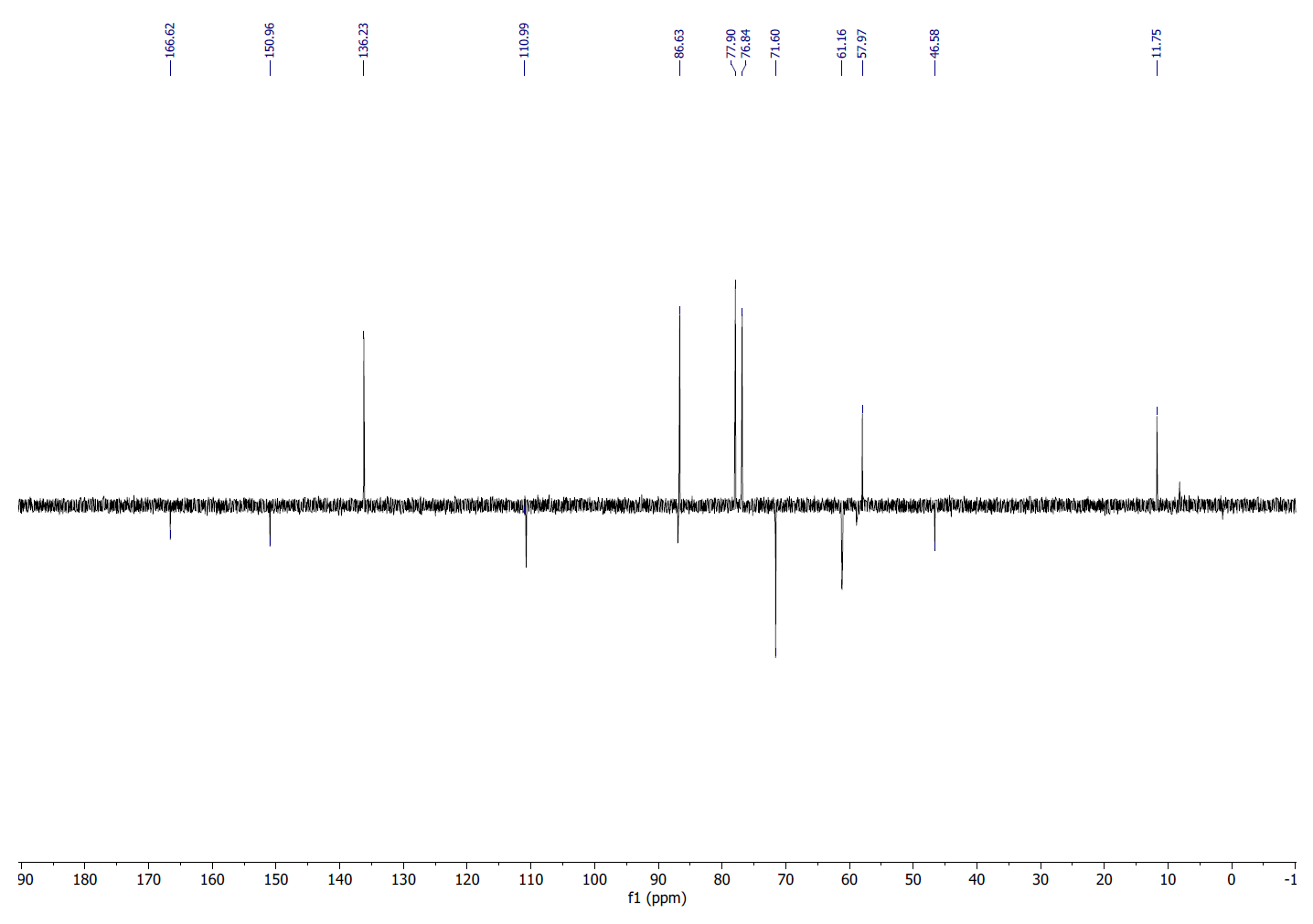


**^31^P NMR (D_2_O, 203 MHz)** spectrum of **3’-*O*-Methyl-LNA-thymidine-5’-*O*-triphosphate 6.**


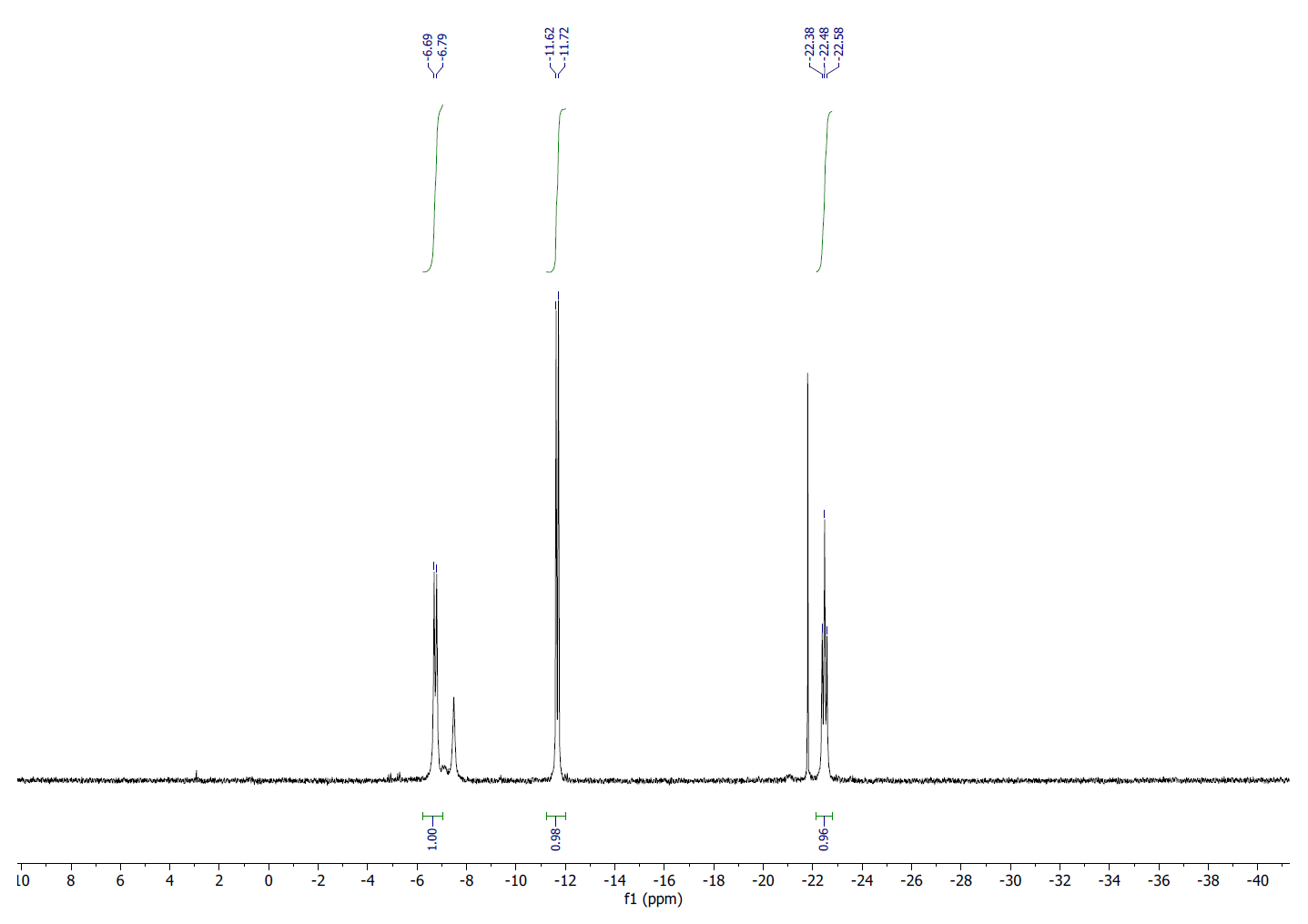


**^1^H NMR (D_2_O, 500 MHz)** spectrum of **3’-*O*-Azidomethyl-LNA-thymidine-5’-*O*-triphosphate 7.**


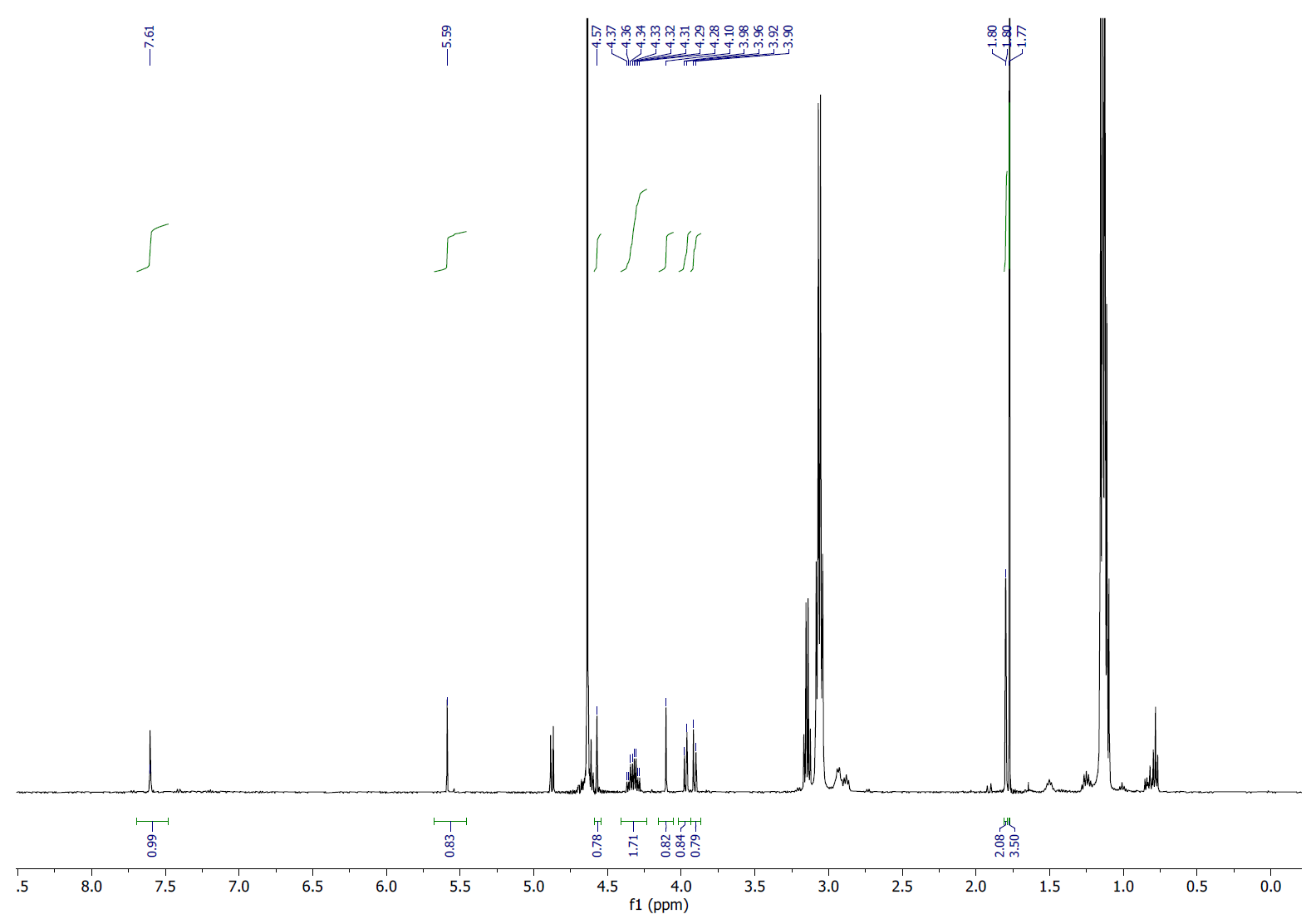


**^31^P NMR (D_2_O, 203 MHz)** spectrum of **3’-*O*-Azidomethyl-LNA-thymidine-5’-*O*-triphosphate 7.**


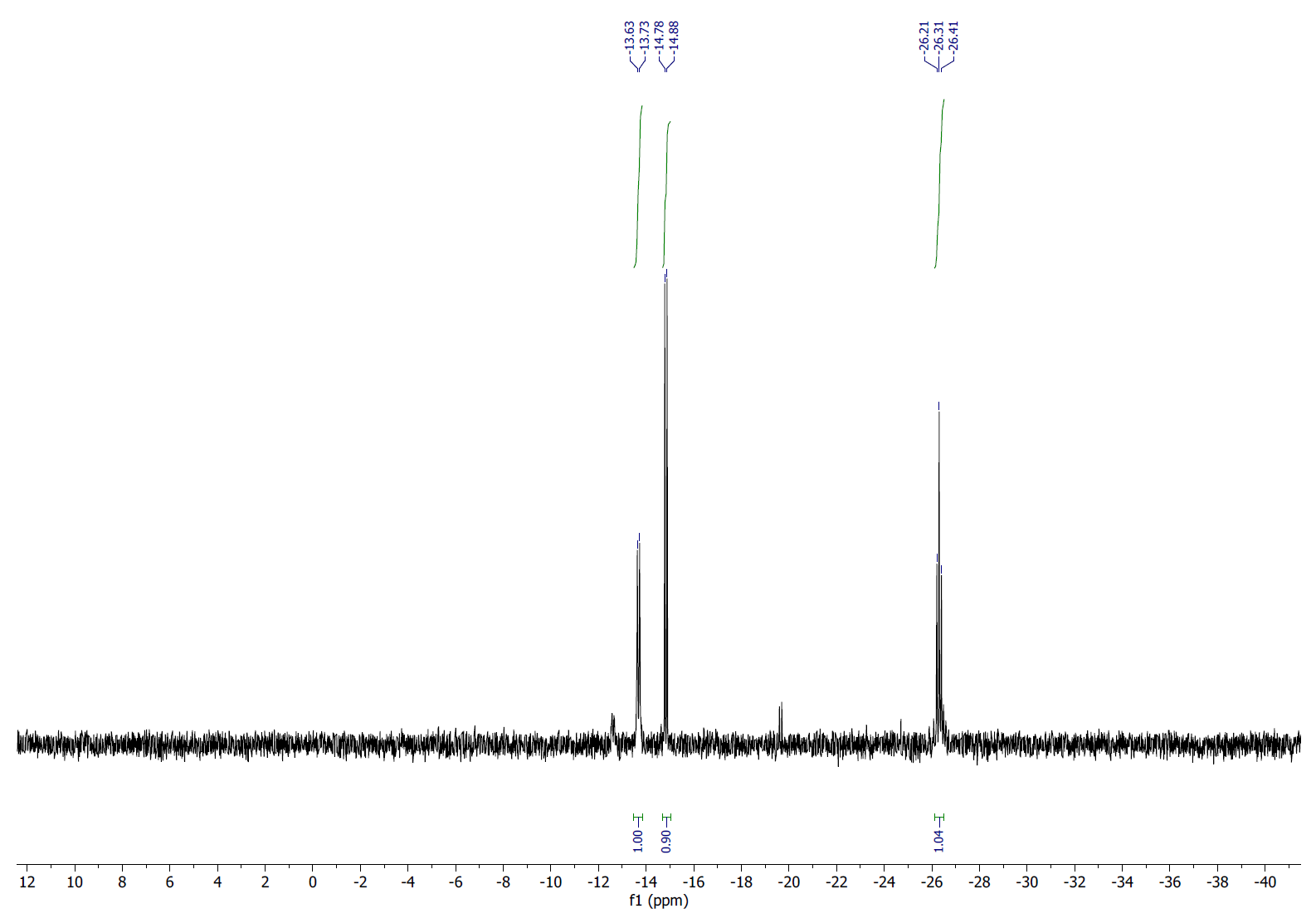


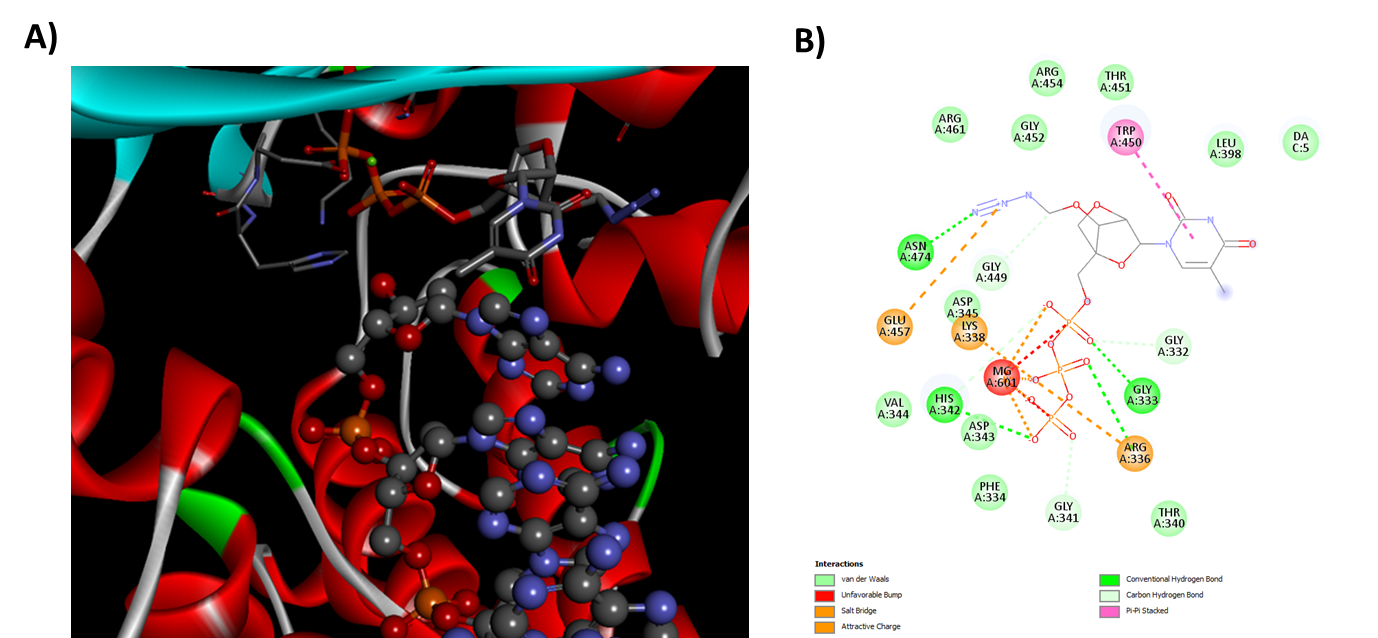


**Supplementary Figure 1:** A) Docking results obtained with 3′-*O*-azidomethyl-LNA-TTP **7** and the binary complex of mouse TdT and a ssDNA primer (PDB 4I27). B) 2D diagram of docking simulation showing the interactions between the 3′-*O*-azidomethyl-LNA-TTP **7** and the amino acids in the active site of the TdT polymerase. For experimental details and protocols see e.g. *Commun. Chem.* 2022, 5**,** 68 and *Asian J. Org. Chem.* 2022, 11**,** e202200384.

# Enzymatic reactions. General protocols.

## 4a. TdT reactions

**Commercial DNA Primer** (ON1) **used:** 5'-FAM-TAC GAC TCA CTA TAG CCT C -3' (19 nt), MW: 6244.

The optimization process of reaction conditions is summarized on the Supplementary Figure 2.


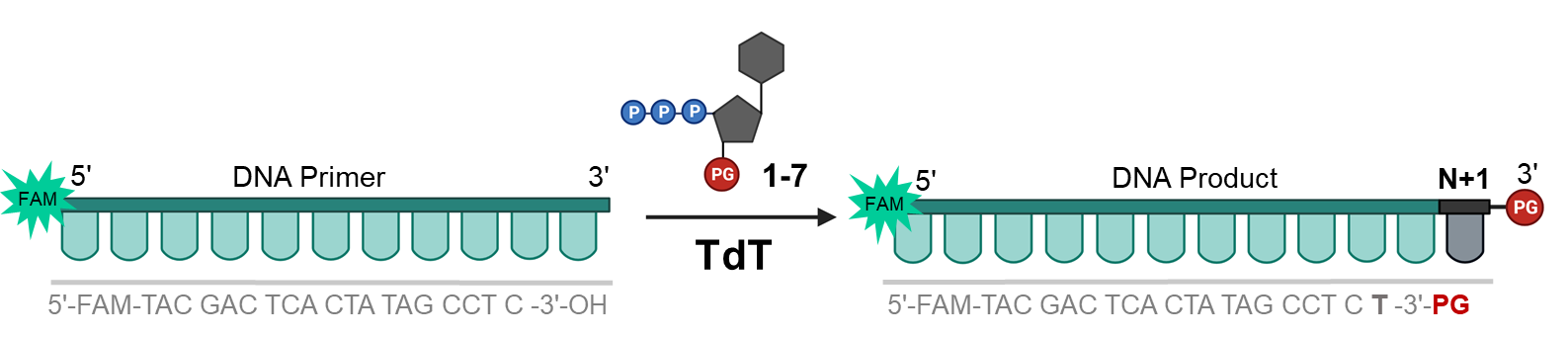


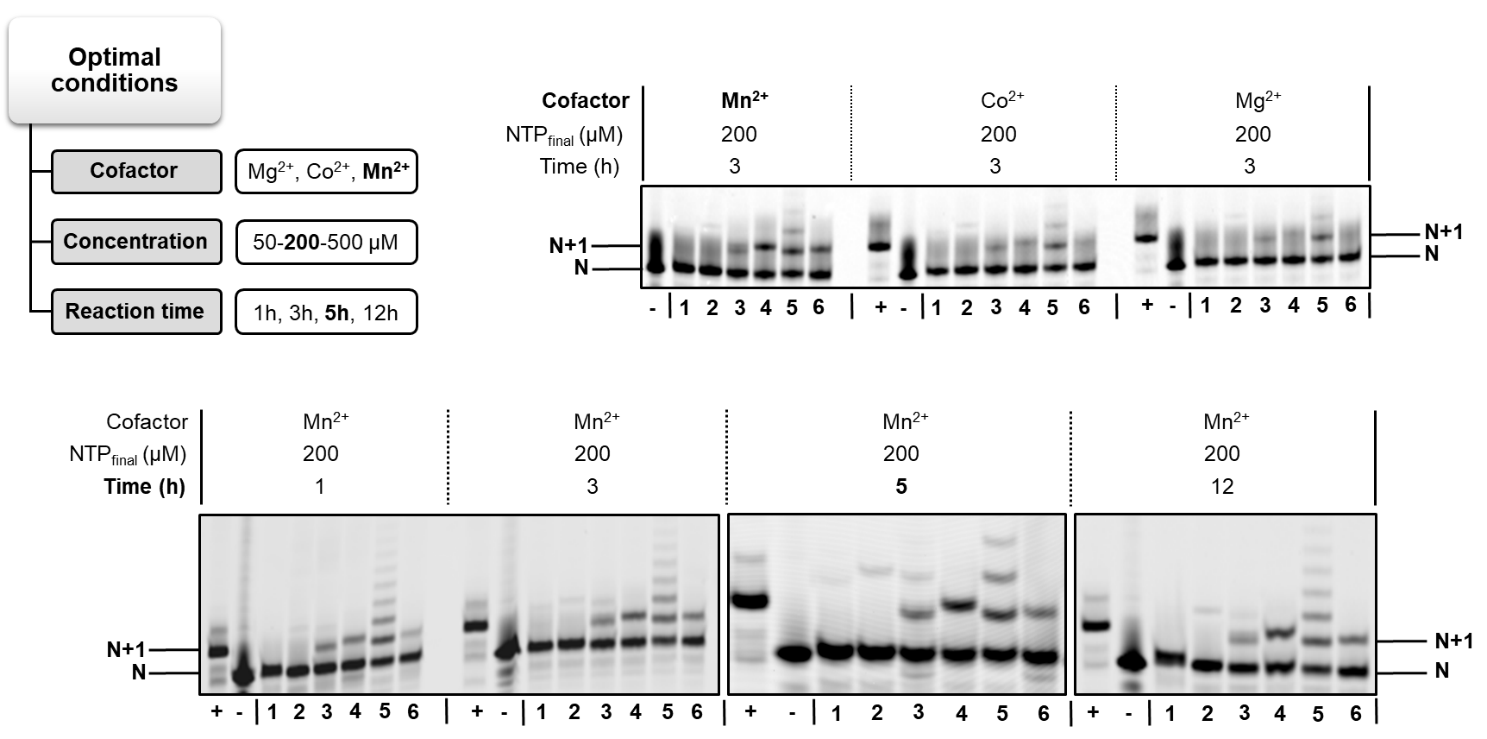


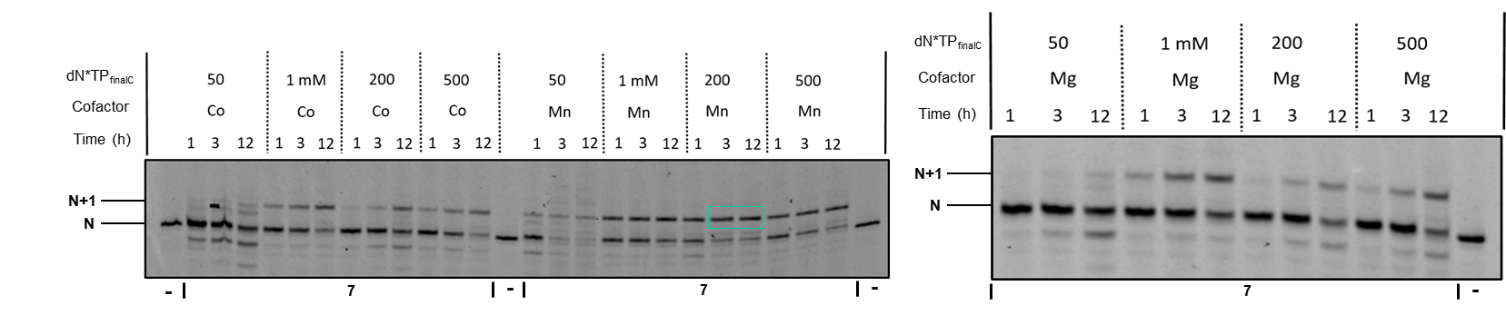


**Supplementary Figure 2.** Gel images (PAGE 20%) for analysis of TdT reactions. (**+**) – positive control using 3’-OH-LNA-dN*TP, (**-**) – negative control in the absence of TdT, (**1-7**) – 3'-OPG-dN*TPs.

**Analytical scale optimal conditions (10 µL):** DNA Primer ON1 10µM (1 µL), 3'-OPG-dN*TP **1-7** 1mM (2 µL), TdT buffer 10X (1 µL), Water (4 µL), TdT enzyme (0.5 µL), TIPP (0.5 µL), MnCl_2_ 10 mM (1 µL). Reaction mixtures were incubated at 37 °C during 5 hours.

**Preparative scale optimal conditions (100 µL):** DNA Primer ON1 100µM (10 µL), 3'-OPG-dN*TP **4, 6, 7** 1mM (60 µL), TdT buffer 10X (10 µL), TdT enzyme (10 µL), MnCl_2_ 20 mM (10 µL). Reaction mixtures were incubated at 37 °C during 5 hours.

## 4b. PUP reactions

**RNA Primer** (FLA) **used:**  5'-FAM-rCrArG rUrCrG rGrArU rCrGrC rArGrU rCrArG (18 nt), MW: 6308.0.

The optimization process of reaction conditions is summarized on the Supplementary Figure 3.


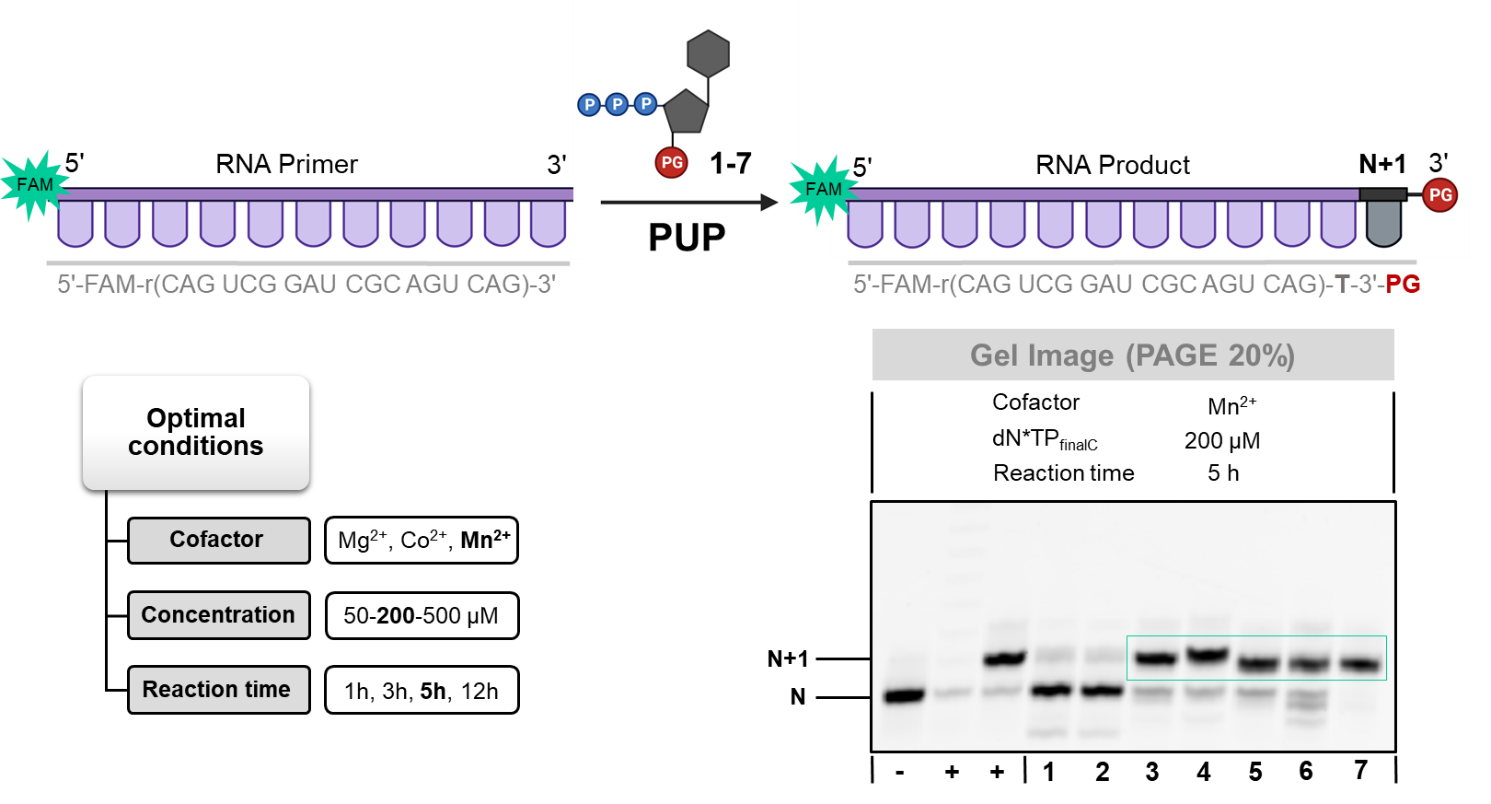


**Supplementary Figure 3.** Gel image (PAGE 20%) for analysis of PUP reactions. First from the left (**+**) – positive control using UTP, second from the left (**+**) – positive control using 3’-OH-LNA-dN*TP, (**-**) – negative control in the absence of PUP, (**1-7**) – 3'-OPG-dN*TPs.

**Analytical scale optimal conditions (10 µL):** RNA Primer FLA 10µM (2 µL), 3'-OPG-dN*TP **1-7** 1mM (2 µL), NEBuffer 2 10X (1 µL), Water (3 µL), PolyU Polymerase (0.5 µL), RNAse Inhibitor Murine (0.5 µL), MnCl_2_ 10 mM (1 µL). Reaction mixtures were incubated at 37 °C during 5 hours.

**Preparative scale optimal conditions (100 µL):** RNA Primer FLA 10µM (25 µL), 3'-OPG-dN*TP **3-7** 1mM (25 µL), NEBuffer 2 10X (10 µL), Water (20 µL), PolyU Polymerase (5 µL), RNAs Inhibitor Murine (5 µL), MnCl_2_ 10 mM (10 µL). Reaction mixtures were incubated at 37 °C during 5 hours.

## 4c. PEX reactions

**DNA primer** (MH5) **used:** 5'-FAM-TAC GAC TCA CTA TAG CCT C -3' (15 nt), MW: 5211.5.

**DNA Template** (MH4A) **used:** 5'-AAA AAA ACC CAT GCC GCC CAT G -3' (22 nt), MW: 6666.0.

The optimization process of reaction conditions is summarized on the Supplementary Figures 4a-e.

**Analytical scale optimal conditions (10 µL):** DNA Primer MH5 10µM (1 µL), DNA Template MH4A 10µM (1.5 µL), Water (0.5 µL) were mixed and hybridized by incubation program 95 °C (5 minutes) to 62 °C (5 minutes) to 25 °C. Next, other components of the reaction were added depending on DNA polymerase used:

1. **Hemo KlenTaq** polymerase ( (1 µL), Hemo KlenTaq buffer 5X (2 µL), 3'-OPG-dN*TP **1-7** 1mM (2 µL), Water (2 µL). Reaction mixtures were incubated at 60 °C during 1 hour;
2. **Bst 2.0** polymerase (1 µL), Isothermal buffer 10X (1 µL), 3'-OPG-dN*TP **1-7** 1mM (2 µL), Water (3 µL). Reaction mixtures were incubated at 60 °C during 1 hour;
3. **Vent (exo-)** polymerase (1 µL), Thermopol buffer 10X (1 µL), 3'-OPG-dN*TP **1-7** 1mM (2 µL), Water (3 µL). Reaction mixtures were incubated at 60 °C during 1 hour;
4. **Sulfolobus** polymerase (1 µL), Thermopol buffer 10X (1 µL), 3'-OPG-dN*TP **1-7** 1mM (2 µL), Water (3 µL). Reaction mixtures were incubated at 55 °C during 1 hour;
5. **Deep Vent** polymerase (1 µL), Thermopol buffer 10X (1 µL), 3'-OPG-dN*TP **1-7** 1mM (2 µL), Water (3 µL). Reaction mixtures were incubated at 55 °C during 1 hour;
6. **Klenow (exo-)** polymerase (1 µL), NEBuffer 2 10X (1 µL), 3'-OPG-dN*TP **1-7** 1mM (2 µL), Water (3 µL). Reaction mixtures were incubated at 37 °C during 1 hour;
7. **KOD exo Mutant MP1** polymerase (1 µL), KOD DNA polymerase buffer 10X (1 µL), MgCl_2_ 25 mM (1 µL), 3'-OPG-dN*TP **1-7** 1mM (2 µL), Water (2 µL). Reaction mixtures were incubated at 60 °C during 1 hour.

**Preparative scale optimal conditions (100 µL):** DNA Primer MH5 100µM (5 µL), DNA Template MH4A 100µM (7.5 µL), Water (2.5 µL) were mixed and hybridized by incubation program 95 °C (5 minutes) to 62 °C (5 minutes) to 25 °C. Next, other components of the reaction were added depending on DNA polymerase used:

1. **Bst 2.0** polymerase (25 µL), Isothermal buffer 10X (10 µL), 3'-OPG-dN*TP **1-2** 10mM (15 µL), Water (35 µL). Reaction mixtures were incubated at 60 °C during 1 hour;
2. **Deep Vent** polymerase (25 µL), Thermopol buffer 10X (10 µL), 3'-OPG-dN*TP **4** 10mM (15 µL), Water (35 µL). Reaction mixtures were incubated at 55 °C during 1 hour;
3. **Klenow (exo-)** polymerase (25 µL), NEBuffer 2 10X (10 µL), 3'-OPG-dN*TP **3, 4, 6, 7** 10mM (15 µL), Water (35 µL). Reaction mixtures were incubated at 37 °C during 1 hour;


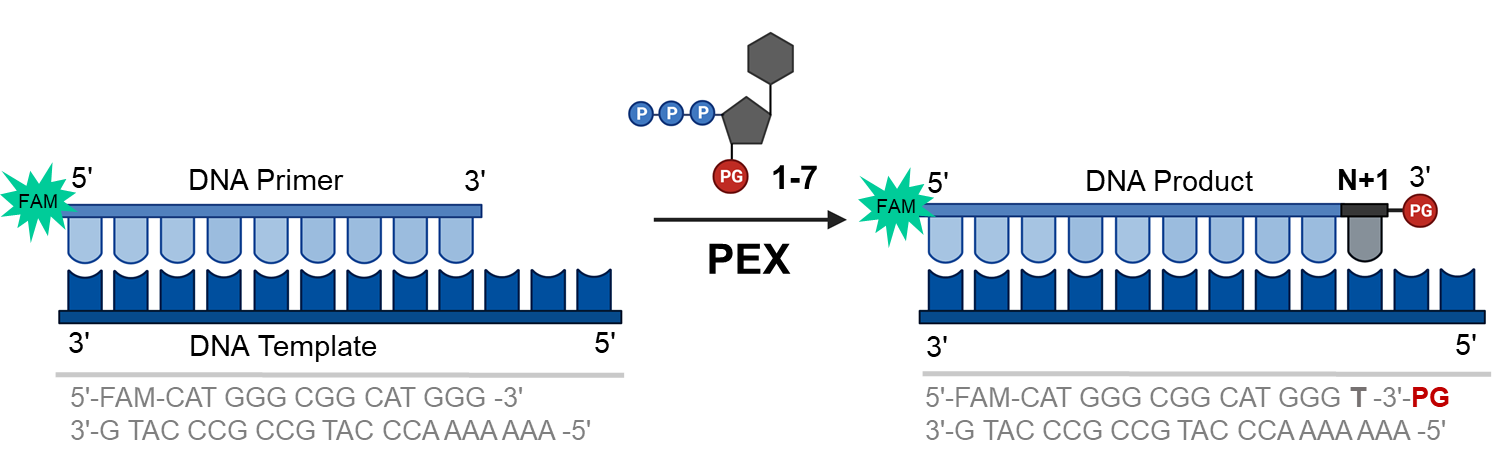


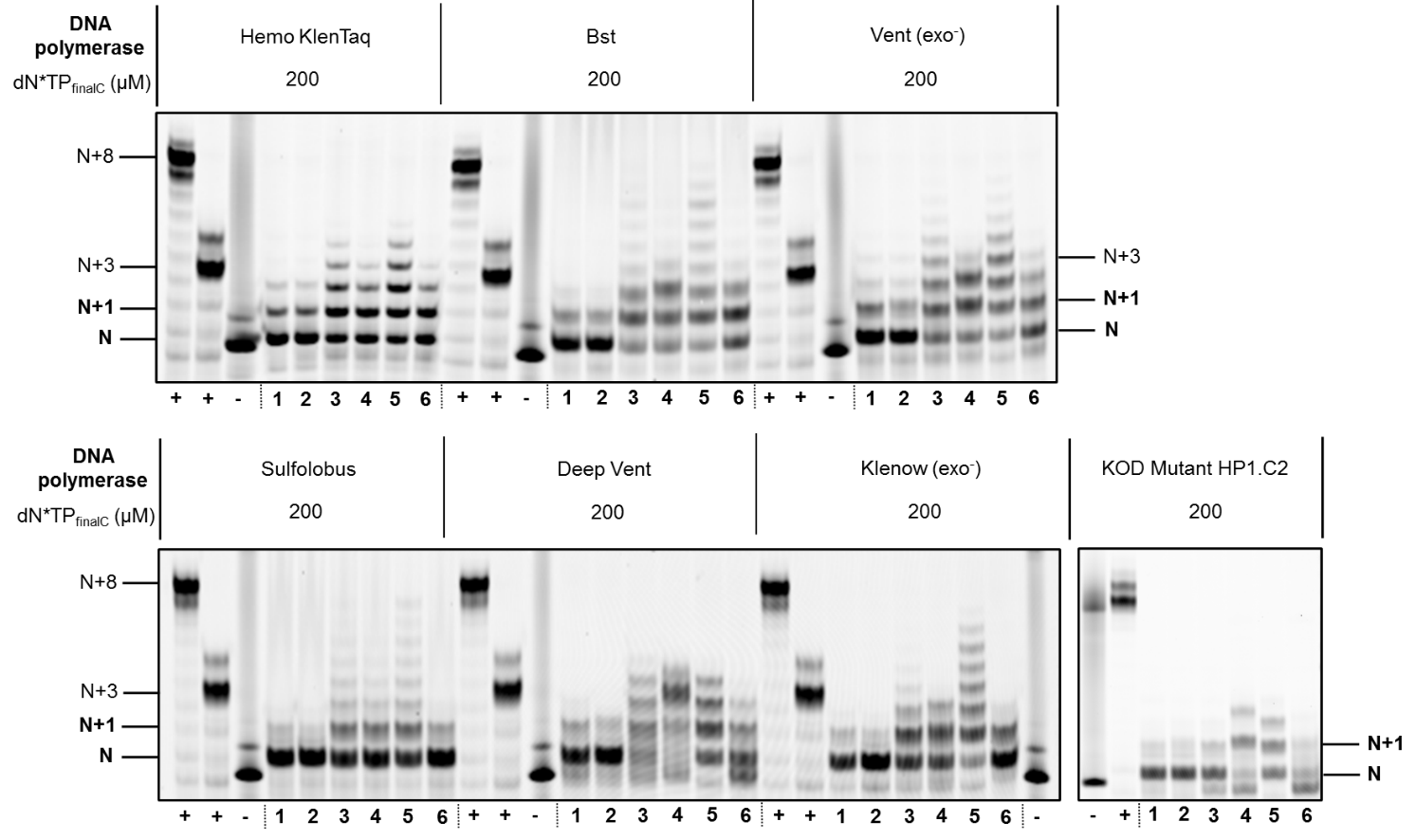


**Supplementary Figure 4a.** Gel images (PAGE 20%) for analysis of PEX reactions. First from the left (**+**) – positive control using dTTP, second from the left (**+**) – positive control using 3’-OH-LNA-dN*TP, (**-**) – negative control in the absence of polymerase, (**1-6**) – 3'-OPG-dN*TPs.


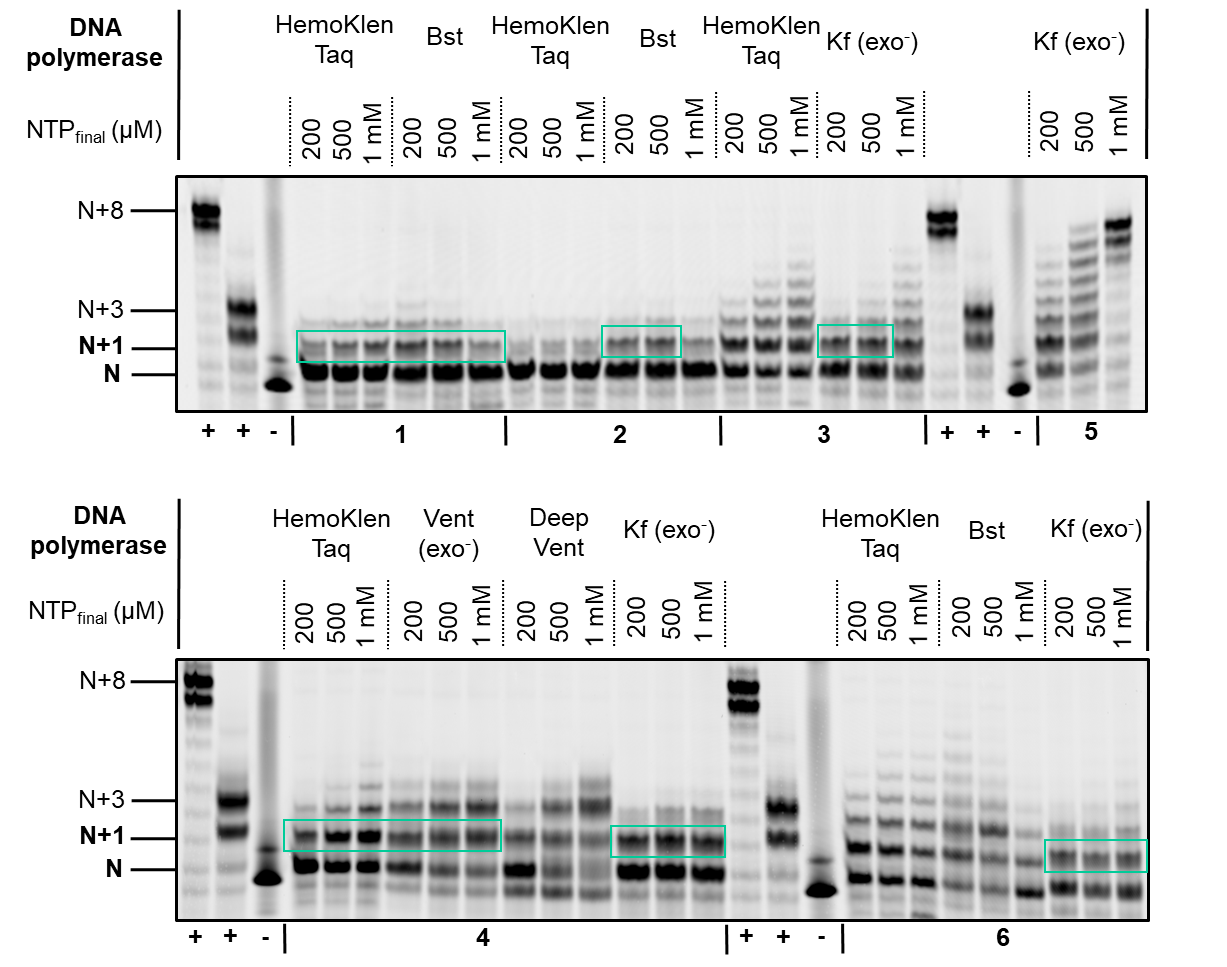


**Supplementary Figure 4b.** Gel images (PAGE 20%) for analysis of PEX reactions. First from the left (**+**) – positive control using dTTP, second from the left (**+**) – positive control using 3’-OH-LNA-dN*TP, (**-**) – negative control in the absence of polymerase, (**1-6**) – 3'-OPG-dN*TPs.


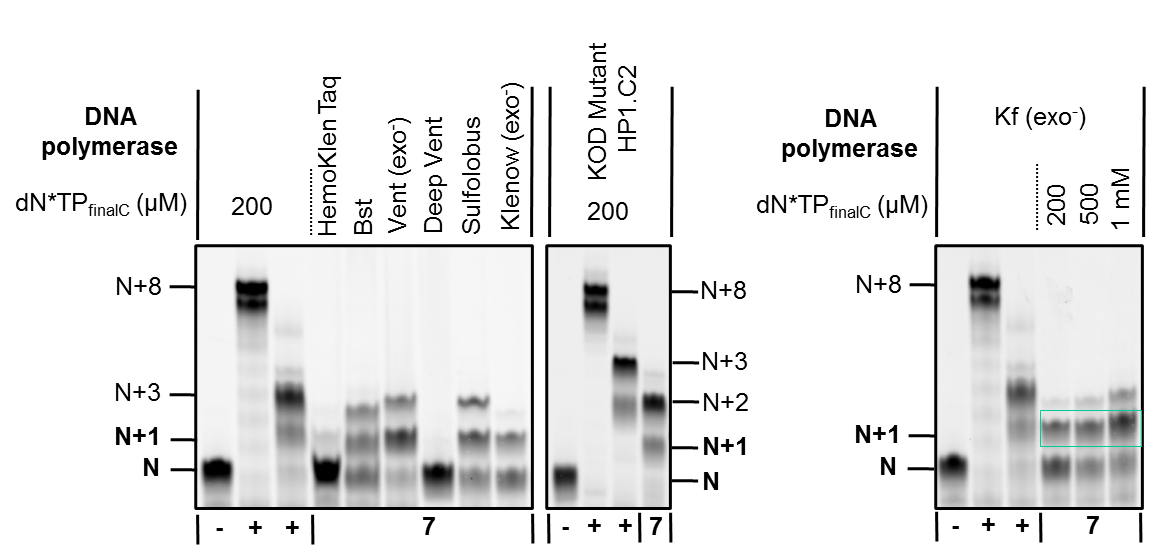


**Supplementary Figure 4c.** Gel images (PAGE 20%) for analysis of PEX reactions. (**+**) – positive control using dTTP, second from the left (**+**) – positive control using 3’-OH-LNA-dN*TP, (**-**) – negative control in the absence of polymerase. **7** – 3'-OCH_2_N_3_-LNA-N*TP.


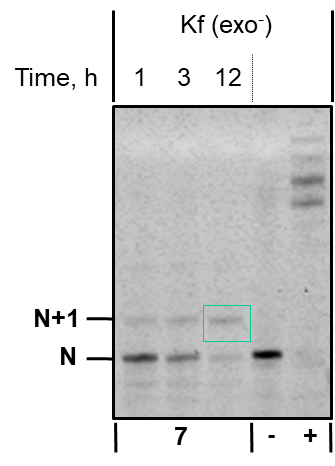


**Supplementary Figure 4d.** Gel image (PAGE 20%) for analysis of PEX reactions carried out with nucleotide **7** in the presence of primer (10 pmoles), template (15 pmoles), modified triphosphates at 100 µM final, and Kf (*exo*^-^) (5 U) at 37°C for 1h, 3h or 12h.. (**+**) – positive control using dTTP, (**-**) – negative control in the absence of polymerase.


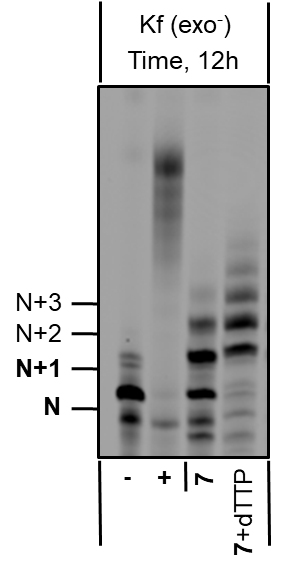


**Supplementary Figure 4e.** Gel image (PAGE 20%) for analysis of PEX reactions using Kf (*exo^-^*) (5U) polymerase in DTT-free reaction and storage buffers for 12h. (**+**) – positive control using dTTP, (**-**) – negative control in the absence of polymerase. **7** – 3'-OCH_2_N_3_-LNA-N*TP. **7**+dTTP – 3'-OCH_2_N_3_-LNA-N*TP used in PEX, then dTTP was added to the reaction mixture and incubated for an additional 1h.

# LCMS analyses of enzymatic reactions

**LC MS settings and method**

Chromatographic separations for LC–MS experiments were performed on a Thermo Scientific™ Vanquish™ Flex Binary UHPLC system (Thermo Fisher Scientific, Reinach, Switzerland).

The column used for all separations was a Waters Aquity Premier BEH C18 Peptide 2.1*50mm 1.7µm 300A (Waters (CH) AG, Baden-Dättwil, Switzerland). The solvents were A: 15mM Amylamine (ALDRICH, W424201 (inhouse re-distilled), CAS 110-58-7, SIGMA-ALDRICH CHEMIE GMBH (CH), Buchs Switzerland) and 50mM 1,1,1,3,3,3-HEXAFLUORO-2-PROPANOL (HFIP, ACROS ORGANICS, ACR14754 (99.5+%, PURE), CAS 920-66-1, ThermoFisher Scientific, Reinach Switzerland) in water (Milli-Q® IQ 7000, Millipore, Merck & Cie, Schafhausen, Switzerland) and B: methanol/acetonitrile (9/1;v/v) both gradient grade. Both solvents contained 1µM Ethylenediaminetetraacetic acid disodium salt dihydrate (EDTA), SIGMA, E5134-100G ( 99.0-101.0%, titration)), CAS 6381-92-6, SIGMA-ALDRICH CHEMIE GMBH (CH), Buchs Switzerland helping to supress metal adducts forming in the mass spectrometer. A flow rate of 400 μL min−1 was applied, the column compartment was held at 80°C. The gradient system was A vs B starting at 5%B with a raise to 20%B within 3 min, and reaching 35%B at 25.5min followed by a short step to 100%B before coming back to the initial 5%B for reinjection.

For mass spectrometric data acquisition, a Thermo Scientific™ Fusion Lumos™ Hybrid Quadrupole-Orbitrap mass spectrometer equipped with a heated electrospray ionization-II (HESI-II) probe in a standard Thermo Scientific™ Ion Max™ ion source (Thermo Fisher Scientific, San José, CA, USA) was used. Data acquisition was performed with Thermo Scientific™ Xcalibur 4.5. HR LC–MS measurements were performed under Thermo Scientific™ Xcalibur™ Orbitrap Fusion Lumos Tune Application 3.5. The mass range was 600-2000 Da at a resolution of 120K. In addition, the full DAD but also a 260nm UV trace were acquired.

MS raw files were exported to ThermoFisher Scientific BioPharma Finder (BPF) 5.1 software and analyzed using the ReSpect algorithm via the sliding window deconvolution feature either against the full structure of the compounds or just against the molecular mass. All data evaluation was done based on the Thermo Scientific BioPharma Finder User Guide Software Version 5.1, XCALI-98492 Revision A, July 2022.

**5a. TdT reactions**

| **TdT reaction conditions** | **m/z calculated** | **m/z found** | **Interpretation of the reaction product** |
| --- | --- | --- | --- |
| TdT of **4** | 6614,1832 | 6574,1833 | **4** was added to the **ON1** primer; allyl protecting group was cleaved; the found mass correspond to 3’-OH product (m/z calc. 6574,1519). |
| TdT of **6** | 6588,1676 | 6588,1960 | **6** was added to the **ON1** primer and detected as the main product in the reaction. |
| TdT of **7** | 6629,1690 | 6574,1938 | **7** was added to the **ON1** primer; azidomethyl protecting group was cleaved; the found mass correspond to 3’-OH product (m/z calc. 6574,1519). |

**Supplementary Table 1.** Summary of LCMS analysis of TdT reactions.

**LCMS analysis of TdT reaction of 3’-OAllyl-dN*TP 4.**


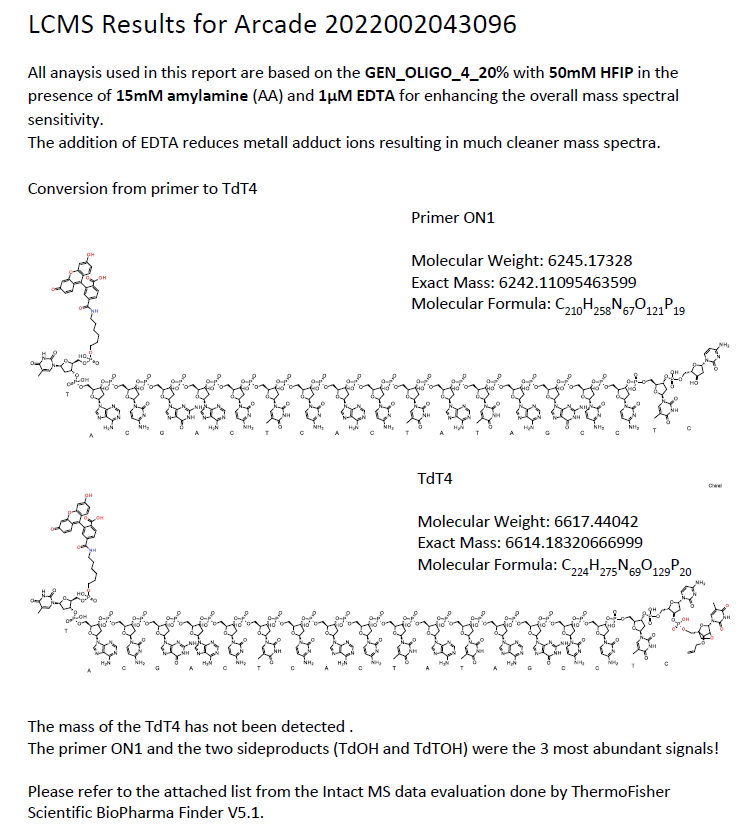


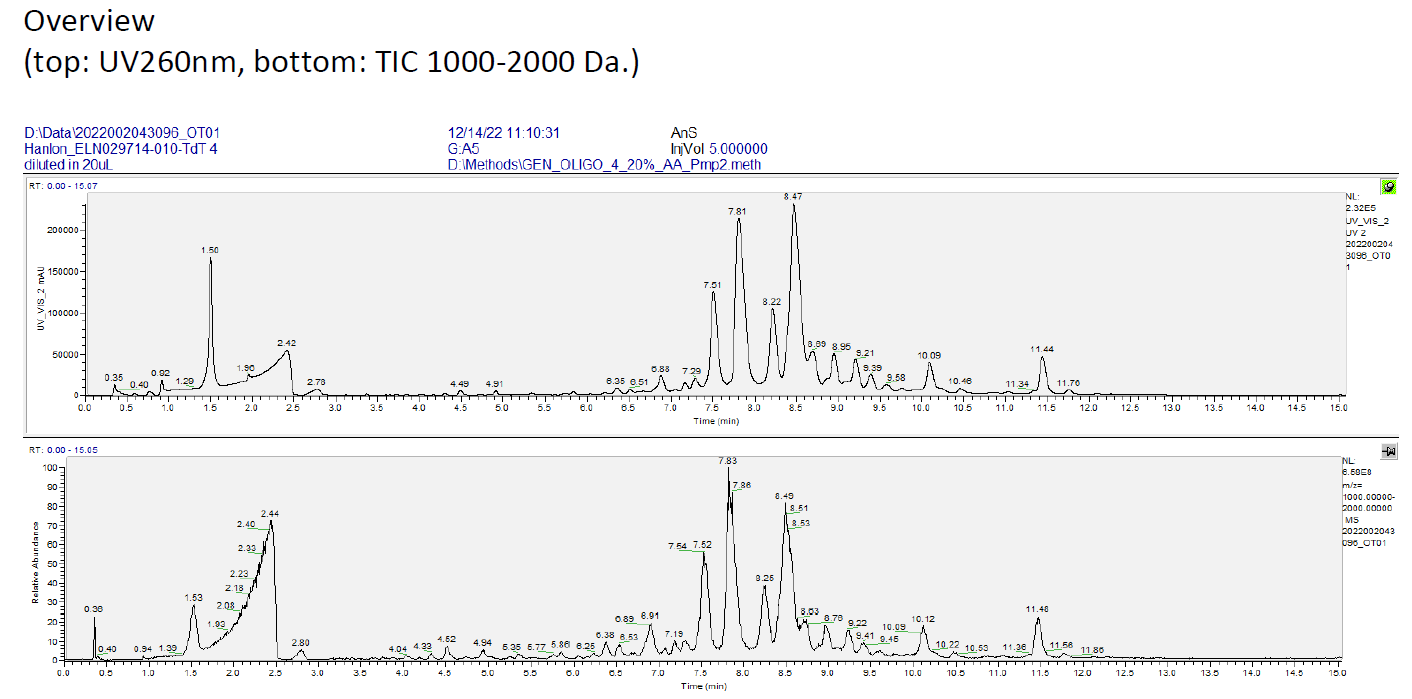


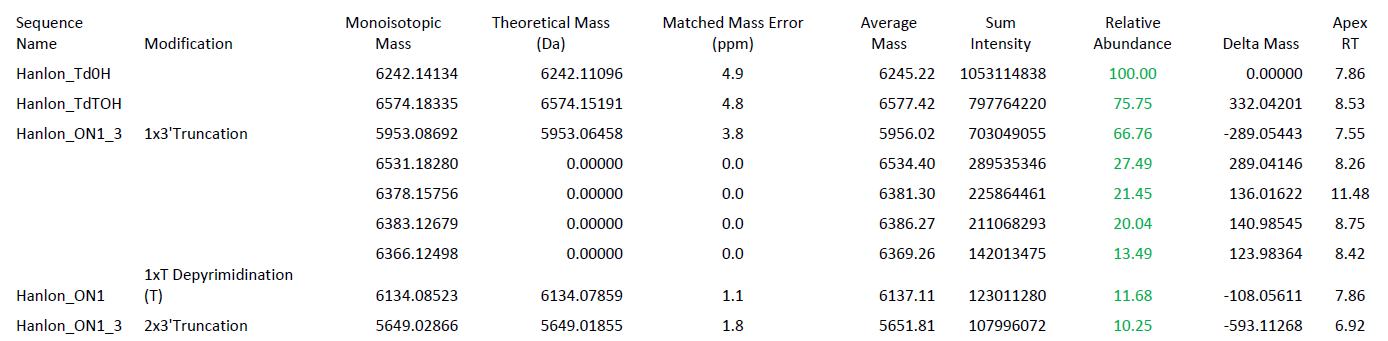


**LCMS analysis of TdT reaction of 3’-OMe-dN*TP 6.**


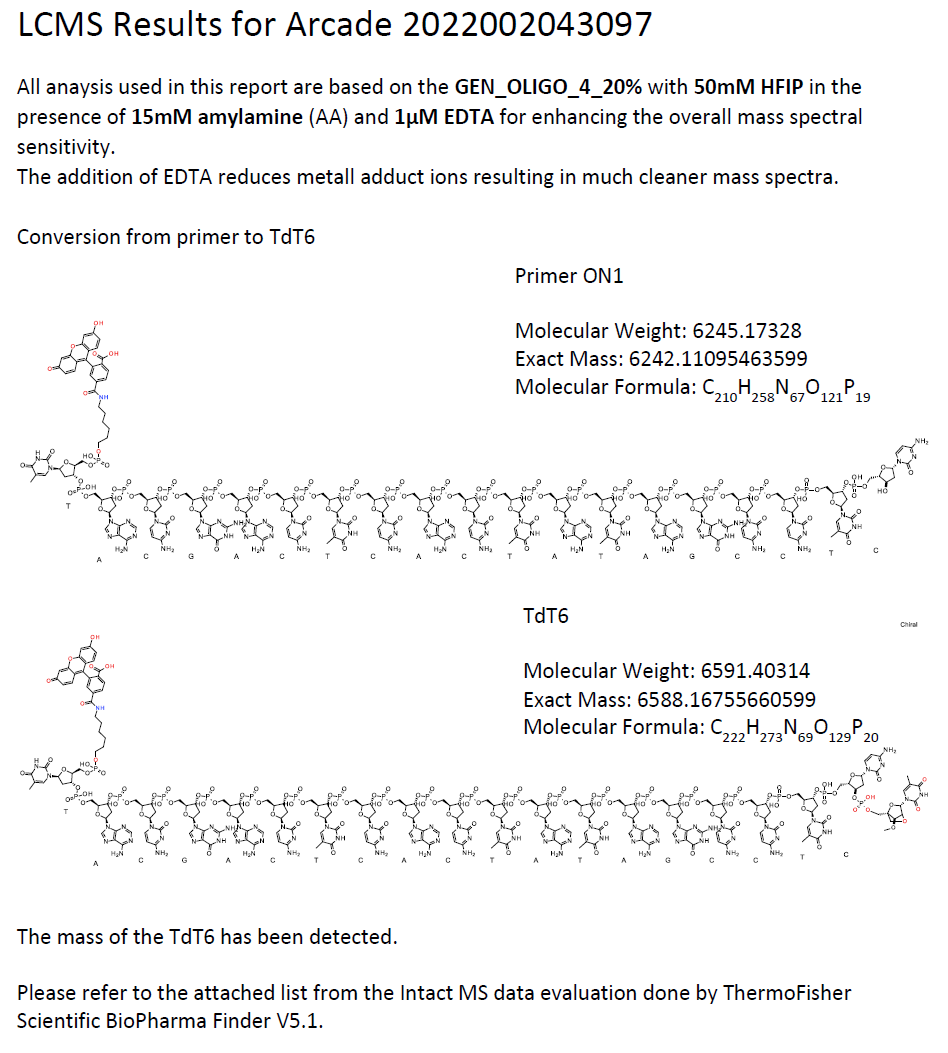


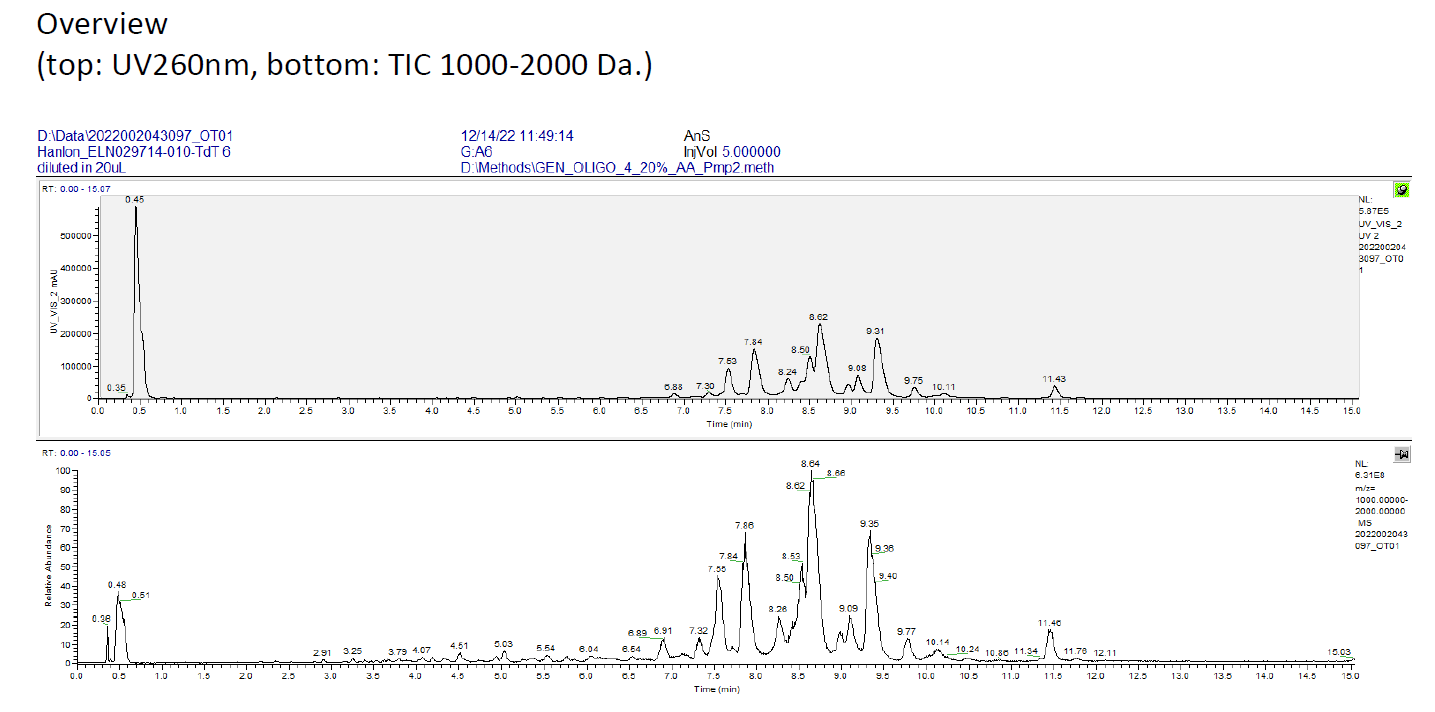


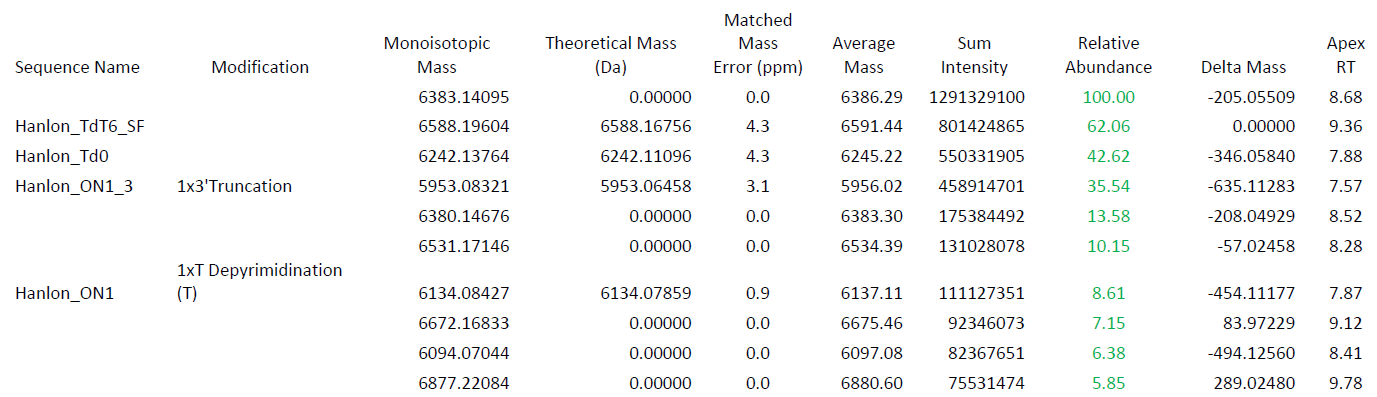


**LCMS analysis of TdT reaction of 3’-CH_2_N_3_-dN*TP 7.**


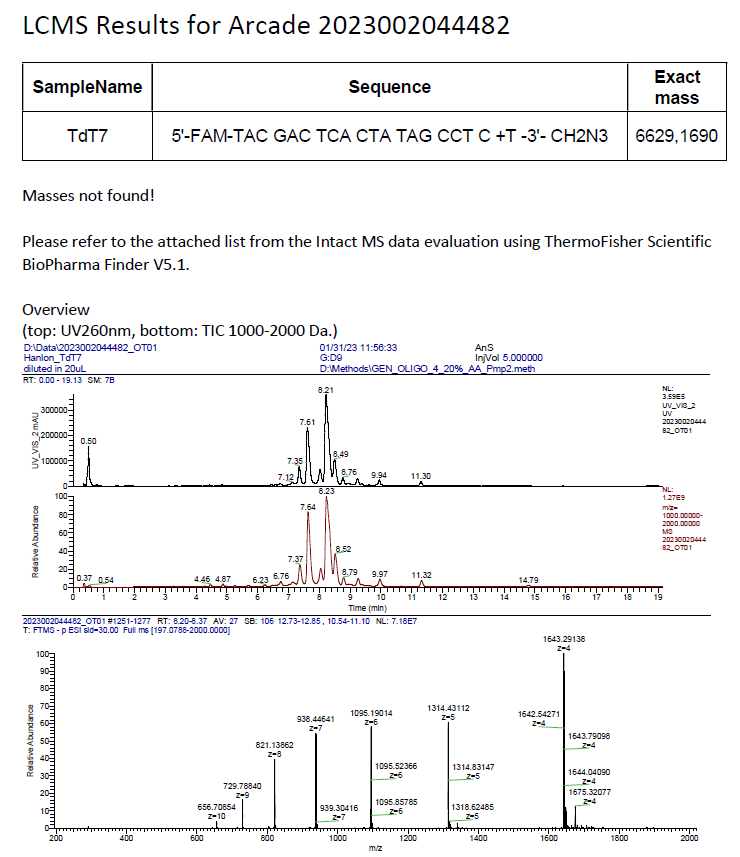


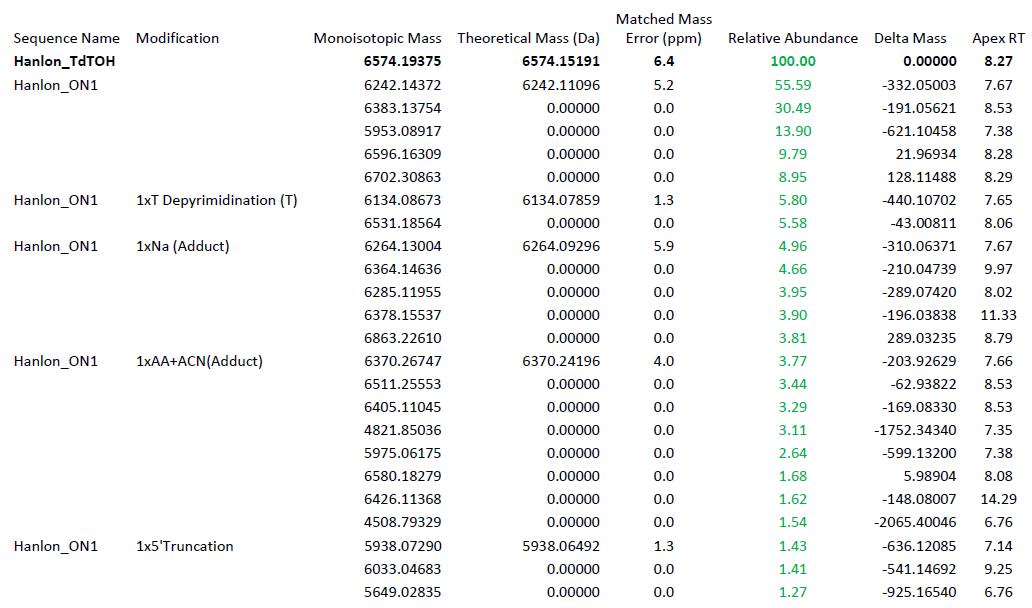


**5b. PUP reactions**

| **PUP reaction conditions** | **m/z calculated** | **m/z found** | **Interpretation of the reaction product** |
| --- | --- | --- | --- |
| PUP of **3** | 6649,0175 | 6649,0473 | **3** was added to the **FLA RNA** primer and detected as the main product in the reaction. |
| PUP of **4** | 6677,0124 | 6677,0316 | **4** was added to the **FLA RNA** primer and detected as the main product in the reaction. |
| PUP of **5** | 6664,0032 | 6664,0347 | **5** was added to the **FLA RNA** primer and detected as the main product in the reaction. |
| PUP of **6** | 6650,9967 | 6651,0190 | **6** was added to the **FLA RNA** primer and detected as the main product in the reaction. |
| PUP of **7** | 6691,9981 | 6637,0165 | **7** was added to the **FLA RNA** primer; azidomethyl protecting group was cleaved; the found mass correspond to 3’-OH product (m/z calc. 6636,9811). |

**Supplementary Table 2.** Summary of LCMS analysis of PUP reactions.

**LCMS analysis of PUP reaction of 3’-Allyl-dN*TP 3.**


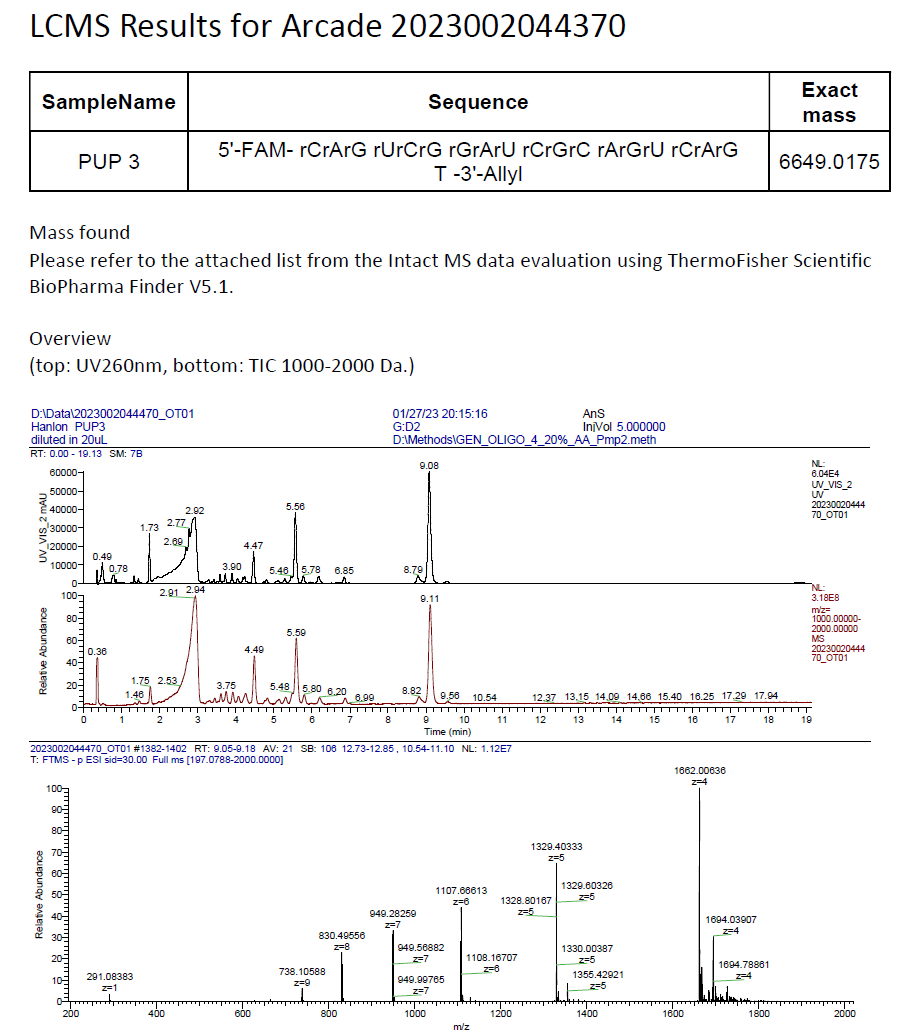


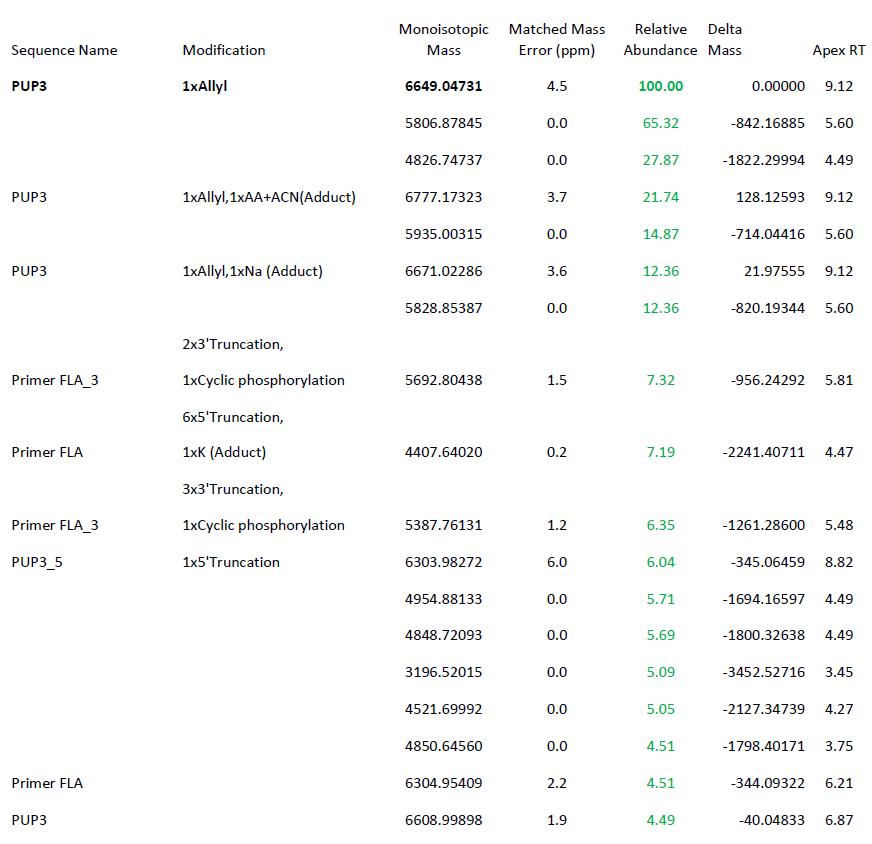


**LCMS analysis of PUP reaction of 3’-Allyl-dN*TP 4.**


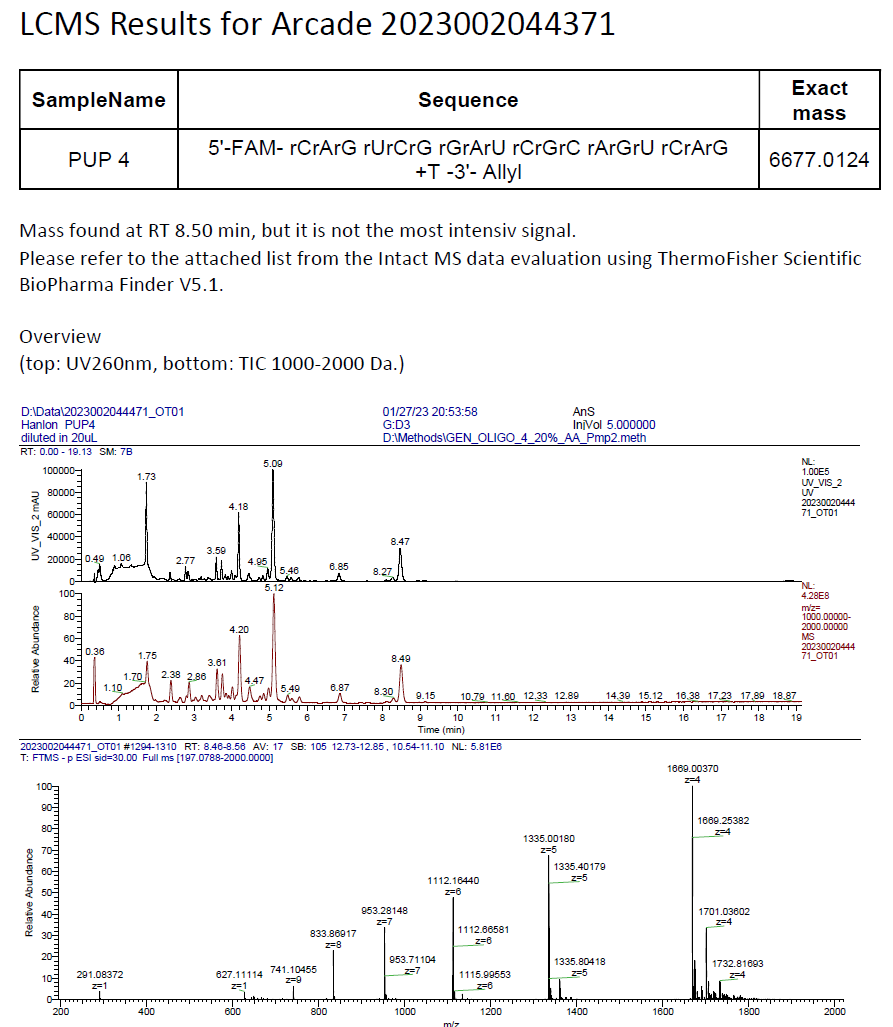


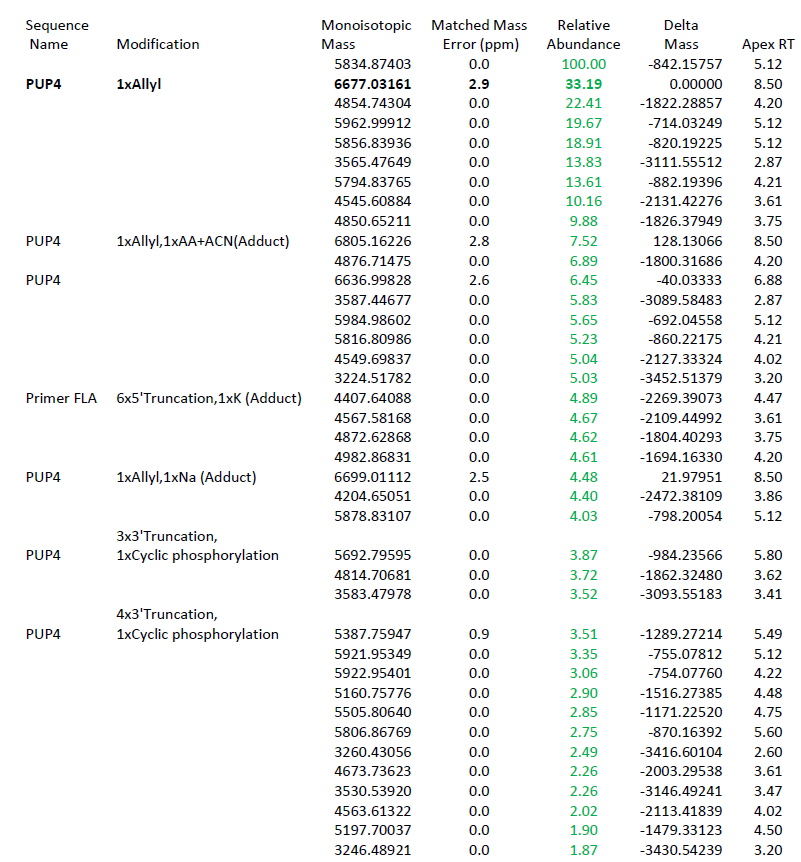


**LCMS analysis of PUP reaction of 3’-Azidomethyl-dN*TP 5.**


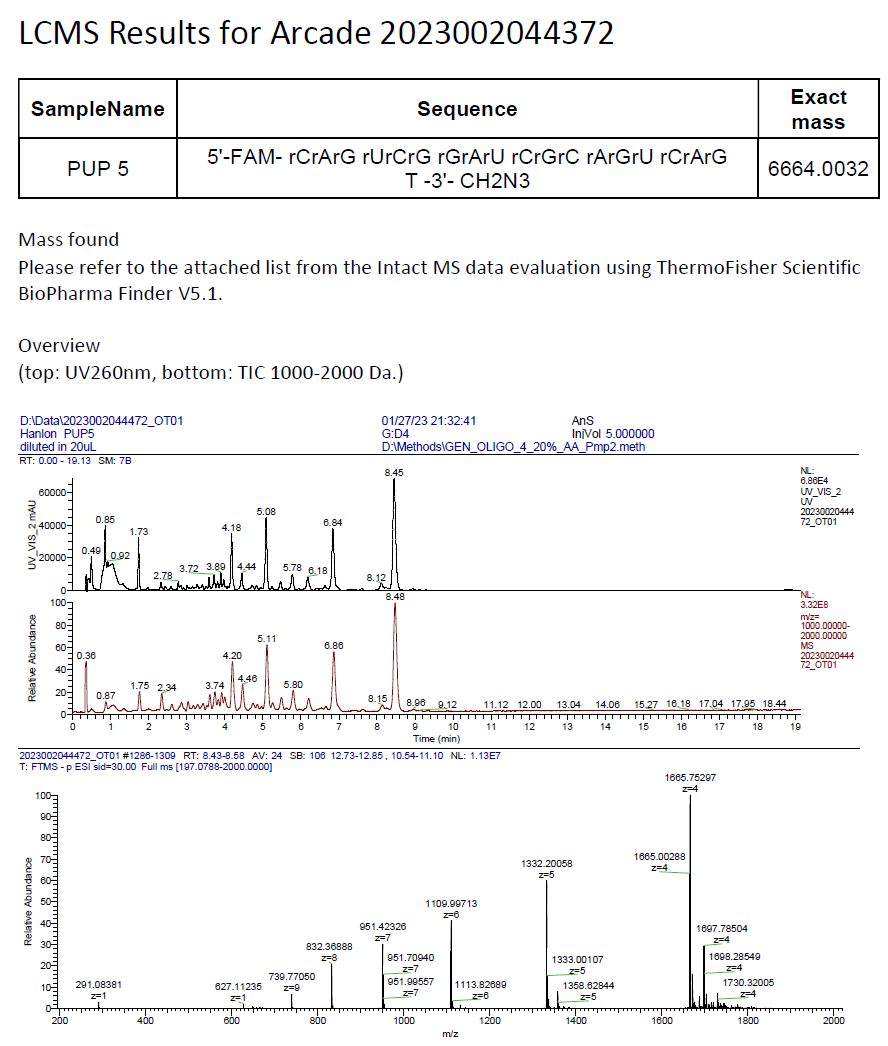


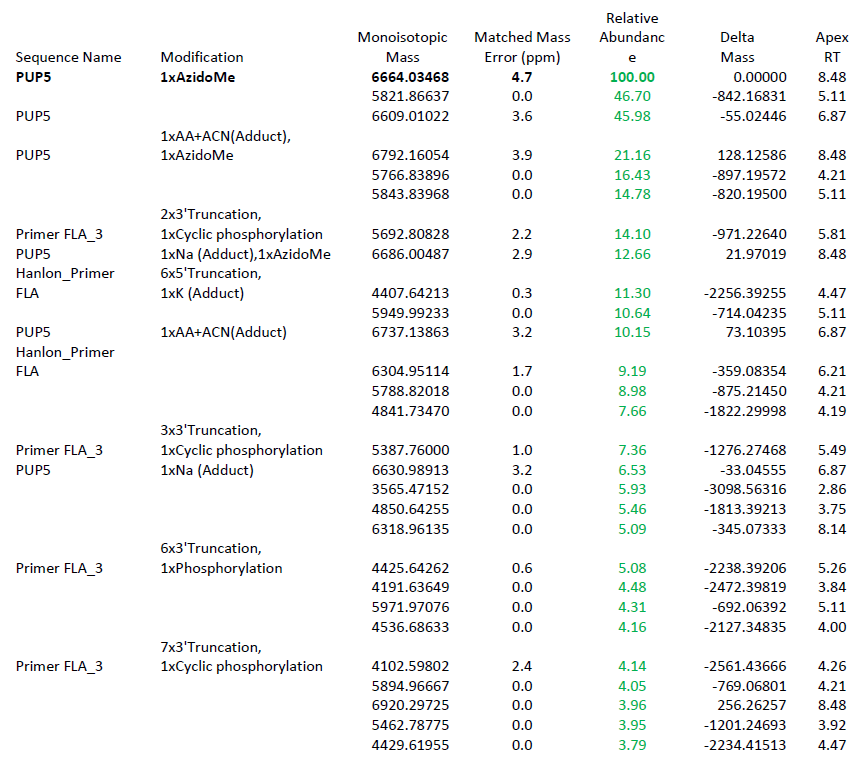


**LCMS analysis of PUP reaction of 3’-Methyl-dN*TP 6.**


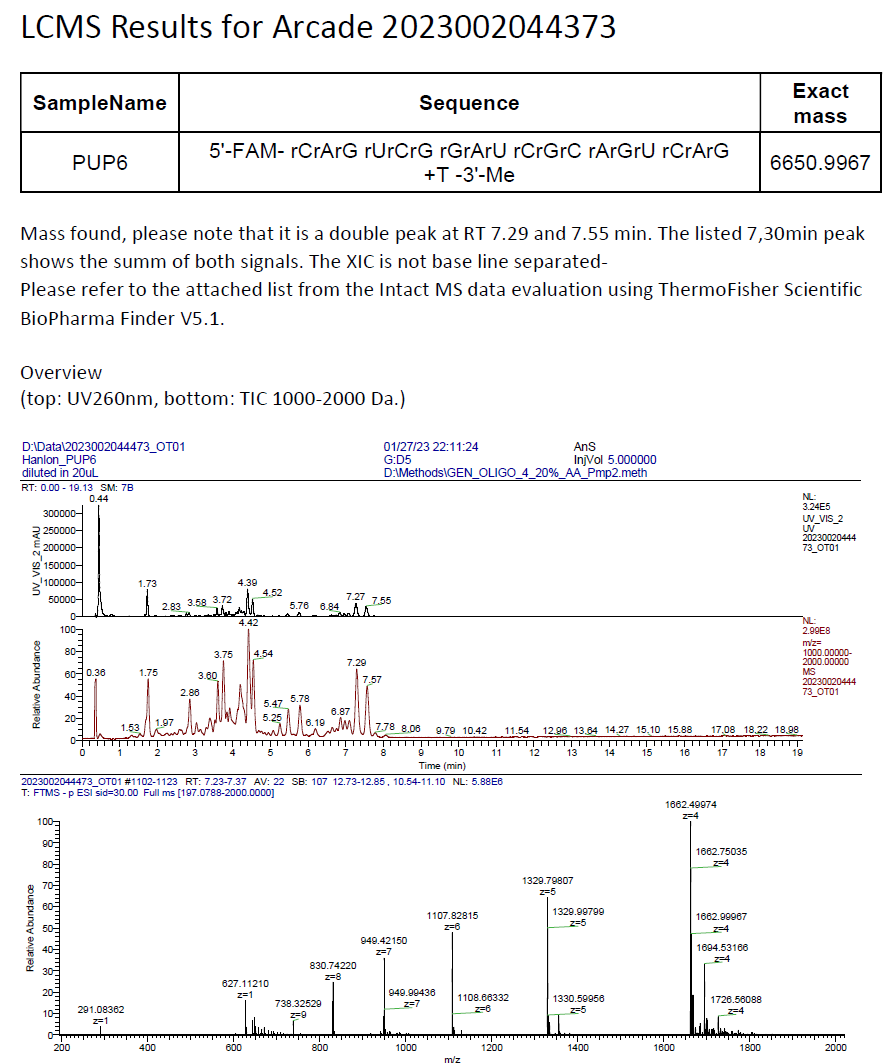


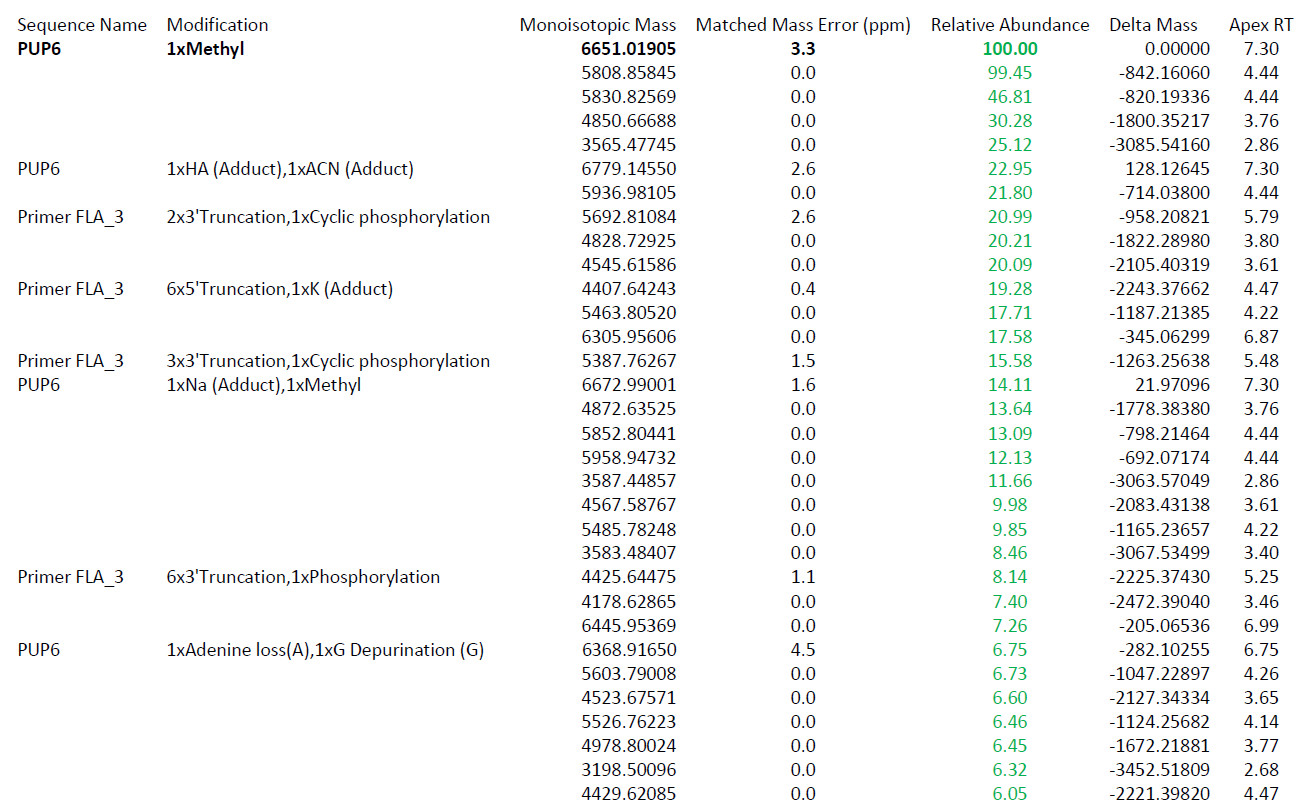


**LCMS analysis of PUP reaction of 3’-Azidomethyl-dN*TP 7.**


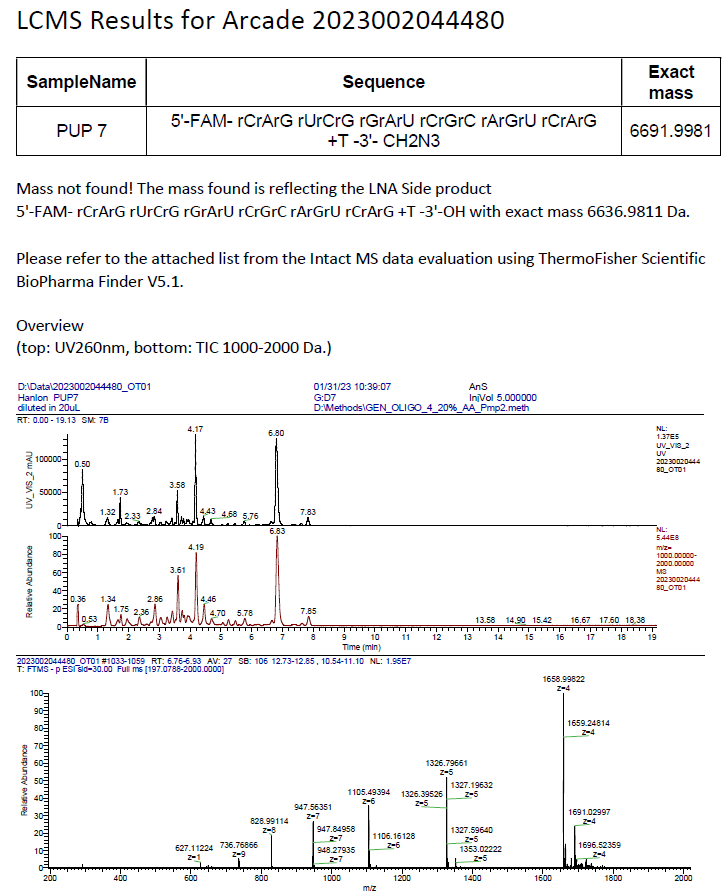


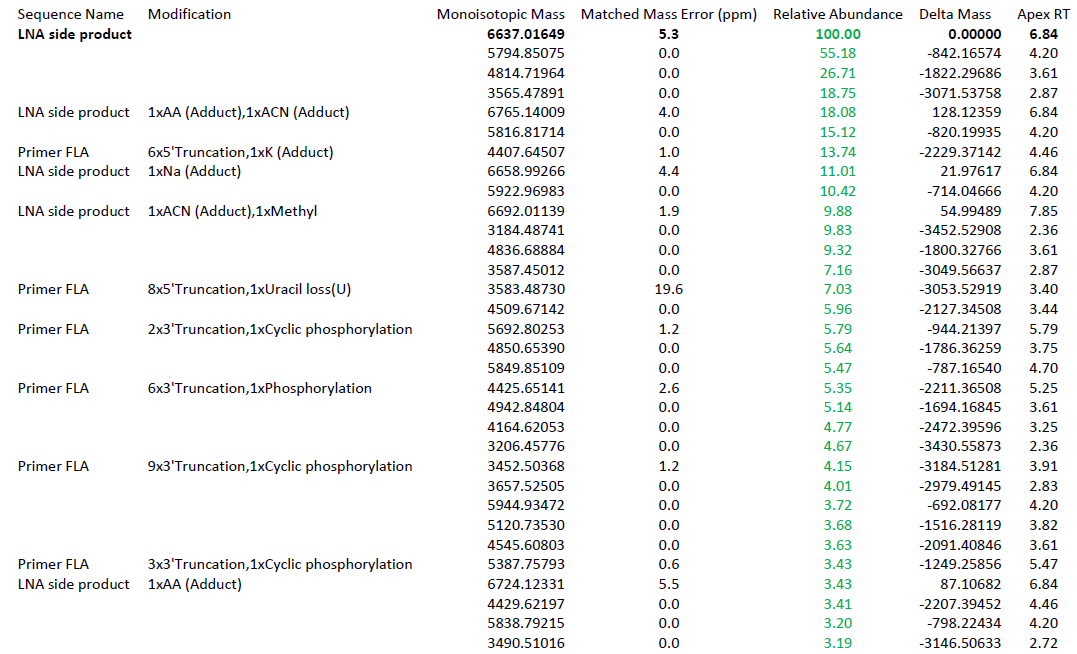


**5c. PEX reactions**

| **PEX reaction conditions** | **m/z calculated** | **m/z found** | **Interpretation of the reaction product** |
| --- | --- | --- | --- |
| PEX of **1** | 5659,0489 | 6743,18647 | No product was detected; phosphorylation of template MH4A was observed (m/z calc. 6743,14962). |
| PEX of **2** | 5687,0438 | 6743,18848 | No product was detected; phosphorylation of template MH4A was observed (m/z calc. 6743,14962). |
| PEX of **3** | 5553,0070 | 5512,99895 | **3** was added to primer MH5; allyl protecting group was cleaved. 3’-OH product was detected (m/z calc. 5512,9757). |
| PEX of **4** | 5581,0019 | 5540,98604 | **4** was added to primer MH5; allyl protecting group was cleaved. 3’-OH product was detected (m/z calc. 5540,9706). |
| PEX of **6** | 5554,9863 | 6743,18674 | No product was detected; phosphorylation of template MH4A was observed (m/z calc. 6743,14962). |
| PEX of **7** | 5595,9877 | 5541,0007 | **7** was added to primer MH5; azidomethyl protecting group was cleaved. 3’-OH product was detected (m/z calc. 5540,9706). |

**Supplementary Table 3.** Summary of LCMS analysis of PEX reactions.

**LCMS analysis of PEX reaction of 3’-Mesitoyl-dN*TP 1.**

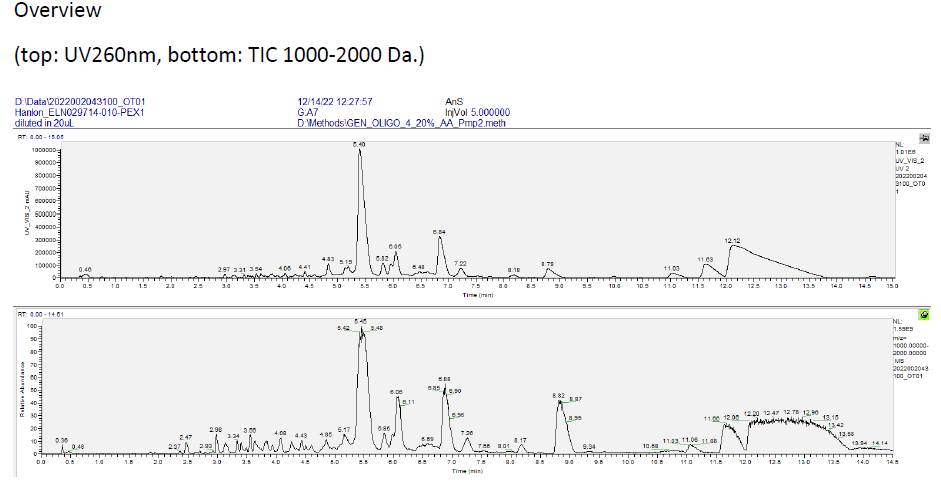


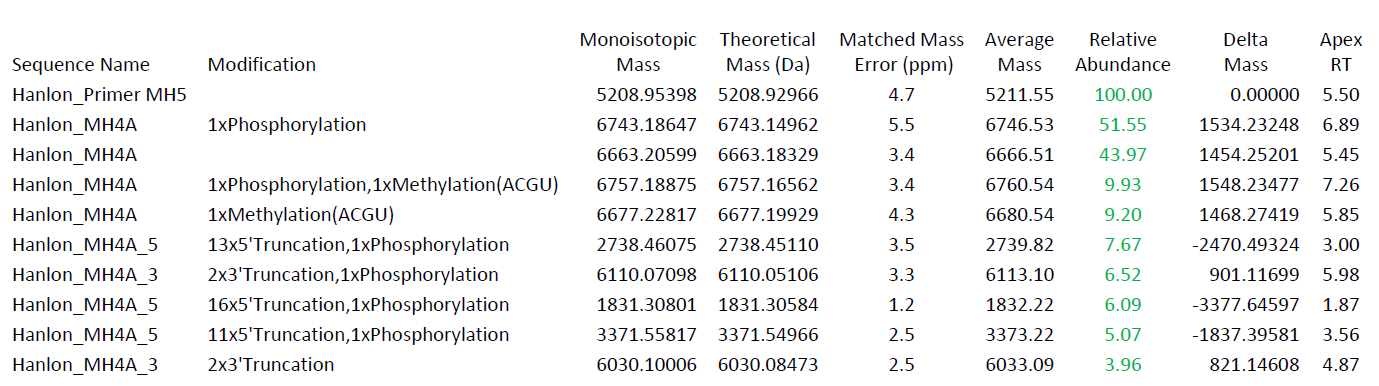


**LCMS analysis of PEX reaction of 3’-Mesitoyl-dN*TP 2.**


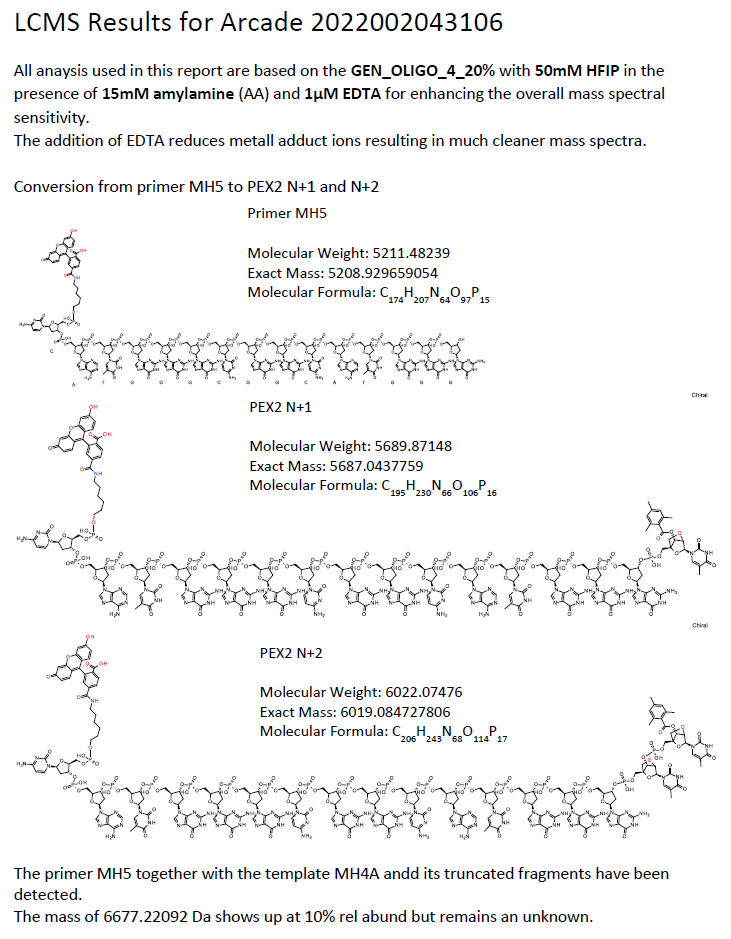


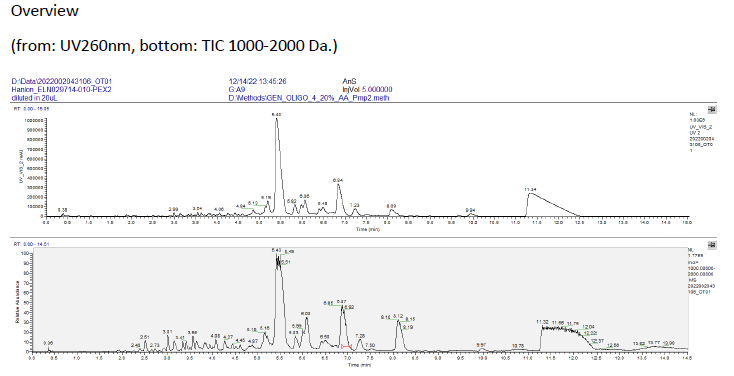


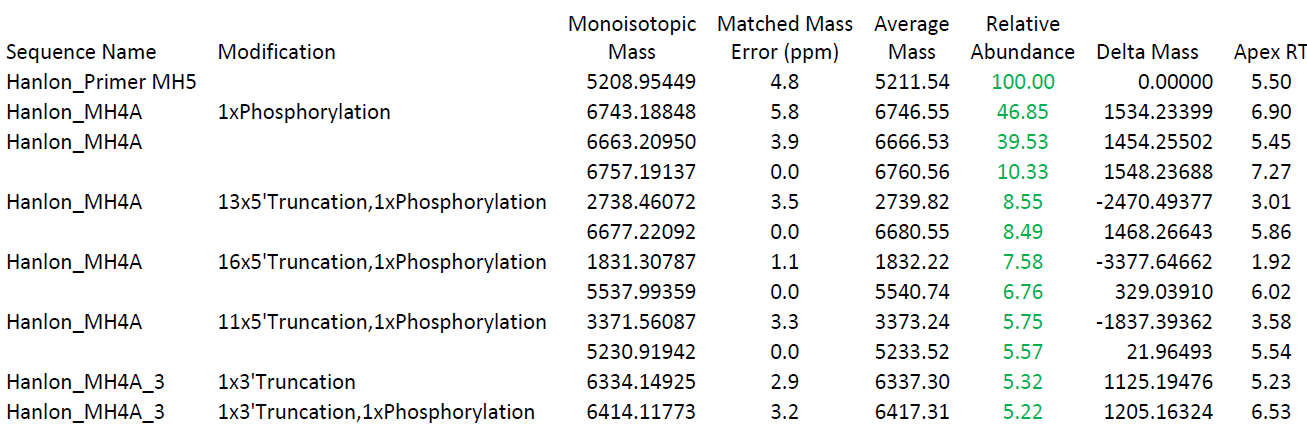


**LCMS analysis of PEX reaction of 3’-Allyl-dN*TP 3.**


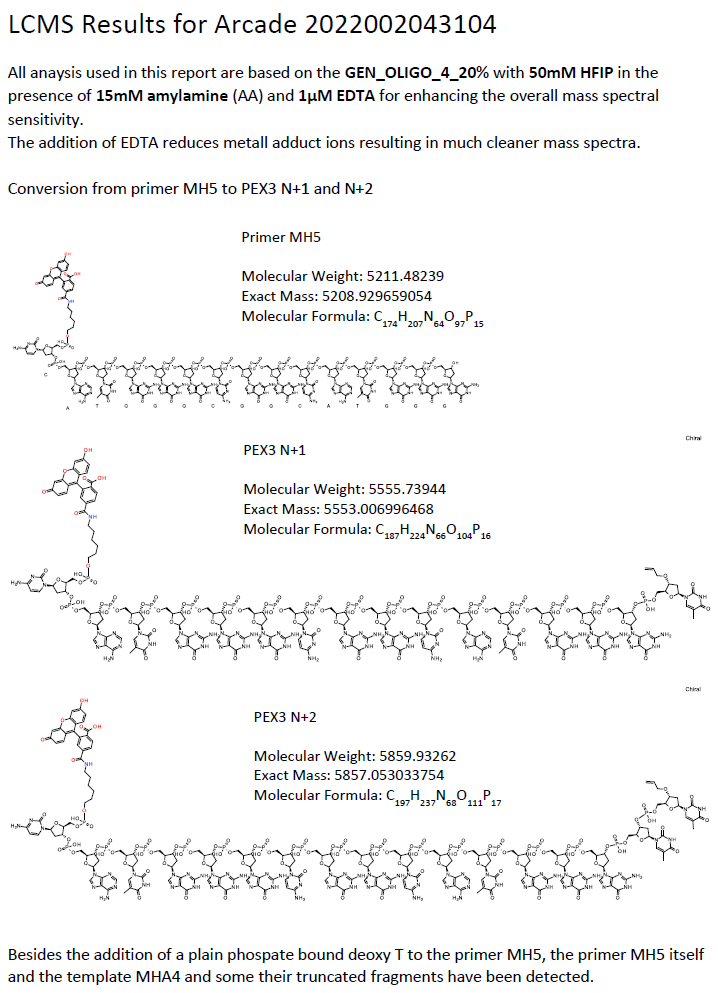


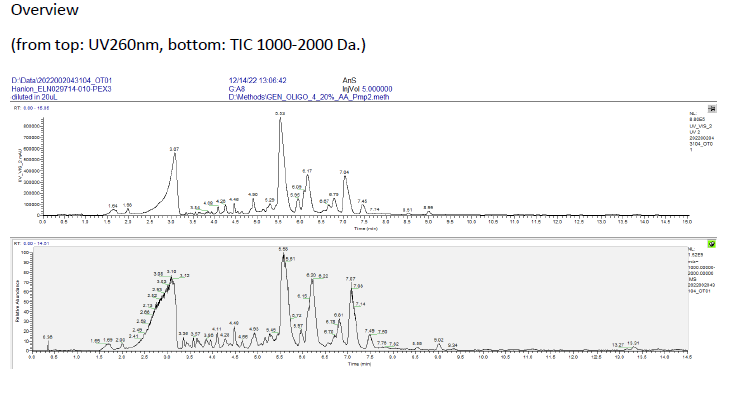


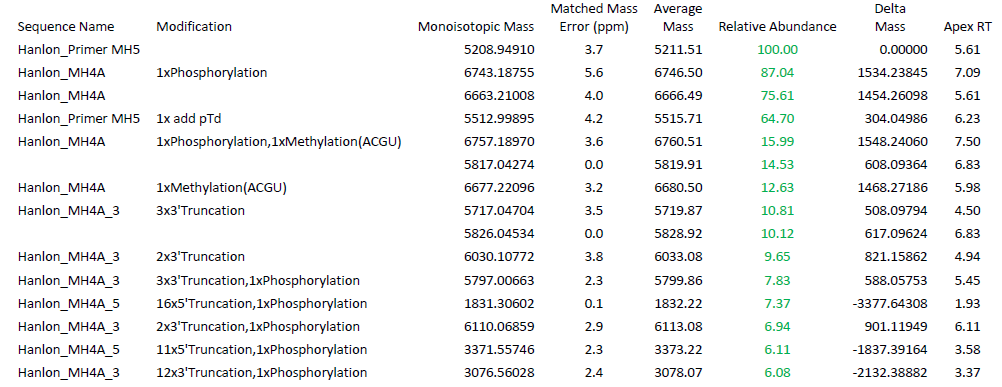


**LCMS analysis of PEX reaction of 3’-Allyl-dN*TP 4.**

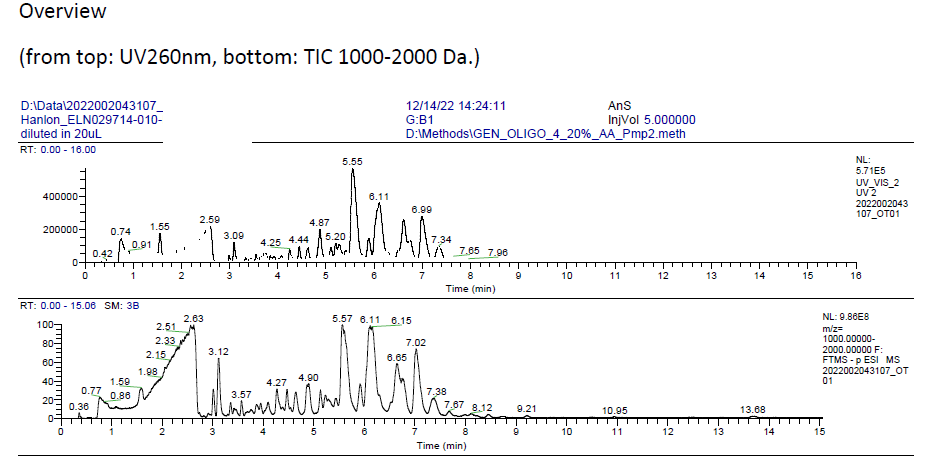


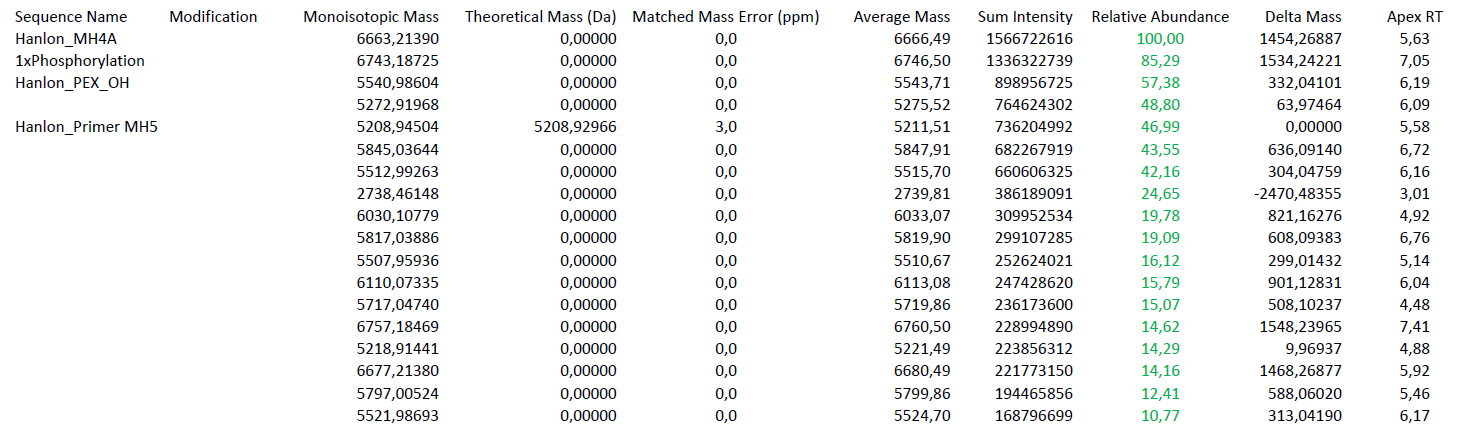


**LCMS analysis of PEX reaction of 3’-Me-dN*TP 6.**


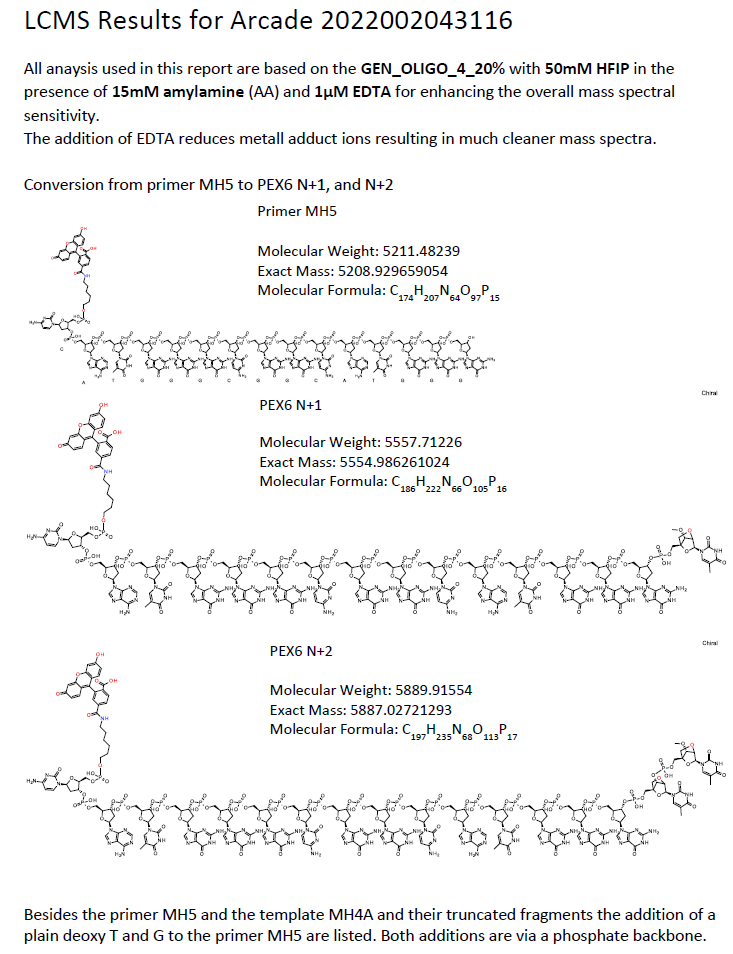

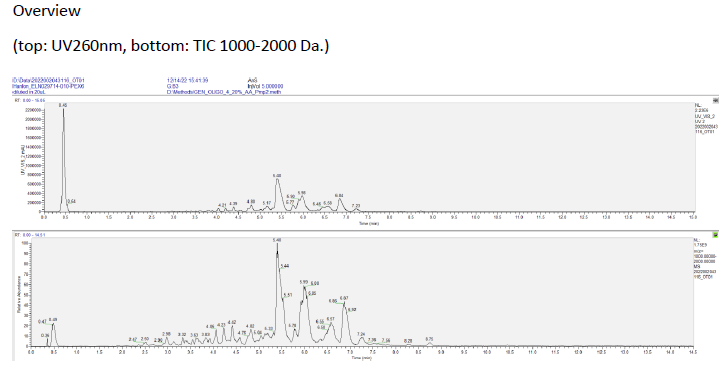


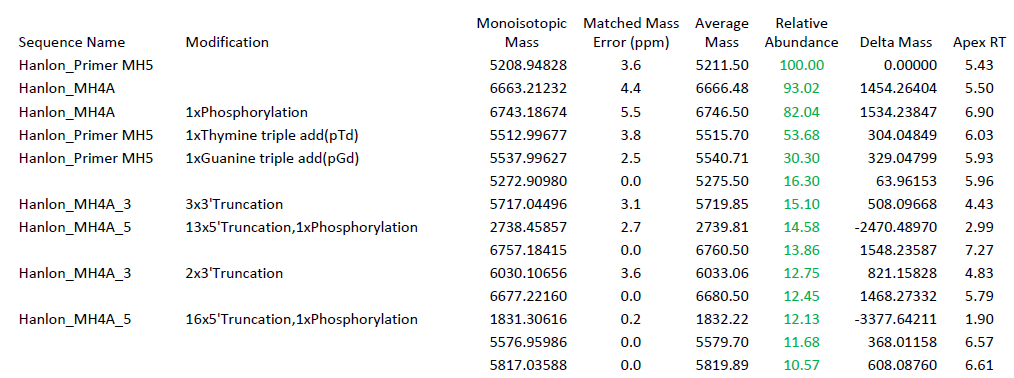


**LCMS analysis of PEX reaction of 3’-CH_2_N_3_-dN*TP 7.**


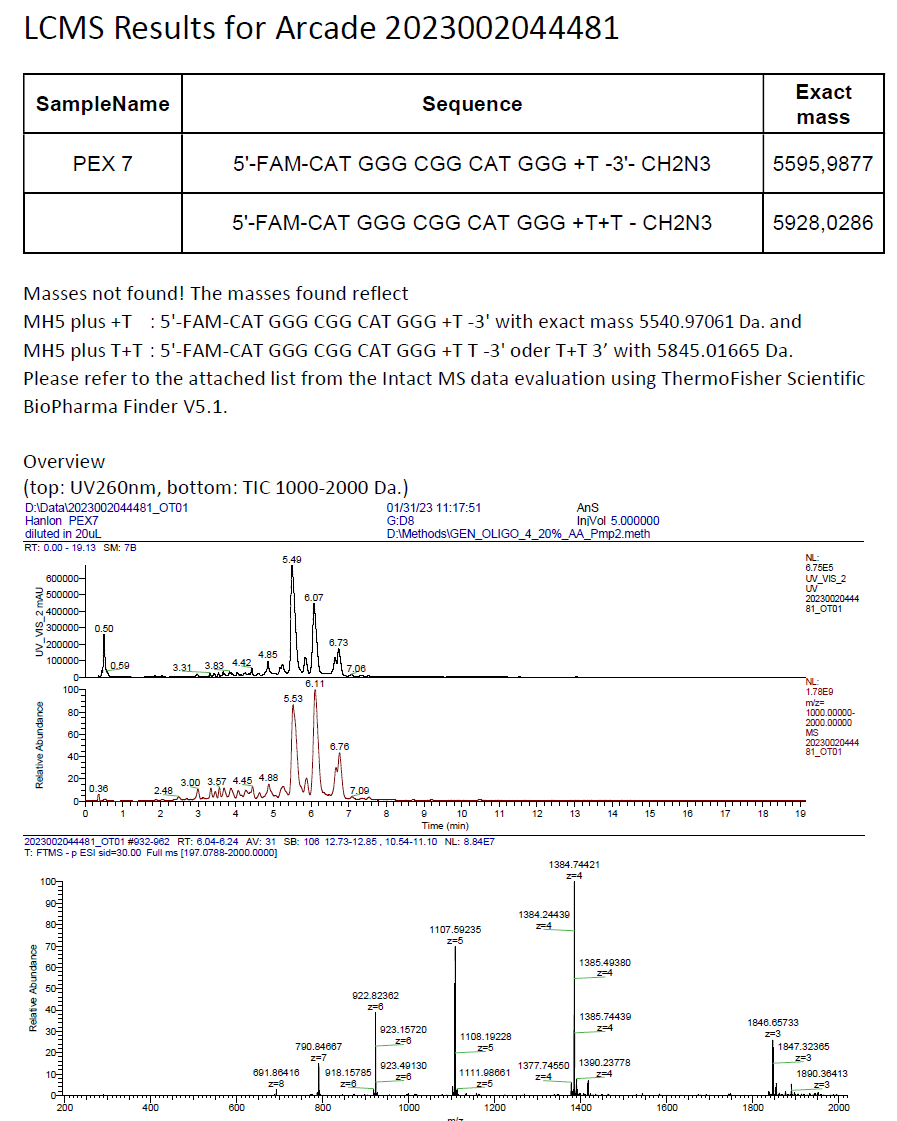


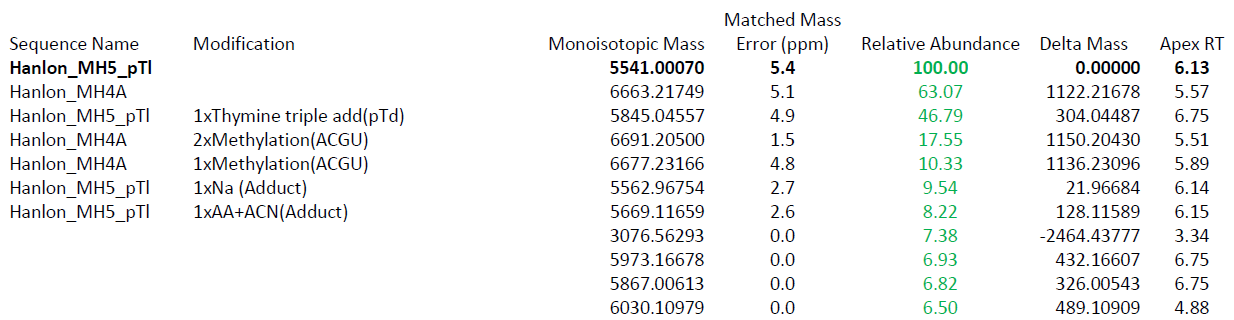

Supplement: Supplementary file 1 [file DataSheet1.docx]
